# Strategic advances in Vat Photopolymerization for 3D printing of calcium phosphate-based bone scaffolds: A review

**DOI:** 10.1016/j.bioactmat.2025.05.001

**Published:** 2025-06-27

**Authors:** Roberto Fagotto-Clavijo, Irene Lodoso-Torrecilla, Anna Diez-Escudero, Maria-Pau Ginebra

**Affiliations:** a Biomaterials, Biomechanics and Tissue Engineering (BBT), Department of Materials Science and Engineering, Universitat Politècnica de Catalunya (UPC) and Institute for Research and Innovation in Health (IRIS), Av. Eduard Maristany, 16, Barcelona, 08019, Spain; b Barcelona Research Centre in Multiscale Science and Engineering, Universitat Politècnica de Catalunya (UPC), Av. Eduard Maristany, 16, Barcelona, 08019, Spain; c CIBER de Bioingeniería, Biomateriales y Nanomedicina (CIBER-BBN), Instituto de Salud Carlos III, Spain; d Institute for Bioengineering of Catalonia (IBEC), The Barcelona Institute of Science and Technology, Baldiri Reixac 10-12, 08028, Barcelona, Spain

**Keywords:** Additive manufacturing, 3D printing, Vat polymerization, Hydroxyapatite, Scaffold, Bone regeneration

## Abstract

3D-printing has emerged as a leading technology for fabricating personalized scaffolds for bone regeneration. Among the 3D-printing technologies, vat photopolymerization (VP) stands out for its high precision and versatility. It enables the creation of complex, patient-specific scaffolds with advanced pore architectures that enhance mechanical stability and promote cell growth, key factors for effective bone regeneration. This review provides an overview of the advances made in vat photopolymerization printing of calcium phosphates, covering both the fabrication of full ceramic bodies and polymer-calcium phosphate composites. The review examines key aspects of the fabrication process, including slurry composition, architectural design, and printing accuracy, highlighting their impact on the mechanical and biological performance of 3D-printed scaffolds. The need to tailor porosity, pore size, and geometric design to achieve both mechanical integrity and biological functionality is emphasized by a review of data published in the recent literature. This review demonstrates that advanced geometries like Triply Periodic Minimal Surfaces and nature-inspired designs, achievable with exceptional precision by this technology, enhance mechanical and osteogenic performance. In summary, VP's versatility, driven by the diversity of material options, consolidation methods, and precision opens new horizons for scaffold-based bone regeneration.

## Highlights

• Vat photopolymerization excels in the fabrication of precise and personalized 3D calcium phosphate-based scaffolds.
• Vat photopolymerization supports fabricating both full ceramic and composite calcium phosphate scaffolds.
• The advanced pore designs enabled by Vat photopolymerization promote efficient bone regeneration.
• Vat Photopolymerization enables the fabrication of complex calcium phosphate scaffolds with tailored mechanical properties.

## Graphical abstract

**Figure:**
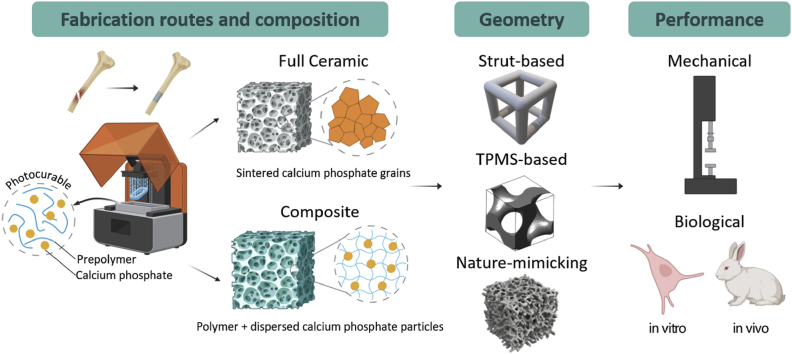

## Teaser

Structured abstractPurpose: 3D printing has steadily garnered increasing popularity for fabricating bone scaffolds. In the last years, vat photopolymerization has emerged as a groundbreaking 3D printing technique for calcium phosphate scaffolds, owing to its high versatility, exceptional printing precision, and material options. This review aims to provide a comprehensive and novel analysis of the advances in vat photopolymerization of calcium phosphate-based scaffolds for bone regeneration, highlighting innovative approaches and strategies.Procedure: This review was conducted through an advanced bibliographic search using Scopus, Web of Science, and PubMed. The search focused on a combination of the following areas of interest: vat photopolymerization techniques, the inclusion of calcium phosphate bioceramics, and bone related applications. The selected studies were meticulously analyzed for key aspects such as resin formulations, manufacturing parameters, rheological properties, post-printing processes, mechanical properties, and, finally, biological performances. A particular emphasis was placed on comparing the mechanical performance of the scaffolds based on their composition and architecture, offering a unique categorization and comparative analysis.Results: This study offers a deep insight of the latest studies, by providing details with a novel approach to understand the field's progress. Two primary fabrication routes were determined, each leading to distinct structural and functional properties. Namely, over 75 % of the studies focused on creating full ceramic bodies, while the remaining 25 % explored composite structures. Additionally, this study discusses the latest strategies performed by researches to enhance both the mechanical and biological performance of bone scaffolds. These include advanced innovative scaffold compositions, post-processing techniques, and architectural designs.

## 1 Introduction

Bone is a highly hierarchical and metabolically active tissue that provides support and protection to vital organs, stores essential ions, and plays a critical role in maintaining bone homeostasis. Despite its regenerative capacity, bone healing can be impaired by systemic factors like age, diabetes or malnutrition, and local factors like poor blood supply, infection, or the presence of large defects caused by trauma or tumor resection. In such scenarios, bone grafting is a common procedure to restore bone function and structure. Worldwide, an estimated 2.2 million bone grafting procedures are performed annually, and are expected to grow by 13 % each year [1]. While autologous bone grafts, so-called autografts, remain the gold standard, the use of synthetic bone grafts for orthopedic interventions strongly surged from 11.8 % in 2008 to 23.9 % in 2018 [2]. In the last ten years, the demand for synthetic-based biomaterials increased by 134 % compared to autografts, which varied by 74 %, and allografts, whose demand decreased by 14 % [2].

Bone tissue engineering addresses large bone defects by integrating biomaterials, cells, and signaling molecules with scaffolds. These temporary structures support bone remodeling while minimizing complications [3]. They enable defect filling with personalized shapes, providing mechanical support, and guiding new tissue growth [4]. Designed to mimic the extracellular matrix, scaffolds facilitate cell adhesion, proliferation, differentiation, and the exchange of nutrients and waste while gradually degrading and being replaced by newly formed bone [5,6].

Synthetic bone grafts have been extensively developed and have achieved clinical relevance, particularly calcium phosphate (CaP) bioceramics. CaPs closely resemble the mineral phase of natural bone in terms of composition and have been shown to be biocompatible, bioactive and osteoconductive. Moreover, in certain configurations, they have demonstrated osteoinductive properties, promoting the differentiation of cells into the bone lineage [7]. One of the main motivations fostering CaP research is the key role of Ca mineralization in endochondral ossification during early fetal development, leading to bone tissue formation. On the other hand, the degradation of certain synthetic CaP-based biomaterials can be integrated into the physiological process of bone remodeling. Finally, the presence of calcium (Ca^2+^) and phosphorous (P_i_), at specific concentrations, can enhance the proliferation and differentiation of osteoprogenitor cells [8]. Despite their success, their application is largely limited to non-load-bearing scenarios due to their inherent brittleness. To address these structural limitations, calcium phosphates have been combined with biocompatible polymers, providing enhanced elasticity and toughness [9].

Despite the close resemblance to the natural bone mineral phase, CaP geometrical design has been a limiting factor in their overall structural integrity and implementation. Often produced in standardized shapes or blocks, CaPs pre-formed designs fail to adapt to patient-specific bone defects, requiring manual adjustments and tailoring by surgeons, which can further compromise material properties and the clinical outcome. Traditional methods such as particulate leaching [10,11], emulsions, freeze-drying, foaming [12], or the use of templates enable the fabrication of porous scaffolds with high interconnectivity and porosity, similar to bone natural structure [13]. However, while offering simplicity and versatility, these techniques yield poor reproducibility and low patient-specificity [14]. The emergence of cutting edge technologies such as additive manufacturing (AM) has enabled the creation of complex customized scaffolds, with interconnected macroporous architectures that mimic the structure and hierarchy of natural bone [4]. Additive manufacturing (AM), and more precisely 3D printing offers a promising approach to personalized medicine. It allows fabricating patient-specific scaffolds with complex geometries, with high reproducibility and accuracy, not only of the external shape but also of the internal architecture [6].

The ASTM F2792-12a standard identifies seven distinct 3D printing technologies: binder jetting, directed energy deposition, material extrusion, material jetting, powder bed fusion, sheet lamination, and vat photopolymerization. Regarding ceramic 3D printing, these seven technologies can be categorized into four groups: i) Powder bed-based AM, where a roller or scraper system spreads a layer of powder or slurry onto the printing substrate, and a device selectively binds the desired regions; ii) Dispensing-based AM, which uses a dispensing device controlled by a 3D positioning system to progressively deposit material layer by layer, forming a 3D structure; iii) Lamination-based AM, where sheets of material are layered on the printing area, and a cutting device defines the regions of interest; and iv) Vat photopolymerization AM, based on the polymerization of liquid polymer precursors or suspensions with the incorporation of colloidal ceramic particles in the case of ceramic printing [15]. Each of these techniques has its advantages and limitations for the printing of calcium phosphate-based scaffolds, in terms of resolution and material selection.

Dispensing-based AM techniques, such as fused deposition modelling (FDM) and direct ink writing (DIW) are very versatile in terms of materials, allowing to print different polymers loaded with calcium phosphate particles, which can be subsequently sintered or not, resulting in full ceramic or composite scaffolds. Ceramic-FDM works by depositing a composite filament from a nozzle by reducing its viscosity through melting. It has been widely adopted with CaPs to create suitable and robust composites by incorporating biocompatible and bioresorbable thermoplastic polymers such as polycaprolactone (PCL) [16,17] or polylactic acid (PLA) [[18], [19], [20]] without requiring solvents. This approach has enabled the fabrication of dimensionally accurate and mechanically enhanced scaffolds [17]. However, FDM has limitations in terms of ceramic loading, typically achieving optimum loadings ranging from 5 to 15 wt%, with some exceptions incorporating up to 30 to 50 wt% [20]. These values are significantly lower than the mineral content of natural bone, which is around 60–70 wt% [21]. In contrast, DIW, which entails the extrusion of a paste, operates at low temperatures (e.g., room temperature), offering new possibilities for bone scaffold manufacturing [9]. For instance, it can be made compatible with cell printing, which is not possible when using FDM [22]. Additionally, DIW supports higher ceramic loading, enabling the extrusion of highly loaded ceramic pastes that, after post-processing, result in full-ceramic 3D printed porous bioceramic scaffolds [[23], [24], [25]]. To overcome the brittleness of full-ceramic scaffolds, biocompatible polymers such as poly-lactic-co-glycolic acid (PLGA) [26], and PCL [9,27] have also been incorporated into the inks for DIW, resulting in composite scaffolds with enhanced toughness. This approach, compared to FDM, allows increasing the ceramic content, reaching up to 70 wt% and showing improved mechanical and biological properties. However, material extrusion AM requires ceramic suspensions with appropriate viscosity and shear-thinning behavior [28]. To ensure smooth and steady extrusion, the nozzle size is generally large in order to meet a feasible extruding force, ranging from hundreds to thousands of newtons for high-load suspensions. This creates a trade-off between printing accuracy and printability [29]. As a result, printing resolutions are typically above 100 μm [30], limiting the possibility of creating complex structures with high precision that could mimic the architecture of natural bone, even when support structures are used [31].

Higher resolution AM methods such as powder bed-based techniques are also commonly used for scaffold fabrication. Powder bed fusion (PBF) uses a high-energy laser or electron beam to selectively melt (Selective Laser Melting; SLM) or sinter (Selective Laser Sintering; SLS) the spread powder, bonding the particles into a dense structure. In contrast, Binder Jetting (BJ) uses a dispensing nozzle to selectively deposit a liquid binder onto the powder, bonding the particles together in specific areas. These AM techniques allow the production of complex geometries and architectures with the great advantage of not needing support structures in the printing process. However, each technique has its trade-offs when fabricating CaP scaffolds. SLS-based strategies pose a number of challenges in both processing and post-processing. CaPs exhibit very high melting temperatures, making them hard to process, as they require high-power lasers capable of heating ceramic powders to densify particles into stacked 2D layers [32]. While PBF provides high mechanical properties, high precision, and no need for binders, the melting/sintering process may cause phase transformations, temperature gradients and internal residual stresses and cracks [33]. One possible alternative is the incorporation of lower-melting-point thermoplastic polymers such as PCL, poly-L-lactic acid (PLLA), polyether ether ketone (PEEK), or polyamide (PA) to obtain composite structures [34]. However, achieving high ceramic loadings is challenging, as it requires increased laser energy, creating a compromise between ceramic loading, printability, and energy consumption. As a result, composite scaffolds fabricated with PBF normally contain 5-50 wt% ceramic content [34]. In opposition, BJ operates at low processing temperature, resulting in lower probability of cracks and no residual stresses. This technique also enables higher ceramic loadings, reaching >70 wt% [35]. Nonetheless, BJ-printed scaffolds experience lower mechanical strength, and lower resolution due to potential binder infiltration, affecting the scaffold's final porosity. Overall, powder bed-based AM techniques are promising approaches for creating complex scaffolds with high precision, achieving layer thicknesses of about 30–200 μm for SLS/SLM and 50–200 μm for BJ [32,36]. However, they are often limited in terms of resulting composition, as a sintering process is needed to remove the binder and fuse the ceramic particles together. Additionally, the large size and high cost of these machines are limiting factors when adapting their integration into clinical or hospital settings.

In this context, Vat Photopolymerization (VP) appears as an innovative technique that has gained increasing attention in medical applications, especially due to its high precision, achieving resolutions of a few tens of micrometers. Compared to other AM techniques, VP offers significant advantages in addressing some of the previously mentioned challenges. Specifically, calcium phosphate particles are mixed into liquid resins to form ceramic suspensions, which are processed in vats where light selectively solidifies layers at low temperatures. Unlike FDM, which requires the material to pass through heated nozzles, VP enables the use of resins heavily loaded with calcium phosphate particles (10–70 wt%) [[37], [38], [39]]. While VP does present challenges related to light-material interactions, it offers greater design capability than material extrusion techniques, enabling the fabrication of complex geometries similar to the architecture of natural bone. Additionally, although challenging, the rheological demands are less stringent than in material extrusion techniques. Furthermore, compared to SLS/SLM, VP does not operate with highly powered lasers and does not require high temperatures, making this technique a more affordable variant that could potentially be integrated into clinical and hospital settings. The risk of defects such as cracks, phase transformations, and dimensional distortions during printing is reduced. However, VP requires extensive post-processing, including cleaning, which can be difficult with tight and low-porous designs, often leading to pore occlusions and residual uncured resin inside the structure. Moreover, common resins often contain toxic components, requiring a binder removal step followed by sintering, resulting in mechanically weak full-ceramic parts. Recent advances in the formulation of biocompatible resins have expanded the potential for obtaining composite structures, a promising approach to overcome post-processing limitations, and enhance mechanical and biological performances.

This review aims to summarize the progress and future perspectives of VP manufacturing of calcium phosphate-based scaffolds for bone tissue engineering, including: (1) the principles of CaP VP printing and the slurry requirements; (2) the composition and processing strategies of VP-printed CaP-scaffolds; (3) the effect of the material and processing routes on the mechanical and biological response; and (4) the strategies that are being explored to improve the mechanical and biological performance of the scaffolds. Although ceramic VP has been used with various bioceramics and bioactive glasses, this study focuses specifically on calcium phosphates as key materials in bone grafting due to their close resemblance to the mineral phase of bone. Furthermore, the growing interest toward incorporating these materials into advanced VP approaches cannot be overlooked, as it holds significant relevance for an increasingly engaged research community (Fig. S1 in Supplementary information). This review was conducted through a bibliographic search of scientific articles using three databases, Scopus, Web of Science, and PubMed. Other sources of information such as patents or clinical study reports may contain valuable but more fragmented and less accessible information, and were considered outside the scope of this study. Similarly, this review is specifically focused on calcium phosphate for bone regeneration and does not address other types of ceramics that may serve different applications with distinct structural and functional requirements. The keywords and search strategy are detailed in the Supplementary information.

## 2 Vat photopolymerization (VP) techniques

VP manufacturing involves selectively curing a photosensitive material, usually polymers, using a light source, typically in the ultraviolet range. During this process, a tank containing the photosensitive resin is exposed to light, forming the layers of the final geometry which is attached to a building platform. This exposure can either be from the bottom up through a transparent film into the tank containing the resin (bottom-up approach), or from the top down to the tank containing the resin (top-down approach). The light travels from the light source and penetrates the photosensitive resin, colliding with the building platform. The material in between solidifies and attaches to the moving building platform, also known as the build plate, becoming the first layer. The build plate retraces back and the following layers solidify and adhere to one another progressively, creating the final piece layer by layer.

**Fig. 1:**
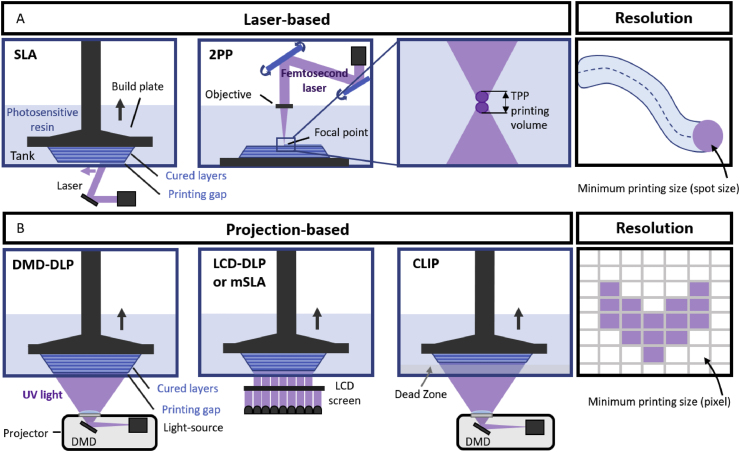
Schematic representation of Vat Photopolymerization techniques, categorized as: (A) laser-based techniques, such as Stereolithography (SLA) and Two-photon Polymerization (2PP); and (B) projection-based techniques, such as Digital Light Processing (DLP), Liquid Crystal Display-based masking projection (LCD-DLP), or masked stereolithography apparatus (mSLA), and Continuous Liquid Interface Production (CLIP).

VP techniques can be categorized depending on the employed light sources during photocuring. Basically, laser-based (Fig. 1A) and projection-based sources can be used (Fig. 1B). Laser-based techniques create a linear pattern for each layer. Common examples include Stereolithography (SLA), which uses a laser beam to cure the resin layer by layer linearly (either bottom-up or top-down), and Two-Photon Polymerization (2PP), which utilizes femtosecond lasers to cure the resin at a precise focal point. Projection-based techniques irradiate an entire pattern directly onto the resin, curing it all at once. Typical examples include Digital Light Processing (DLP), which uses a digital micromirror device (DMD) to project the image; Continuous Liquid Interface Production (CLIP), which creates a dead zone hampering the curing of the resin (enabling continuous production); and LCD-DLP 3D printing, also known as Masked Stereolithography (mSLA), which is similar to DLP but uses an LCD panel to mask the UV light source [40].

The most popular techniques for VP printing of calcium phosphate-based materials are DLP, accounting for over 75 % of the reviewed publications, and SLA, used in around 25 % of the publications.

### 2.1 Stereolithography (SLA)

SLA is considered one of the earliest VP techniques, first developed in 1986 by Charles Hull [29]. SLA involves using a laser that scans a photosensitive liquid resin leaving behind a solidified line of crosslinked polymer. The photosensitive resin is placed in a vat and the laser scans linearly, selectively irradiating specific areas which become a solidified layer. This process is repeated layer by layer, solidifying and adhering consecutive layers to each other, and resulting in the final print. Typical commercial printers use tens of microwatts lasers (such as a 30 mW [41]), and can be set to hundreds of microwatts (100 mW [42]), and even higher, up to 300 W [43] when employing custom-made SLA printers. The laser beam penetrates solidifying tens of μm high in the z-axis linear patterns. In the x-y plane, the resolution depends on the laser beam-spot size, as represented in Fig. 1A, which is easily adapted to create complex and detailed geometries. However, SLA has some limitations in terms of printing time, as the laser scan results in a slow printing speed.

### 2.2 Digital Light Processing (DLP)

DLP is a projection-based technique which involves projecting a pattern of light at once onto a photosensitive liquid resin using LED arrays projected towards a Digital Micromirror Device (DMD) or a liquid crystal display (LCD). First, the build plate is immersed into a ceramic-loaded photosensitive resin (or slurry). The movable build plate descends beneath the liquid surface leaving a programmed gap (layer height) between the build plate and the bottom of the transparent vat. Then, the light source, typically a lamp or LED array, irradiates onto the DMD or the LCD, which directs it to the bottom of the tank in the designed pattern. In the case of DMD, multiple micrometric mirrors selectively move to either reflect light towards the vat, or hinder the exposition at that specific location. This way, each micromirror acts as a pixel that solidifies a quantified volume or voxel. Once the slurry solidifies, the platform separates from the bottom of the tank enabling the slurry to flow back underneath. Then, the build plate with the first layer attached to its surface descends again leaving the latter layer height between the hardened layer and the bottom of the tank. The process is repeated until the scaffold is formed layer by layer [44]. Since it prints the entire layer simultaneously, DLP is faster than SLA. On the other hand, LCD screens are controlled by a computer, selectively blocking the light and exposing only the transmission areas that will form the layer. One key advantage of this technology compared to DMD is its relatively more affordable cost, making it the most common commercially available option. However, LCD has some limitations in terms of printing size, which is restricted by the LCD panel, and less lifespan when compared to a projector with DMD [29].

The printing resolution in the z-axis, ranging from 20 to 100 μm, is associated with the layer height and is highly dependent on the stepper motor or linear actuators controlling Z-axis platforms. The most typical layer heights are 25–50 μm, although lower values, down to 20 μm, have been achieved [45,46]. The resolution in the x-y axes, on the other hand, can significantly vary between DMD or LCD due to their differences in their light modulation mechanics and optics. Typically, the resolution is limited to the pixel size that the projection system creates on the resin, resulting in a solidified voxel as the minimum printing volume, represented in (Fig. 1B). While DMD x-y resolutions typically range from 50 to 100 μm, LCD systems can achieve 50–75 μm; however, DMDs provide higher pixel size consistency and uniformity compared to LCD systems where light intensity can vary across the build area and reduce precision.

## 3 Photocurable resins for Vat photopolymerization of calcium phosphate-based materials

VP of calcium phosphate-based scaffolds has garnered much attention in the field of bone regeneration. Calcium phosphate-loaded photosensitive resins consist of a colloidal suspension of calcium phosphate particles in a liquid photosensitive polymeric resin. Under irradiation, a continuous cross-linked polymeric network containing the calcium phosphate particles is obtained [3,47,48]. The polymeric resin contains polymeric monomers/oligomers, light-sensitive initiator molecules (photoinitiators), and small concentrations of other additives. The selection of the appropriate components and their concentrations plays a crucial role in determining the polymerization resolution, printing time, and accuracy, thereby tailoring the functional properties of the 3D printed structure [30]. These components are typically mixed before printing, using either a planetary ball milling or centrifugal mixers. The elements in the slurry must be carefully selected to ensure high precision, and will be described in the following sections.

### 3.1 Photocurable polymeric resins

#### 3.1.1 Calcium phosphate-loaded photocurable resins

The central components of the resin are the photocurable prepolymers (monomers or oligomers), which act as the building blocks of the final solidified part. These molecules contain crosslinking functional groups that interact with each other, forming a network. The most commonly used prepolymers are synthetic acrylated prepolymers like poly(ethylene glycol) diacrylate (PEGDA) [37,[49], [50], [51], [52], [53]], 1,6-hexanediol diacrylate (HDDA) [[54], [55], [56], [57], [58], [59]], trimethylolpropane triacrylate (TPMTA) [55,[60], [61], [62], [63], [64]], poly(trimethylene carbonate)-methacrylate (PTMC-MA) [[65], [66], [67], [68], [69]], among others. These synthetic polymers provide adequate, mechanically robust structures and are non-cytotoxic, although they are inert in terms of cell-material interactions and often face poor cell adhesion. Natural polymers such as gelatin, collagen, silk-fibroin, or alginate in their acrylated forms (methacrylated gelatin (GelMA) [[70], [71], [72], [73]], methacrylated collagen (ColMA), methacrylated silk-fibroin (SilMA) [74], methacrylated alginate (AlgMA) [75]) have been more recently explored in the field of VP printing for medical applications. Bone-derived decellularized extracellular matrix methacrylate (bdECM-MA), rich in collagen-, glycosaminoglycans- (GAGs), and other bone-specific ECM proteins, is another promising material recently used in VP bioprinting [76]. These materials closely resemble the polymeric composition of natural bone, potentially exhibiting better specificity in cell-material interactions. Another prepolymer that has been recently proposed for the preparation of CaP-containing resins is CSMA-2 ((3R, 3aR, 6S, 6aR)-hexahydrofuro [3,2-b] furan-3,6-diyl)bis(oxy)) bis(ethane-2,1-diyl))bis(oxy))bis(carbonyl))bis(azan ediyl))bis(3,3,5 trimethylcyclohexane-5,1-diyl))bis (azanediyl))bis(carbonyl))bis(oxy))bis(ethane-2,1-diyl) bis(2-methylacrylate)). This novel synthetic bio-based prepolymer has shown excellent printability, good mechanical properties and biocompatibility *in vitro* and *in vivo* [39,77], with great osteogenic and angiogenic potential [78].

#### 3.1.2 Photoinitiators

Under light irradiation, the prepolymers start to crosslink to form the solidified network due to the action of photoinitiators. These are molecules that create reactive species in the form of free radicals or cations/anions when exposed to radiation, initiating the polymerization reaction by interacting with the monomers/oligomers. Thereby, the crosslinking mechanisms are categorized as either radical polymerization or cationic polymerization [79]. Some of the most used monomers/oligomers and photoinitiators are displayed in Table 1, detailing their chemical composition and optimal operating wavelength.

**Table 1:** Common monomers/oligomers and initiator molecules used in the formulation of photocurable resins. Typical (meth)acrylated monomers/oligomers are selected for their double bonds, which react with each other to form covalent bonds, thus enabling chemical crosslinking (TMPTA molecule figure taken from Ref. [69], and CSMA-2 from Ref. [39]). The initiation and propagation of this reaction are driven by photoinitiator molecules, which convert photolytic energy into reactive species that initiate the polymerization process (photoinitiator molecule figures taken from Ref. [79])*.*

Monomer/Oligomer	Molecule	Initiator	Molecule
Poly(ethylene glycol) diacrylate (PEGDA)		Diphenyl(2,4,6-trimethylbenzoyl) phosphine oxide (TPO)	
		*λ*_peak_ 380 nm	
1,6-hexanediol diacrylate (HDDA)		Ethyl phenyl(2,4,6-trimethylbenzoyl) phosphinate (TPO-L)	
		*λ*_peak_ 379 nm	
Trimethylolpropane triacrylate (TMPTA)		Lithium phenyl-2,4,6 trimethyl-benzoyl phosphinate (LAP)	
		*λ*_peak_ 450 nm	
Poly(trimethylene carbonate)-methacrylate (PTMC-MA)		Phenyl bis (2,4,6-trimethylbenzoyl) phosphine oxide (BAPO)	
		*λ*_peak_ 370 nm	
CSMA-2			

The most commonly used mechanism in VP printing is radical photopolymerization. The mechanism is based in the reaction of monomer/oligomer functional groups with free radicals, forming covalent bonds between prepolymers and resulting in crosslinked chains [80]. The process follows three steps: (i) radical generation: under light irradiation photoinitiator molecules react with photons of a specific energy (wavelength), and are responsible of converting this energy into reactive species; (ii) initiation: these species react with prepolymer molecules initiating the polymer chain reaction; and (iii) propagation: polymerization process [30,79,81]. Free radical initiators themselves can be categorized as Norrish-type I (also called α-cleavage), and Norrish-type II. Type I free radical initiators are the most used photoinitiators in VP printing with calcium phosphates. They undergo photo-cleavage resulting in two radical species, both being capable of initiating the polymerization. The wavelength and intensity of light needed to trigger cleavage vary based on the chemical structures of the photoinitiators [79]. Common type I initiators include phosphine oxide-containing molecules, such as diphenyl(2,4,6-trimethyl benzoyl)phosphine oxide (TPO; *λ*_peak_ ∼ 380 nm), ethyl phenyl(2,4,6-trimethylbenzoyl)phosphinate (TPO-L; *λ*_peak_ ∼ 379 nm), and phenylbis(2,4,6-trimethyl-benzoyl)-phosphineoxide (BAPO; *λ*_peak_ ∼ 370 nm) (Table 2). In fact, TPO and BAPO, and their commercial branding names (Irgacure®, Omnirad®) are widely used because of their efficiency. Other type I initiators used in VP printing include lithium phenyl-2,4,6 trimethyl-benzoyl phosphinate (LAP; *λ*_peak_ ∼ 405 nm) [82]. Free-radical type II initiators generate radicals in the presence of a co-initiator, typically hydrogen donating compounds, such as amines, thiols or alcohols. The most commonly used type II initiators are camphorquinones (CQ, *λ*_peak_ ∼ 480 nm) and thioxanthones [79], such as 2-isopropyl-9h-thioxanthen- 9-one (ITX) [37]. However, compared to type I initiators, type II initiators such as CQ have low photoreactivity, often addressed with the addition of tertiary amines as electron/proton donors or reducing agents [83].

**Table 2:** Resin formulations and rheological properties, printing parameters and post-printing processes for vat photopolymerization printing of calcium phosphate full-ceramic scaffolds (D: debinding; CD: chemical debinding; S: sintering; d: dose, I: intensity, P: power, LH: layer height, *λ*: curing wavelength).

CaP	Loading	Slurry composition	VP printing	Slurry's viscosity	Post-process	Ref.
HA	_	Commercial slurry: LithaBone HA 480E (Lithoz GmbH, Vienna, Austria)	DLP (Cerafab7500)	_	D: up to 800 °C, or CD: LithaSol 30	[123,124,126,130]
			LH: 25 μm		S: 1275–1300 °C for 2 h	
			*λ*: 460 nm			
			d: 150 mJ/cm^2^			
	48 wt%	Commercial slurry: HAPM100T01 (CERHUM, Belgium)	DLP (Propmaker V6000)	_	S: 1170 °C, 1270 °C for 5–90 h	[128,129]
			LH: 50 μm			
			*λ*: 365 nm			
	45 wt%	Commercial slurry: Shanghai Guangyi Chemical Co., Ltd., China	DLP	<12 Pa s	D: 500 °C for 4 h	[101]
		Dispersant: SPA	LH: 100 μm		S: 1400 °C for 1.5 h	
			*λ*: 405 nm			
			I: 11 mW/cm^2^			
	Protected		SLA (3D Ceram)	_	D: 240-460-800 °C	[99,131]
					S: 1050 °C	
	40 vol%	Monomer: PEGDA (Mn = 250)	DLP (custom)	0.18 Pa s	S: at 1300 °C for 2 h	[51]
		Initiator: 2 wt% PPO (BAPO)	LH: 100 μm			
		Dispersant: 3 wt% Triton X-100	*λ*: 380–420 nm			
			I: 0.5 mW/cm^2^			
	50 vol%	Monomers: HDDA + HEMA + TMPTA (6:3:1)	DLP (AutoCera)	10–15 Pa s at 50 Hz	S: 1250 °C	[60]
		Initiator: TPO	LH: 25 μm			
		Dispersant: 2 wt% Solsperse 17000	*λ*: 405 nm			
			I: 8 mW/cm^2^			
	50 wt%	Commercial resin: Shanghai, China	DLP (custom)	_	S: 1500 °C for 3 h	[132]
	35 vol%	Commercial resin: Rigid resin (XYZ Printing inc. Taiwan) + HDDA (∼3:5)	SLA (Novel 1.0, XYZ)	3.6–6 Pa s	S: 1250 °C	[102]
		Dispersant: 1.5 wt% BYK 180	LH: 50 μm			
			*λ*: 405 nm			
	30 vol%	Monomer: HDDA	DLP (Photon, Anycubic)	_	S: 1250 °C for 2 h	[59]
		Initiator: BAPO (Omnirad 819)	LH: 50 μm			
		Dispersant: 0.15 wt% OA				
	55 wt%	Monomer: HDDA	DLP	380 mPa s at 52 Hz	S: 1250 °C for 3 h	[106]
		Initiator: TPO	LH: 30 μm			
		Dispersant: 3 wt% BYK (not specified)	*λ*: 405 nm			
			I: 15 mW/cm^2^			
	50 vol%/68 wt%	Monomers: OPPEA + HDDA (mass ratio 1:1)	SLA (Ceramaker 100)	1.25 Pa s at 100 Hz	S: 1100, 1200, 1300 °C for 2 h	[41]
		Initiator: TPO	LH: 50 μm			
		Dispersant: 0.2 wt% S18	*λ*: 405 nm			
			P: 30 mW			
	30 vol%	Monomers: HDDA + TMPTA (4:1)	DLP (AutoCera)	_	S: 1250 °C for 2 h	[104]
		Initiator: TPO	LH: 50 μm			
		Dispersant: Solsperse KOS163	*λ*: 405 nm			
			I: 0.9 mW/cm^2^			
	45 vol%	Monomers: HDDA + HEMA + TMPTA (6.3:1)	DLP (AutoCera)	_	S: 1250 °C for 2 h	[64]
		Initiator: 1.5 wt% TPO (regarding resin)	LH: 25 μm			
		Dispersant: 2 wt% Solsperse 17000 (regarding powder)	*λ*: 405 nm			
			I: 8 mW/cm^2^			
	60 wt%	Monomer: Not specified	DLP (Admaflex 130)	_	S: 1150 °C for 2 h	[107]
		Initiator: TPO				
		Dispersant: BYK 2155				
	46 vol%	Commercial: LithaBone HA400 (Lithoz GmbH)	DLP (CeraFab7500)	_	S: 1300 °C	[125]
			LH: 25 μm			
			I: 56 mW/cm^2^			
	50 vol%	Commercial: Dentifix-3D (FunToDo®)	mSLA (Phrozen shuffle)	0.065 Pa s at 100 Hz, <5 Pa s at 1 Hz, TSI<2	S: 1250 °C for 2 h	[117]
		Diluent: 35 vol% PEG-200	LH: 100 μm			
			*λ*:405 nm			
			P: 50.000 mW			
	38 vol%	Commercial: 62 vol% LithaBone, not specified (Lithoz GmbH)	DLP (CeraFab7500)	_	CD: LithasSol 80	[133]
			LH: 25 μm		S: 900–1300 °C	
	40 vol%	Monomer: Acrylate oligomers (ACMO)	LCD-DLP (Phrozen)	1,2 Pa s - 1.8 Pa s at 10 Hz	S: 1250 °C for 9 h^a^	[115]
		Absorber: Light Stabilizer 292	LH: 50 μm			
		Dispersant: SPA	*λ*: 460 nm			
	55 wt%	Commercial: Sylgard 184 silicone elastomer kit (3DCeram Sinto, France)	SLA (3DCeram Sinto)	_	S: 1280 °C for 1 h	[134]
			LH: 100 μm			
			P: 48 mW			
	0-60 wt%	Monomer: Cyracures UVR-6105	SLA (custom)	<3 Pa s at 100 Hz	_	[43]
		Initiator: Cyracures UVI-6976	LH: 100 μm			
			*λ*: 370 nm			
			P: 300 W			
	27 wt%	Monomer: methacrylate-based (not specified)	DLP (custom)	_	S: 1300 °C for 2 h	[135]
			LH: 50 μm			
			I: 28 mW/cm^2^			
	50 wt%	Polyfunctional acrylic resins (not specified)	SLA (Prodways V6000)	_	S: 1125 °C, for 5 h	[136]
			LH: 50 μm			
	50-56 vol%	Monomer: MBAM	SLA (SPS450B)	<3 Pa s at 30 Hz	S: 1080 °C	[96]
		Initiator: photocure-1173	LH: 100 μm			
		Dispersant: ammonium polyacrylate	P: 300 mW, d: 20.3 mJ/cm^2^			
	10-45 wt%	Commercial: DSM's Somos (ABSlike)	DLP (LAYING II 1510P)	_	D: 500 °C	[137]
			LH: 50 μm		S: 1250 °C	
	5, 10, 20 wt%	Commercial resin: FormLabs Ceramic RS-F2-CEWH-01	SLA (FormLabs Form2)	3.4–4.1 Pa s at 12 rpm	D: up to 300 °C	[138]
			*λ*: 405 nm		S: 1270 °C	
			LH: 100 μm			
	60 wt%	Monomers: PUA + PEGDA (Mn 400) (3:1 ratio)	DLP (Admaflec 130 plus)	_	S: 1150 °C for 2 h	[139,140]
		Coating: GelMA (20 wt%) + Icariin (for drug release)				
		Initiator: 2 % TPO-L				
		Dispersant: BYK 2155				
	50 wt%	Monomers: HDDA	LCD-DLP (Sonic 4K, Phrozen)	<150 mPa s	D: up to 600 °C	[141]
		Initiator: 3 wt% TPO	LH: 50 μm		S: 1200 °C for 2 h	
		Dispersant: BYK 111				
	50 wt%	Monomers: UA + PEGDA (Mn400) (3:1)	DLP	_	S: 1150 °C for 2h	[142]
		Initiator: TPO	LH: 50 μm			
		Dispersant: BYK 2155				
	45 wt%	Monomer: HDDA	DLP (Autocera-R)	1 Pa s at 50 Hz	S: 1200 °C	[95]
		Initiator: 1.5 wt% BAPO (regarding resin)	*λ*: 405 nm			
		Dispersant: 5 wt% Solsperse 41000 (regarding ceramic)				
		Absorbers: 0.2 wt% MEHQ (regarding resin)	I: 5 mW/cm^2^			
		Porogen: ethylene glycol	LH: 50 μm			
	50-70 wt%	Monomers: UA + PEGDA-400 (3:1)	DLP (Admaflec 130+)	0.3–2 Pa s	S: 1050-1150-1250 °C for 2h	[49,50]
		Initiator: TPO	LH: 65 μm			
		Dispersant: BYK 2155				
HA + Al_2_O_3_	20-80 wt%	Monomers: Trimethylolpropane formal acrylate + PEGDA	DLP (CeraStation 160)	_	S: 1400 °C	[143]
		Initiator: BAPO	*λ*: 405 nm			
		Dispersant: SP710	I: 127.23 mW/cm^2^			
			LH: 50 μm			
HA + ZrO_2_	45-70 wt%	Commercial: (Shanghai Guangyi Chemical Co., Ltd.) and (Shanghai Prismlab Co., Ltd.) respectively	DLP (custom, SU-100A)	_	D: 500 °C for 4 h	[45,46]
		Dispersant: 2 wt% SPA	LH: 20 μm		S: 1400 °C for 1.5h	
			*λ*: 405 nm			
			I: 10 mW/cm^2^			
	70 wt%	Monomer: PEGDA (Mn 600)	DLP (custom)	_	S: 1200, 1300, 1400 °C	[37]
		Initiator: TPO	*λ*: 405 nm			
		Absorber: Sudan red	I: 0.9 mW/cm^2^			
		Dispersant: KH-570				
	_	Monomers: PEGDA + Hydroxyethyl methacrylate phosphate	DLP	_	S: 1200 °C	[144]
		Initiator: 0.5 % TPO	*λ*: 405 nm			
		Dispersant: KH-570 + ACMO	LH: 40 μm			
		Absorber:3·10^−5^ wt% Sudan				
	10-60 wt%	Monomers: 45 % HDDA + 35 % ACMO + 15 % TMPTA + 5 % hyperbranched polyester acrylate	DLP (custom)	10–100 mPa s at 100 Hz	D: up to 600 °C	[63]
		Initiator: 0.5 % BAPO (Omnirad® 819)	*λ*: 405 nm		S: 1100–1250 °C	
		Dispersant: KH-570 + OA + Castor oil				
HA + AK (9:1)	40 vol%	Monomers: 60 wt% HDDA + TPGDA (7:3)	DLP (Autocera-M)	_	D: 600 °C for 3 h	[[56], [57], [58]]
		Initiator: 0.5 wt% TPO	LH: 50 μm		S: 1000–1250 °C for 2h	
		Dispersant: 4 wt% Solsperse 41000	d: 8 mJ/cm^2^			
	40 vol% + nano-Fe_3_O_4_	Monomers: HDDA + TPGDA (7:3)	DLP (Autocera-M)	_	S: 1100 °C for 2 h	[105]
		Initiator: 0.5 wt% TPO (regarding resin)				
		Dispersant: 4 wt% Solsperse 41000				
Si-HA	55 vol%	Monomer: amine modified polyester acrylate	DLP (PμSLA) (Xianlin 3D)	2 Pa s at 150 Hz (<5 Pa s)	S: 1160–1200 °C for 2 h	[88]
		Initiator: EDMD	LH: 250 μm			
		Dispersant: phosphate ester	*λ*: 365 nm			
HA + SiO_2_	35 wt%	Monomer: 60 wt% SR454NS acrylic resin	DLP	<4 Pa s	S: 1200 °C for 3 h	[120]
		Initiator: 0.5 wt% Ethyl 4-(Dimethylamino) benzoate	LH: 25 μm			
		Dispersant: 2 wt% TAEA				
HA + Sr^2+^ + Mg^2+^ + Zn^2+^	38 vol%	Commercial resin: 62 vol% Lithoz GmbH	DLP (CeraFab 7500)	_	D: up to 500–600 °C	[133]
			LH: 25 μm		S: 900, 1000, 1100, 1200. 1300 °C	
HA + CaSiO_2_ + SrPO_4_ + CaSO_4_	30 wt%	Monomers: acrylic, acrylate monomer	DLP (custom)	_	D: up to 715 °C for 3 h	[116]
		Dispersant: SPA	LH: 40 μm		S: 1300 °C for 2 h	
			*λ*: 405 nm			
HA + BR	55 wt%	Monomers: HDDA + TPGDA (7:3)	DLP	0.5–1 Pa s at 30 Hz	D: up to 600 °C	[145]
		Initiator: 0.5 wt% TPO			S: 1300 °C for 2 h	
		Dispersant: 4 wt% BYK 111				
HA + BG (8:2)	50 wt%	Commercial rigid resin (Anycubic)	LCD-DLP	_	D: 600 °C at 5 °C/min for 2h	[146]
					S: 1300 °C at 5 °C/min	
HA + ZnO	60 wt% (95–15, 90–10)	Monomers: PUA + PEGDA (Mn 400) (3:1 ratio)	DLP (Admaflec 130 plus)	_	S: 1150 °C for 2 h	[147]
		Initiator: 2 % TPO-L				
		Dispersant: BYK 2155				
HA + BT (5:5, 3:7, 1:9)	55 wt%	Monomers: HDDA + TPGDA	DLP (Admaflex 130)	_	D: up to 600 °C at 1 °C/min	[148]
		Initiator: Initiator 819 (BAPO)	*λ*: 405 nm		S: 1300 °C for 3h at 3 °C/min	
		Absorber: HEMQ	LH: 40 μm			
		Dispersant: Triton X-100				
HA + BT (2:8)	45 vol%	Monomers: HDDA	DLP (Cerafab7500)	0.287 Pa s	D: up to 468 °C at 0.5–1 °C/min	[149]
		Initiator: Aladdin, Shanghai	*λ*: 453 nm		S: 1300 °C for 3h at 2 °C/min	
		Dispersant: 2 wt% KH-570	I: 87 mW/cm^2^			
			LH: 50 μm			
HA + BT (3/7) + ZnO	_	Monomers: 80 % PEGDA (Mn 600)	DLP	_	D: up to 600 °C	[150,151]
		Initiator: 2 % TPO-L	I: 2.5 mW/cm^2^		S: 1250 °C for 2 h	
		Dispersant: 15 % KH-570	LH: 20 μm			
		Absorber: 3 % Sudan red				
Whitlockite	75 wt%	Monomers: HDDA + TPGDA (7:3)	DLP (Anycubic D2)	0.5 Pa s at 30 Hz	D: up to 600 °C	[152]
		Initiator: 3 wt% TPO			S: 1000 °C for 2 h	
		Dispersant: 18.7 wt% BYK 111				
β-TCP	60 wt%	Monomers: PEGDA (Mw = 200) + β-CEA + HDDA (30: 5.2: 4.8) + HA-DA	DLP (custom)	_	S: 1150 °C	[153]
		Initiator: TPO	LH: 50 μm			
		Dispersant: KH-570	*λ*: 405 nm			
	60 wt%	Monomers: PEGDA (Mw = 200) + β-CEA + HDDA	DLP (custom)	2–3 Pa s (<3 Pa s at 30 Hz)	D: up to 536 °C	[97]
		Initiator: TPO	LH: 50 μm		S: 1150 °C for 4 h	
		Dispersant: 1 wt% KH-570	*λ*: 405 nm			
	70 wt%	Monomers: AM + MBAM	SLA (custom)	_	D: 80 °C/h to 660 °C, 115 °C/h to 700 °C	[38]
		Initiator: 0.02 wt% photocure-1173			S: 360 °C/h to 1150 °C for 1 h	
		Dispersant: SPMA				
	65 wt%	Commercial: CryoBeryl Software, France	SLA (CryoCeram)	_	S: 1050 °C for 3 h at 5 °C/min	[154]
			LH: 50 μm			
			*λ*: 350–400 nm			
			I: 5 mW/cm^2^			
	71 wt%	Monomers: 50 wt% HDDA + TGDA + HEMA + TTA + PEGDA	SLA (Admaflex 130)	3.5–4.4 Pa s (5–10 Pa s < 300 Hz)	S: 1100 °C for 3h	[155]
		Initiator: 1 wt% TPO	*λ*: 405 nm			
		Dispersant: 0.5 wt% Zelec *P*312				
		Diluent: 10 wt% PEG200				
	52 vol%	Monomers: HDDA + OPPEA	DLP (CeraRay CR-1)	5.76 Pa s at 100 Hz	D: up to 450 °C	[93]
		Initiator: 1 wt% TPO	LH: 100 μm		S: 1000 °C for 2 h	
		Dispersant: 2 wt% S18	*λ*: 405 nm			
	50 vol%	Commercial: HDDA, TMPTA, epoxy acrylic resin (Liangzhi chemical, Germany)	SLA (Ceramaker)	_	S: 1100 °C	[108,109]
		Dispersant: 2.5 wt% BYK 110	*λ*: 355 nm			
			P: 180 mW			
	30 wt%	Commercial: photosensitive resin (Anycubic Co, Shenzhen, China)	DLP (custom)	_	S: 1150 °C for 3 h	[119]
		Dispersants: 2 wt% PEG-600 + 3 wt% 1,5-pentanediol	LH: 50 μm			
	40-60 wt%	Commercial: acrylic resin (FTD Standard Blend 3D Printing resin, Fun To Do, Alkmaar, The Netherland)	DLP (3DLPrinter-HD 2.0)	_	S: 1200 °C for 2 h	[156,157]
		Dispersant: 0.1 wt% OA (regarding resin)	LH: 25 μm			
			*λ*: 400–500 nm			
			I: 10 mW/cm^2^			
	40 vol%	Commercial: acrylic resin (FTD Standard Blend 3D Printing resin, Fun To Do, Alkmaar, The Netherland)	DLP (3DLPrinter-HD 2.0)	1.9 Pa s at 10 Hz	S: RSA (rapid sintering) 5 min dwell, CSA (conventional sintering) 2 h dwell, SPS (vacuum) 5 min dwell at 1200, 1300, 1400, 1500 °C	[[158], [159], [160]]
		Diluent: 30 wt% Camphor	LH: 25–50 μm			
		Dispersant: 0.1 wt% OA (regarding resin)	*λ*: 385 nm			
			I: 31 mW/cm^2^			
	40 vol%	Commercial: resin (ELEGOO)	DLP (3DLPrinter-HD 2.0)	_	S: Conventional sintering (CS): 1200 °C for 3 h; 2-step sintering (2SS): 1250/1270/1290/1310 °C for 2 min + 1000 °C for 3 h	[121]
		Dispersant: 6 wt% Disperbyk 110	LH: 25 μm			
			*λ*: 405 nm			
			I: 13.2 mW/cm^2^			
	40 wt%	Commercial: 60 wt% FLGPCL02, Formlabs	SLA (SEPS) (custom)	_	S: 1250 °C for 3 h	[161]
			LH: 100 μm			
			*λ*: 405 nm			
	47 vol%	Commercial: Lithabone TCP 300 (Lithoz GmBH, Austria)	DLP (LCM) (Cerafab7500)	6–12 Pa s	D: up to 850 °C	[103]
			LH: 25 μm		S: 1200 °C	
			I: 101 mW/cm^2^			
	_	Commercial: LithaBone TCP 380 D	DLP (Cerafab7500)	_	D: 96 h	[122]
			LH: 25 μm		S: 1200 °C for 2 h	
	45 wt%	Commercial: resin (WANHAO Co.)	DLP (Autocera-M)	_	S: 1150 °C for 3 h	[162]
			LH: 50 μm			
	50 wt%	Commercial: resin (Suzhou Ding'an Technology Co.)	SLA (custom)	_	_	[163]
		Dispersant: SPA	I: 10 mW/cm^2^			
	40 vol%	Monomers: TMPTA + HDDA (1:1)	DLP (M-Jewelry U30)	<3 Pa s at 30 Hz	D: 600 °C for 2 h	[55,61,62]
		Initiator: BAPO (Omnirad® 819)	LH: 15–65 μm		S: 1100 °C for 2 h	
		Absorber: graphite	*λ*: 405 nm			
		Dispersant: KH-550	I: 2.19 mW/cm^2^			
	68 wt%	Commercial resin	SLA (CryoCeram)	_	D: 600 °C for 1 h at 1 °C/min	[164]
		Dispersant: Darvan C + B1001	LH: 50 μm		S: 1000, 1050, 1120 °C for 3 h at 5 °C/min	
	60 wt% _	Monomers: Aliphatic UA + HDDA (6:4)	DLP (Autocera-M)	_	S: 1100 °C	[165]
		Initiator: Hydroxy cyclohexyl phenyl ketone	LH: 25 μm			
		Dispersant: phosphoric acid ester	I: 10 mW/cm^2^			
		Monomer: PEGDA (Mw = 200)	DLP	_	D: up to 440 °C	[166]
		Initiator: TPO	LH: 50 μm		S: 1150 °C for 3 h	
			*λ*: 405 nm			
	43.1 vol%	Monomers: HDDA + TMPTA	SLA (Ceramaker 300)	_	S: 1200 °C	[167]
		Initiator: 3 % TPO	LH: 100 μm			
		Dispersant: 5 % JOS-110	*λ*: 365 nm			
			I: 52 mW/cm^2^			
	45 wt%	Commercial resin: 50 % SP700 photosensitive acrylic resin	DLP (Shaoxing)	_	S: 1160 °Cat 2 °C /min	[110]
		Dispersant: 5 % BYK 111	LH: 50 μm			
	50 wt%	Monomer: 49 wt% HDDA	SLA (Cerafab 8500)	_	D: 200 °C for 16 h	[168]
		Initiator: 1 wt% CQ	LH: 25 μm		S: 1200 °C for 4 h	
			*λ*: 406 nm			
			I: 200 mW/cm^2^			
β-TCP + MgO	43 wt%	Commercial: 57 wt% resin, shanghai guangyi chemical co.	DLP (custom)	_	S: 1500 °C for 3 h	[169,170]
		Dispersant: 4 wt% SPA				
	50 vol%	Monomers: HDDA + TPGDA (7:3)	DLP (Autocera-M)	_	S: 1250 °C for 2 h	[171]
		Initiator: 0.5 wt% TPO	LH: 25 μm			
		Absorber: 0.1 wt% P-hydroxyanisole	d: 12.4 mJ/cm^2^			
		Dispersant: Solsperse 41000 (Lubrizol)				
β-TCP + BG-58S (8:2)	45-60 wt%	Monomer: PEGDA	DLP	30.5–85.92 Pa s at 10 Hz	_	[53]
		Initiator: TPO	LH: 50 μm			
		Dispersants: DCA-1228 + PPG				
β-TCP + BG	52 vol%	Monomers: HDDA + OPPEA	DLP (CeraRay 1)	_	D: 370, 420, 460 °C for 2 h	[172]
		Initiator: 1 % TPO	LH: 100 μm		S: 710 °C	
β-TCP + Laponite	50-60 wt%	Monomer: PEDGA (200)	DLP	_	_	[52]
		Initiator: 0.5 % TPO	LH: 50 μm			
		Dispersant: DCA-1228	*λ*: 405 nm			
β-TCP + α-CS	55 vol%	Monomers: TPGDA + TMP3EOTA	SLA (3DCeram C900)	40–50 Pa s	S: 1100 °C for 3 h	[173]
		Initiator: 2,2-Dimethoxy-2-phenylbenzene	LH: 50 μm			
		Dispersants: KH-560 (3–5 %) + KOS110 + Ammonium polyacrylate	*λ*: 355 nm			
			P: 128 mW			
TCP (not specified)	60 vol%	Acrylate resin (not specified)	SLA (B9Creator)	0.1–1 Pa s at 1 Hz (<1000 cP)	_	[174]
		Dispersant: Surfactant Darvan C (Vanderbilt, USA)				
BCP (15/85)	_	Monomer: Commercial resin type B-0#, Ten Dimensions Technology	DLP (Autocera-L)	_	S: 1200 °C	[175]
		Initiator: TPO	*λ*: 405 nm			
		Dispersant: Solsperse 17000	I: 24.5 mW/cm^2^			
			LH: 25 μm			
BCP (1:1)	35 vol%	Monomer: HDDA	DLP (3DP-21ODS)	0.47–0.10 Pa s at 0.1–100 Hz	S: 1200 °C for 3 h	[113]
		Initiator: 1.5 wt% BAPO (Omnirad 819)	LH: 25 μm			
		Dispersant: 4 wt% BYK 2001	*λ*: 405 nm			
		Diluent: 40 wt% Camphor	I: 16.4 mW/cm^2^			
	40-70 vol%	Monomers: HDDA + PMMA (as porogen agent)	DLP	0.26–0.55 Pa s at 10 Hz	D: 600 °C	[112]
		Initiator: 2 wt% PPO (BAPO)	LH: 100 μm		S: 1200 °C for 3 h	
		Absorber: 4 wt% benzopurpurin 4B				
		Dispersant: Disperbyk 2001				
		Diluent: 40 wt% Camphor				
	70 wt%	Monomers: IBOA, HDDA, PEGDA (1:3:1)	DLP	0.8 Pa s at 40 Hz (<3 Pa s)	D: 700 °C	[176,177]
		Initiator: 1 wt% TPO (regarding resin)	d: 10 mJ/cm^2^		S: 1200 °C for 2 h	
		Dispersant: 4 wt% BYK 111 (regarding ceramic)	LH: 35 μm			
	65 wt%	Monomer: 28.86 wt% HDDA	DLP (Asiga Max)	400 mPa s at 50 Hz	S: 1100, 1200, 1300 °C	[47]
		Initiator: 1 wt% TPO				
		Dispersant: 9 wt% BYK 111				
BCP (6:4)	40-60 wt%^a^ _	Monomers: UDMA + camphene-camphor (ratio 2:1)	DLP	_	S: 1250 °C for 3 h	[178]
		Initiator: 2 wt% TPO	LH: 220 μm			
		Dispersant: 3 wt% KD4 (Croda, Everberg, Belgium)				
		Commercial: acrylic monomers, trade secret of Genoss®	DLP (Cubicon Lux)	_	_	[179]
			LH: 50 μm			
	50 wt%	Commercial: polyfunctional acrylic resin (Sirris, belgium)	SLA (Optoform)	_	S: 1125 °C, for 5 h	[180]
			LH: 50 μm			
	64 wt%	Monomers: Acrylic monomers (proprietary info)	DLP (Cubicon Lux)	_	S: 1250 °C for 10 h	[181]
		Initiator: TPO				
	40 wt%	Monomers: HDDA + TPGDA (7:3)	DLP (Autocera-M)	<5 Pa s over 60 Hz	S: 1100 °C for 2 h	[100]
		Initiator: 0.5 wt% TPO	LH: 50 μm			
		Absorber: 0.1 wt% MEHQ	d: 12.5 mJ/cm^2^			
		Dispersant: 4 wt% Solsperse 41000 (Lubrizol)				
BCP (7:3)	20 vol%	Commercial: resin FA1260T; SKCytec	DLP (pMSTL) (custom)	_	S: 1400 °C	[182]
	65 wt%	Photosensitive resin (not specified)	DLP (Admaflex 130+)	_	D: 800 °C for 2.7 h	[183]
			LH: 50 μm		S: 1100 °C for 5 h	
	_	Monomers: UA + PEGDA (Mn400) (3:1)	DLP (Admatec 130)	_	S: 1050 °C for 2 h	[111]
		Initiator: TPO	*λ*: 405 nm			
		Dispersant: BYK 2155				
BCP	50 wt%	Not specified + toners as pore forming agents	DLP (Autocera-M)	_	_	[184]
		Dispersant: MAEP	LH: 50 μm			
			*λ*: 405 nm			
	50 wt%	Not specified + 2 wt% toners as pore-forming agents	DLP (Autocera-M)	3 Pa s at 30 Hz^a^	S: 1100 °C for 2 h	[185]
		Dispersant: MAEP	LH: 50 μm			
			*λ*: 405 nm			
	50-60 wt%	Monomer: HDDA	DLP (Autocera-M)	5 Pa s at 30 Hz^a^	S: 1100 °C for 2 h	[54]
		Initiator: BAPO	*λ*: 405 nm			
		Dispersant: steric acid, sebacic acid, OA, MAEP	I: 10–34 mW/cm^2^			
	50 wt%	Monomer: HDDA	DLP (Autocera-R)	_	D: up to 466 °C	[91]
		Initiator: 2 wt% Irgacure® 819 (BAPO)	*λ*: 405 nm		S: 1250 °C	
		Dispersant: 5 wt% Solsperse 41000	I: 5 mW/cm^2^			
		Absorber: 2 wt% MEHQ	LH: 30 μm			
	45 wt%	Monomers: HDDA + TMP3EOTA	DLP (Autocera-M)	_	D: up to 550 °C	[55]
		Initiator: BAPO (Omnirad® 819)	*λ*: 405 nm		S: 1100 °C	
		Dispersant: Disperbyk 111	I: 3.1–7.4 mW/cm^2^			
BCP (6:4) + BG	30 vol%	Monomers: HDDA + TPGDA + PEG (54:23:23)	DLP (Autocera-M)	_	D: up to 650 °C for 1 h	[186]
		Initiator: 0.5 wt% TPO (regarding resin)	*λ*: 405 nm		S: 1200 °C for 2 h	
		Dispersant: 5 wt% Solsperse 41000 + 1 wt% RAD2500	LH: 50 μm			
		Absorber: 0.1 wt% Easepi 590				
BCP + BG 45S5®	40 vol%	Monomers: HDDA + TPGDA	DLP (Autocera-M)	0.1–2.2 Pa s at 20 Hz	S: 1200 °C for 2, 4, 6 h	[57]
		Initiator: TPO	LH: 50 μm			
		Absorber: MeHQ	*λ*: 405 nm			
		Dispersant: Solsperse 41000	d: 12.54 mJ/cm^2^			
α-TCP	45 wt%	Commercial: 50 % SP700 photosensitive acrylic resin	DLP (Shaoxing)	_	S: 1240 °C	[110]
		Dispersant: 5 wt% BYK 111	LH: 50 μm			
Ca_2,5_Na(PO_4_)_2_	_	Monomers: Laromer 8889 + HDDA	DLP (Ember)	_	S: 1200 °C for 12 h	[114]
		Initiator: TPO-L	LH: 30–50 μm			
		Absorbers: Sudan II orange + Carbon black	d: 170 mJ/cm^2^			
		Dispersant: Triton X-100				

**Abbreviations**: α-β-TCP: tricalcium phosphate, α-CS: α-calcium silicate, β-CEA: β-carboxyethyl acrylate, ACMO: acryloylmorpholin (4-(1-oxo-2-propenyl)-morpholine), AK: akermanite, AM: acrylamide, BAPO: phenylbis(2,4,6-trimethyl-benzoyl)-phosphineoxide, BCP: biphasic calcium phosphate, BG: bioglass, CEA: β-carboxyethyl acrylates, CQ: camphorquinone, EDMD: ethanone, 2,2-dimethoxy-1,2-diphenyl, HA: hydroxyapatite, HA-DA: hyaluronic acid-dopamine, HDDA: 1,6-hexanediol diacrylate, HEMA: 2-hydroxyethyl methacrylate, IBOA: isobornyl acrylate, MAEP: monoalcohol ethoxylate phosphate, MBAM: N-N′ methylenebisacrylamide, MeHQ: p-hydroxyanisole, OA: oleic acid, OPPEA: 2-([1,1′-biphenyl]- 2-yloxy) ethylacrylate, PEG: poly(ethylene glycol), PEGDA: poly(ethylene glycol) diacrylate, PMMA: poly(methyl methacrylate), PPG: polypropylene glycol, PPO: phenylbis (2,4,6-trimethylbenzoyl) phosphine oxide, SPA: sodium polyacrylate, SPMA: sodium polymethacryate, TAEA: tris(2-Hydroxyethyl) amine, TGDA: tetraethylene glycol diacrylate, TMP3EOTA: ethoxylated trimethylolpropane triacrylate, TMPTA: trimethylol-propane triacrylate, TPGDA: tripropylene glycol diacrylate, TPO: diphenyl(2,4,6-trimethylbenzoyl)phosphine oxide, TPO-L: ethyl phenyl(2,4,6-trimethylbenzoyl)phosphinate, TTA: trimethylolpropane trimethacrylate, UA: urethane acrylate, UDMA: diurethane dimethacrylate.

**Commercial chemicals**: Irgacure® 819/Omnirad® 819 (BAPO): phenylbis(2,4,6-trimethyl-benzoyl)-phosphineoxide, KH-550: 3-aminopropyl triethoxy silane, KH-560: γ-glycidyloxy-propyltrimethoxy silane, KH-570: γ-methacryloxy-propyltrimethoxy silane, Photocure-1173: 2-hydroxy-2-methylpropiophenone, SR454NS: ethoxylated (3) trimethylolpropane triacrylate, Triton® X-100: t-octilfenoxipolietoxietanol, Zelec *P*312: alcohol phosphates.

a Values taken indirectly from graphs and not explicitly described on article's text.

#### 3.1.3 Photoabsorbers

One intrinsic challenge of VP is the precision of the projected light. Light must travel through different materials, from the light source to the tank's bottom film, and finally to the photosensitive resin contained in the building gap. These materials present different refraction indices causing changes in the behavior of light waves. These changes can cause reflection, refraction, and finally scattering phenomena, with loss of light directionality and spreading of the light beam spots [30], thus reducing printing precision. Light-absorbing molecules (photoabsorbers), which attenuate the light scattering are often added to improve the printing resolution and pattern fidelity [30], although they might require longer exposure times and higher photoinitiator concentrations [84]. Improved printing resolution is obtained by a balance between these additives. Typical absorbing molecules include Orasol Orange G (absorbing λopt.range 480–500 nm) [[65], [66], [67], [68], [69]], Quinoline Yellow (absorbing λopt_range 400–450 nm) [85], Tartrazine (absorbing λopt.range 425–450 nm) [86], and Sultan I (absorbing λopt.range 385–415 nm [87].

Resin composition and printing parameters are intrinsically dependent and thus, they need to be tightly balanced to obtain optimal printability. Two key indicators often used to assess optimal printability are cure depth (Cd) and resin viscosity. Cure depth is the farthest point where the light is able to cure the photosensitive resin, thus is highly dependent on the interaction between light and resin. These interactions can be modified either by tailoring the resin formulation or by adjusting the light exposure parameters such as energy, intensity or exposure time, depending on the printing device. To ensure printability, the distance between layers (layer height) must be smaller than Cd.

The reactivity of a resin is commonly evaluated by curing it in a build plate-free volume, where the light penetration is unlimited. By varying the energy dose (i.e., light intensity) and exposure times, different thicknesses of cured resins are obtained. The Cd is represented versus the logarithm of the exposure times while keeping the light energy constant, or versus the logarithm of the light intensities while keeping the layer height constant. These calculations allow identifying the critical energy dose, according to Jacobs's equation (Equation (2)), characteristic of each resin formulation [88]. This empirical equation is commonly used in the literature to analyze experimental data on cure depth to determine the depth of penetration (Dp) and the empirical constant of critical energy (Ec). These values are then used to determine the layer thickness of each layer for light-based fabrication [89].

Equation 2 $$C_{d}=D_{p}\cdot \ln\left(\frac{E_{i}}{E_{c}}\right)$$

where Cd represents the cure depth, E_i_ is the energy dosage per area, E_c_ represents the “critical” energy dosage, and D_p_ refers to the “depth of penetration” of the laser beam into the solution, which is inversely proportional to the molar extinction coefficient and the concentration of photoinitiator.

Finally, a key parameter in VP approaches is the rheological behavior of the resin. Resins require a shear-thinning behavior which implies that the resin's apparent viscosity decreases when the shear stress increases, and is due to shear-induced disentanglement of the long polymeric chains. The polymeric chains, which are entangled at rest, align upon shearing, reducing the internal resistance to flow and, thus, its viscosity [90], ensuring printability [91]. Shear-thinning behavior favors an easier flow of the resin underneath the build plate, allowing layer-by-layer printing. This type of characteristic behavior is described by the Herschel-Burkley model (Equation (3)), which allows calculating some rheological parameters, such as the yield stress ($\tau_{0}$), which can be used to characterise the properties of the VP resins.

Equation 3 $$\tau =\tau_{0}+k\gamma^{n}$$

### 3.2 Calcium phosphate-loaded photocurable resins

The addition of CaPs into VP resins affects key parameters needed for printability such as the rheological properties of the resins and the light interactions with the light sources. Light penetration is hindered by the suspended particles causing light scattering and light absorption [92]. As a result, these changes in light interaction affect the cure depth (Equation (2)) of the resins. On the other hand, CaPs addition to the resin highly affects the resin's rheological behavior. Resin viscosity increases with the addition of ceramic fillers and can compromise printability. Furthermore, the ceramic filler hydrophilicity, when combined with common hydrophobic resin monomers, can cause agglomeration and sedimentation [93]. The main effect due to the incorporation of ceramic particles is on the shear-thinning behavior of the resins, typically increasing their yield stress ($\tau_{0}$, see Equation (3)). However, high yield stress is commonly considered to be an obstacle to the spreading of new layers and the yield stress tends to rise with increasing solid content [94].

A strategy widely used to modify the rheological properties and suspension stability in ceramic-loaded resins is by adding dispersant molecules which help stabilize the viscosity during the printing process. Dispersants form a protective film on ceramic particles preventing particle collision and maintaining the resin viscosity stable [60]. As dispersant molecules start to adsorb on the surface of ceramic particles, the repulsive forces between particles increase, reducing the viscosity of the slurry. Nonetheless, there is a limit to dispersant adsorption on ceramic particles due to the limited number of dispersant molecules that can be adsorbed onto their surface [41]. When excessive dispersant is added, excess dispersant results in flocculation and subsequent viscosity increase [47,91,95]. This phenomenon also limits the ceramic loading capacity. Generally, the reported viscosity limit for slurries ranges from 3 Pa s [39,43,62,[96], [97], [98]] to 5 Pa s [54,86,99,100], although higher viscous slurries have been successfully used [[101], [102], [103]]. Commercially available dispersants, such as commercial Solsperse® variants [57,60,64,104,105], BYK® variants [47,49,50,102,[106], [107], [108], [109], [110], [111], [112], [113]], or common surfactants such as Triton® X-100 [51,114], or sodium polyacrylates [45,46,101,115,116], beyond others, are used to lower the viscosity of the slurry, tuning their printability.

**Fig. 2:**
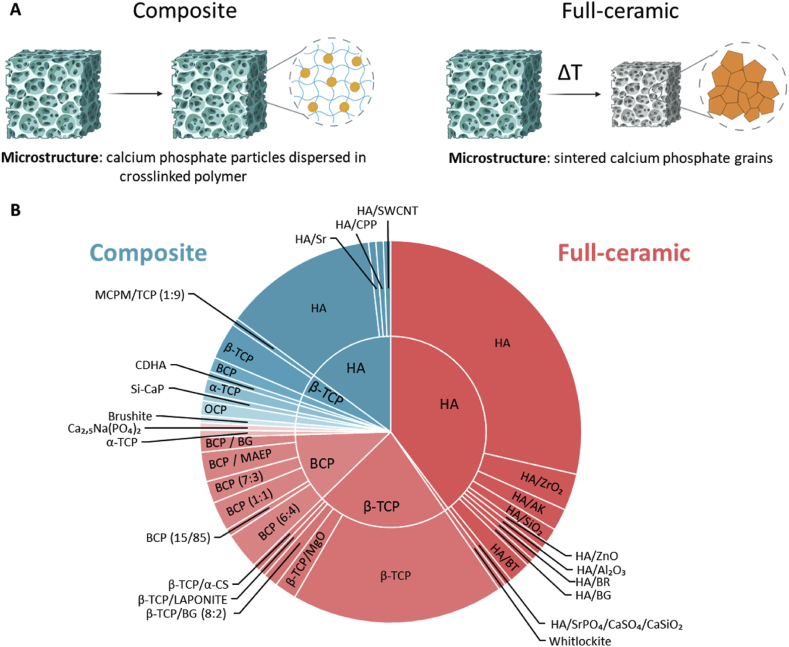
(A) Schematic representation of post-printing processes resulting in composite or full ceramic parts. Composite scaffolds, consist of a continuous polymeric matrix containing dispersed ceramic particles. Full ceramic scaffolds are obtained through a high-temperature treatment consisting in debinding and sintering. The resulting microstructure consists of ceramic particles bound together. (B) Schematic representation of different calcium phosphate ceramics and other inorganic components used on both the full-ceramic route (in red) and composite route (in blue), following Table 2, Table 3 (Abbreviations: α-CS: α-Calcium silicate, AK: akermanite, BCP: biphasic calcium phosphate, BG: bioglass, BR: bregidite, BT: barium titanate, CPP: calcium pyrophosphate, HA: hydroxyapatite, MAEP: monoalcohol ethoxylate phosphate, MCPM: mono-calcium phosphate monohydrate, OCP: octacalcium phosphate, Si-CaP: silicon-calcium phosphate, SWCNT: single-walled carbon nanotube, TCP: tricalcium phosphate).

In the case of calcium phosphates, the most commonly used ceramic fillers are hydroxyapatite (HA), β-tricalcium Phosphate (β-TCP), and a combination of both known as biphasic calcium phosphate (BCP) [60,113,[117], [118], [119], [120]]. In fact, over 90 % of the reviewed literature use these three CaPs, purely or together with other ceramic fillers such as zirconia (ZrO_2_), magnesium oxide (MgO), and bioglass, among others (Fig. 2B).

## 4 Fabrication routes

The resin composition, formulation and printing configuration are closely linked to the fabrication route used. In the literature, two main approaches are identified: producing fully ceramic scaffolds or polymeric-ceramic composite scaffolds. The resin composition and characteristics vary depending on the selected route. In the case of full ceramic scaffolds, the polymeric resin serves a critical role in the printing process but is subsequently removed through high-temperature treatments. Careful consideration of the debinding and sintering processes is essential to ensure structurally robust ceramic frameworks. In contrast, composite scaffolds maintain the polymeric phase, which remains an integral part of the final structure.

Once printed, the scaffolds can be further processed to adjust their physicochemical, mechanical or structural properties. Full ceramic bodies can be obtained by applying a thermal treatment that includes a debinding and a sintering step. The polymer matrix is removed during the debinding process, and the ceramic particles are fused together by solid-state diffusion during the sintering step. Alternatively, the polymeric matrix can be maintained, resulting in a composite scaffold based on a continuous polymeric matrix with dispersed ceramic particles (Fig. 2A). These two processing routes result in two distinct types of scaffolds, with different properties mainly in terms of mechanical and biological response. The first route is the most commonly followed, accounting for 75 % of the analyzed publications. Conversely, only 25 % of the published studies have investigated composite scaffolds (Fig. 2B).

### 4.1 Full ceramic scaffolds

Full ceramic scaffolds are obtained by removing the polymeric matrix and sintering the ceramic particles through thermal treatment. In this approach the polymeric resin is used as a sacrificial support, and the final goal is to obtain a full-ceramic part.

Commonly, the thermal treatment consists first of a debinding step to decompose the polymeric matrix. The temperature applied is usually slightly above the degradation temperature of the polymer used in the resin. The debinding temperature is often determined by Thermogravimetric analysis (TGA-DTG), which allows to identify the temperature range where the crosslinked polymer decomposes, and a thermal treatment is designed including a slow heating ramp and a dwell time to ensure total polymer debinding and simultaneously preserve the structural integrity of the scaffold [113,121]. Debinding steps are usually programmed with several steps including dwelling times at each step which ensures a homogeneous decomposition of the polymer without affecting the structural integrity of the scaffolds. After eliminating the polymeric phase, the scaffolds undergo a sintering process at high temperature to allow for solid-state grain boundary atomic diffusion, resulting in the consolidation of a full-ceramic part. The sintering temperature, heating ramp, and dwell times affect the grain growth and have a clear effect on the resulting microstructure. This process is commonly set depending on the material to avoid cracks or inconsistent gaps and undesired porosity. Low sintering temperatures lead to loose grains resulting in an increased number of pores, whereas higher temperatures promote grain coarsening, abnormal growth, and may even cause ceramic cracking and failure [41]. Resin formulations and processing parameters reported in the literature for the fabrication of full CaP ceramic scaffolds by VP are summarized in Table 2, including several commercially available CaP-resins, such as Lithabone® from Lithoz GmbH [103,[122], [123], [124], [125], [126]], Dental resin DETAX® [127], Cerhum® [128,129], for which the exact composition is often confidential [40]. Abbreviations can be found in footnotes bellow the table or in Table S1 (supplementary information).

### 4.2 Composite scaffolds

The high brittleness of full ceramic CaP scaffolds has encouraged the development of composite CaP specimens [157]. These composites must meet certain requirements of biocompatibility and resorbability. Therefore, the polymers used in the resin formulation should also be biocompatible and bioresorbable, degrading upon contact with organic fluids, and disappearing completely from the organism once the defected area is healed without any acidic degradation by-products [65]. In this approach the crosslinked polymeric structure is preserved, resulting in a composite material where a polymer matrix embeds ceramic particles that act as reinforcing agents. Polymer-ceramic printed scaffolds offer a promising solution for bone grafts, combining the strength and flexibility of both components. In this context, the photocrosslinkable resin is no longer a sacrificial phase and becomes an integral part of the final scaffold.

Commonly used resins in this particular approach entail the use of acrylated groups capable of reacting upon light irradiation. However, acrylated resins can exhibit high irritancy levels or even cytotoxicity in the uncured state. The leaching of unreacted monomers/oligomers or photoinitiators, as a result of low double-bond conversion rates, which is used as an indicator for the extent of the reaction, may cause health risks. Chen et al. illustrated the cytotoxicity problems associated to low crosslinking degrees, the concentration of the photoinitiator being a key factor influencing photopolymerization efficiency. When the photoinitiator concentration was too low, the energy to trigger the polymerization reaction was insufficient, leading to unpolymerized monomers, which can have toxic effects. Conversely, if the concentration was too high, excessive light absorption caused rapid polymerization of the surface layers, resulting in incomplete polymerization [73,99,131]. This phenomenon has been already addressed previously. Lee et al. demonstrated the existence of a critical photoinitiator concentration for which the curing depth is maximized for photopolymerization reactions. They reported experimental evidence that there must clearly be an “optimal” concentration to maximize the curing of the gel. In fact, when the photoinitiator concentration is low, just a small fraction of the photons is absorbed, resulting in few free radicals to start the reaction which are unable to form a gel. However, when the concentration of photoinitiator increases, the resulting radical initiation increases which results in higher double-bond conversion. At high photoinitiator concentrations, the photon absorption is so intense that light penetration diminishes, remaining confined near the surface of the resin. This fact induces the formation of a tightly cross-linked, thin layers [89]. To add up, this phenomenon is further aggravated by the presence of ceramic particles in the resin, which absorb part of the light radiation.

The printed parts must be thoroughly rinsed and ultrasonically cleaned, to wash out unpolymerized monomers or loose particles. In some cases, additional curing of the printed structure is carried out to completely crosslink any unpolymerized residue [86,98,187,188]. Moreover, in the case of reactive ceramics that are able to undergo a self-hardening process by a cement-like reaction, a subsequent process can be applied to transform the ceramic phase, as reported by Oliver-Urrutia et al. for an α-TCP-loaded resin [189]. A detailed summary of resin formulations and processing parameters reported in the literature for the fabrication of CaP/polymer composite scaffolds by VP are summarized in Table 3. Abbreviations can be found in footnotes bellow the table or in Table S1 (supplementary information).

## 5 Mechanical performance

Mimicking bone anisotropy and hierarchical structure remains a significant challenge. However, advances in the digitalization and processing of medical images such as those obtained from magnetic resonance imaging (MRI) or computed tomography (CT) have facilitated their integration into geometrical models suitable for implementation in advanced manufacturing techniques. This implementation has enabled the development of patient-specific designs and customization, but still, replicating the specific mechanical properties of natural bone persists as a hurdle in the clinical application of VP scaffolds. While the design and composition of the printed scaffolds are critical in determining their mechanical performance, the printing parameters also play a crucial role. The following sections examine and summarize the effects of printing parameters, designs, and compositions on the overall mechanical performance of VP printed scaffolds.

### 5.1 Effect of printing parameters on mechanical properties

The printing parameters set up during the printing process greatly affect the outcome of the printed parts. The energy dose, controlled by the light intensity and/or the exposure time has a strong effect on the polymerization process of the resins used in VP techniques. Higher polymerization degrees, achieved by higher energy dosage often result in higher stiffness in the polymeric phase. In addition, the printing orientation affects the mechanical properties of the scaffolds. Unlike other printing techniques where part orientations are highly restricted, VP printing offers a high versatility of printing orientation, allowing the anisotropy resulting from the printing process to be adjusted depending on the implantation site. Therefore, the printing orientation of the parts must be carefully designed to match the intended mechanical requirements of the printed scaffolds.

#### 5.1.1 Light exposure

Vat photopolymerization techniques are based on the interaction between light and the material. Consequently, the light energy dosage (which measures the intensity per time of exposure) is the main parameter affecting the integrity of the printed polymeric matrix and thus affects the mechanical properties of the scaffolds due to its role in the polymerization process. A higher energy dose, resulting from either longer exposure times or higher light intensity, leads to a higher energy absorption by photoinitiator molecules yielding more radical formation, and consequently a higher degree of double bond conversion. This creates a more crosslinked polymer network which generally improves the mechanical strength of the scaffold. Oppositely, low energy dose results in insufficient energy and uncured material, negatively impacting the crosslinking degree and lowering the scaffold's mechanical properties. Many commercially available resins are optimized for a certain energy dose and have specific parameters of light exposure times to print. However, and more specifically for composite scaffolds, formulated resins need to be optimized in terms of the energy dosage by varying exposure time or intensity. This effect can be seen in Table 2, and Table 3, where various energy dosages have been used depending on the resin formulation.

**Table 3:** Resin formulation, rheological properties and printing parameters for vat photopolymerization printing of calcium phosphate composite scaffolds. Acronyms (LSS: laser spot size, E: energy, d: dose, I: intensity, P: power, LH: layer height, *λ*: curing wavelength).

CaP	Loading	Slurry composition	VP printing	Slurry's viscosity	Ref.
HA	40 wt%	Monomer: TATO alkene + TATO thiol	SLA (Peopoly Moai 130)	_	[87]
		Initiator: 0.68 wt% TPO	LH: 60 μm		
		Absorbers: Sultan I + PYG	*λ*: 400 nm		
		Diluents: TMPMP, PETMP, ETTMP			
	10-20 wt%	Monomer: 60 wt% PEGDA (Mn = 700)	SLA (custom solid-oodle)	_	[190]
		Initiator: 0.5 wt% BAPO	LH: 400 μm		
		Diluent: PEG (Mw = 300)	*λ*: 355 nm		
			I: 40 mW/cm^2^		
	55 wt%	Monomer: OL-MA (Mn 1420 g/mol) + TEGDMA (1:1)	SLA	_	[187]
		Initiator: Irgacure® 819 (BAPO)	LSS: 100 μm		
			*λ*: 355 nm		
	40 wt%	Monomer: 60 wt% PEGDA (Mn 700)	SLA	5 Pa s at 100 Hz^a^	[131]
		Initiator: 0.5 wt% Irgacure® 2959	LH: 50 μm		
			*λ*: 355 nm		
			P: 70 mW		
	8 wt%	Commercial: acrylic-based urethane methacrylated resin (Novafab Powerdent Temp)	DLP (Novafab Vega)	_	[188]
			*λ*: 405 nm		
			I: 2.3 mW/cm^2^		
	5.2, 16.7 wt%	Monomer: 60–47.4-25.1 wt% PTMC-MA	SLA	57.5–71.9 mPa s	[65]
		Initiator: 5 wt% TPO-L	LH: 50 μm		
		Absorber: 0.15–0.12-0.08 wt% Orasol Orange G			
		Diluent: 40-47.4-58.2 wt% Propylene carbonate			
	20, 40 wt%	Monomer: PTMC-MA	SLA (Envisiontec Perfactory III)	_	[[66], [67], [68]]
		Initiator: 5 wt% TPO-L	LH: 50 μm		
		Absorber: (0.15–0.1-0.08 wt%) Orange G	I: 1.80 mW/cm^2^		
		Diluent: propylene carbonate			
	7 wt%	Monomer: PPF (70 %)	MSTL (SLA) (custom)	_	[191]
		Initiator: 1 wt% Irgacure® 819 (BAPO)	LH: 215 μm		
		Diluent: DEF (30 %)	*λ*: 375 nm		
			P: 310 mW		
	10 vol%	Monomer: PEGDA (Mw250) + AESO (1:1)	mSLA (Anycubic Photon)	0.2–0.49 Pa s at 50 Hz	[94]
		Initiator: 1 wt% Irgacure® 819 (BAPO)	LH: 50 μm		
	40 wt%	Monomer: OCM-2P	SLA (LS-250)	260 cSt	[192]
		Initiator: Irgacure® 671P	LH: 200 μm		
		Absorber: 0.02 wt% bis-(5-methyl-3-tert-butyl-2-oxyphenyl)-methane			
		Dispersant: PAA			
	20 wt%	Monomer: PDLLA	SLA (Envisiontec Perfactory Mini)	4–7 Pa s	[193,194]
		Initiator: 4 wt% Lucirin®-TPO-L	LH: 25 μm		
		Absorber: 0.2 wt% tocopherol inhibitor + 0.15 wt% Orange Orasol G	*λ*: 400–550 nm		
		Diluent: 50 wt% NMP	I: 17 mW/cm^2^		
	55, 75 wt%	Monomer: PLA-MA	SLA (custom)	_	[195]
		Initiator: ethylene glycoxide	LSS: 70 μm		
		Diluent: TEGDMA	*λ*: 355 nm		
			P: 1.5 mW		
	10 wt%	Monomer: PEDGA (60 or 40 %) RGD modified	SLA	_	[196]
		Initiator: Not specified	LH: 400 μm		
			I: 25–300 mW/cm^2^		
	10 w/v%	Monomer: 15 w/v% PEGDA + 10 w/v% GelMA + PLGA NPs with TGFb1	SLA (not specified)	_	[71]
		Initiator: 0.5 w/v% Irgacure® 2959			
	10 wt%	Monomer: 10–15 % GelMA	SLA	_	[70]
		Initiator: 0.5 % Irgacure® 2959	LH: 200 μm		
			E: 20 μJ at 15 kHz		
	2, 5, 10 w/w %	Monomer: 60 % PEGDA (Mn 700)	SLA (Printrbot®)	_	[197,198]
		Initiator: 0.5 % Irgacure® 819 (BAPO)	LSS 190 μm		
		Diluent: 40 % PEG	*λ*: 355 nm		
			Energy: 20 μJ at 15 kHz		
	30 w/v%	Monomers: GelMA + SilMA (1:1)	DLP (BP600, EFL)	_	[74]
		Initiator: 0.5 w/v% LAP	I: 15 mW/cm^2^		
		Absorber: 0.05 w/v% Tartrazine	LH: 50 μm		
	10-50 wt%	Monomers: 30 wt% GelMA	SLA (custom)		[73]
		Initiator: 4 % (w/v) Irgacure® 2959	P: 180–200 mW		
			LH: 100 μm		
	10-30 wt%	Monomer: mAESO + PEGDA (1:1)	mSLA (Sonic XL 4K, Phrozen)	_	[199]
		Initiator: 1 % Irgacure® 819	LH: 50 μm		
	5.5 wt%	Monomer: GelMA + AlgMA	DLP (EFL-BP-8601)	_	[75]
		Initiator: LAP	I: 14 mW/cm^2^		
			LH: 25 μm		
	5-10 wt%	Monomer: CSMA-2	DLP (Nobel Superfine, XYZ)	0.3–0.55 Pa s	[77,78]
		Initiator: 2 wt% BAPO	*λ*: 405 nm		
			I: 5.3–6 mW/cm^2^		
HA + CPP	5 + 5 wt%	Commercial: Soybean oil-based commercial resin (Anycubic Co.)	SLA (Anycubic Photon S)	0.501–0.839 Pa s	[200]
			*λ*: 355–410 nm		

HA + Sr	32.6–38 wt%	Monomers: 10 w/v% GelMA	DLP		[72]
		Initiator: 0.5 wt% LAP	*λ*: 405 nm		
			I: 12 mW/cm^2^		
HA + SWCNT	12.5 mg/mL^a^ + 0, 1, 2 wt%	Commercial: Dental resin, DITAX	DLP	_	[127]
		Dispersant: TEA	LH: 20 μm		
			*λ*: 360–410 nm		
β-TCP	32, 51, 60 wt%	Monomer: PTMC-MA	DLP (Envisiontec Perfactory III mini SXGA+)	_	[69]
		Initiator: 5 wt% TPO-L	LH: 50 μm		
		Absorber: Orasol Orange G	*λ*: 400–550 nm		
		Dispersant: Propylene carbonate	I: 7 mW/cm^2^		
	_	Monomer: GelMA + HyAc-MA	DLP (LumenX, Celllink)		[82]
		Initiator: LAP	LH: 50 μm		
		Absorber: R1800, benzophenone-9	*λ*: 400–550 nm		
			I: 7 mW/cm^2^		
	20 vol%	Monomer: 40 % PEGDA (Mw = 400)	DLP (MMSL) (Custom)	_	[201]
		Initiators: 0,25 wt% DAROCUR-1173 + DAROCUR- TPO (2:3)	LH: 100 μm		
		Dispersant: Quaternary ammonium	*λ*: 400–410 nm		
			I: 14,98 mW/cm^2^		
	5 mg/ml	30 w/v% PEGDA (Mn 700) + Chitosan (4 mg/ml)	SLA	100–200 mPa s	[202]
		Initiator: 0.5 w/v% Irgacure® 2959	*λ*: 365 nm		
			I: 1 mW/cm^2^		
	10 %	Commercial PLA resin (Yisheng New Material)	LCD-DLP (Chuangxiang LD-002R	200 mPa s	[203]
			LH: 50 μm		
	_	Monomer: PEGDA 508	SLA (Custom)	_	[42]
		Initiator: 0.5 % Irgacure® 2959	P: 100 mW		
BCP	22.5, 40 wt%	Monomer: 56 wt% PLLA + 20 wt% TMPTMA (crosslinker)	DLP (Kavosh economy)	0.1–5^a^ Pa s (<5 Pa s)	[86]
		Initiator: 4 wt% of TPO	LH: 50 μm		
		Absorber: 0.01 wt% tartrazine	*λ*: 405 nm		
		Diluent: 20 wt% of NMP	I: 18 mW/cm^2^		
	0.5, 1 w/v%	Commercial: resin Portux Print 3D Model (New stetic)	DLP (Wanhao Duplicator 7)	<3 Pa s	[98]
			LH: 45 μm		
			*λ*: 405 nm		
OCP	5 wt%	Monomer: PEGDA (50 %, 700Da)	DLP (Ember Autodesk)	_	[85,204]
		Initiator: 0.5 wt% Irgacure® 819 (BAPO)	LH: 200 μm		
			*λ*: 405 nm		
			I: 39.8 mW/cm^2^		
Brushite	10 wt%	Monomer: PEGDA	DLP (Ember Autodesk)	_	[85]
		Initiator: Irgacure® 819 (BAPO),	LH: 200 μm		
		TPO or Api-180 (0.1–1 %)	*λ*: 405 nm		
		Absorber: E104	I: 39.8 mW/cm^2^		
α-TCP	55.4 wt% (32 vol%)	Commercial: plant-based UV resin Anycubic Co.	DLP/LCD (Photon, MonoX Anycubic)	_	[189]
			LH: 75 μm		
			*λ*: 405 nm		
	60 wt%	Monomer: PEGDA (Mw 575) + PEGMA (Mw 350) (1:1 ratio)	DLP (Ember)	_	[92]
		Initiator: Irgacure® 819 (BAPO)	*λ*: 405 nm		
			I: 22–23 mW/cm^2^		
CDHA	10 vol%	Monomer: 45 vol% mAESO + 45 vol% TEGDMA	mSLA (Sonic XL 4K, Phrozen)	_	[205]
		Initiator: 1 wt% Irgacure® 819 (BAPO; regarding resin)	LH: 50 μm		
MCPM + TCP (1:9)	40, 50, 60 wt%	Monomer: CSMA-2	DLP (Nobel Superfine)	3 Pa s	[39]
		Initiator: 1 wt% CQ	I: 10 mW/cm^2^		
Si-CaP	0.5 mg/mL	Monomer: bdECM-MA	DLP (BP8601 Pro, EFL)	_	[76]
		Initiator: 0.25 % LAP	I: 20 mW/cm^2^		
		Cells: BMSCs (Bioprinting)	LH: 100 μm		
	_	Monomer: PEGDA	DLP (Custom)	_	[118]
		Initiator: TPO			
		Dispersant: DCA‐1228			
		Diluent: polypropylene glycol			

**Abbreviations**: α-β-TCP: tricalcium phosphate, AESO: acrylated epoxidized soybean oil, AlgMA: alginate methacrylate, BAPO: phenylbis(2,4,6-trimethyl-benzoyl)-phosphine oxide, BCP: biphasic calcium phosphate, CSMA-2: r((3R, 3aR, 6S, 6aR)-hexahydrofuro [3,2-b] furan-3,6-diyl) bis(oxy)) bis(ethane-2,1-diyl)) bis(oxy)) bis(carbonyl)) bis(azanediyl)) bis(3,3,5-trimethylcyclohexane-5,1-diyl)) bis(azanediyl)) bis(carbonyl))bis(oxy)) bis(ethane-2,1-diyl) bis(2-methylacrylate)), CPP: calcium pyrophosphate, CQ: camphorquinone, DEF: diethyl fumarate, E104: quinoline yellow food coloring, ETTMP: ethoxylated trimethylolpropane tri(3-mercaptopropionate), Gel-MA: gelatin-methacrylate, HA: hydroxyapatite, MCPM: mono-calcium phosphate monohydrate, NMP: N-Methyl-2-pyrrolidone, NPs: nanoparticles, OCP: octacalcium phosphate, OCM-2P: olygocarbonate dimethacrylate, OL-MA: methacrylated oligolactide, PAA: polyacrylic acid, PBS: phosphate-buffered saline, PDLLA: poly(DL-lactide), PEG: poly(ethylene glycol), PEGDA: poly(ethylene glycol) diacrylate, PEGMA: polyethylene glycol monomethacrylate, PETMP: pentaerythritol tetrakis(3-mercaptopropionate), PLA-MA: polylactic acid-methacrylate, PLGA: poly(lactic-co-glycolic) acid, PLLA: poly(L-lactide), PPF: poly(propylene fumarate), PTMC-MA: poly(trimethylene carbonate)-methacrylate, PYG: pyrogallol, RGD: arginine-glycine-aspartic acid peptide sequence, Si-CaP: silicon-calcium phosphate, SilMA: silk fibroin methacrylate, SWCNT: single-walled carbon nanotubes, TATO alkene: 1,3,5-tiallyl-1,3,5-triazine-2,4,6-trione, TATO thiol: tris[2-(3-mercaptopropionyloxy)ethyl]-isocyanurate, TEA: triethanolamine, TEGDMA: triethylene glycol dimethacrylate, TGFb1: transforming growth factor Beta 1, TMPMP: trimethylolpropane tris(3-mercaptopropionate), TMPTMA: trimethylolpropane trimethacrylate, TPO: diphenyl(2,4,6-trimethylbenzoyl) phosphine oxide, TPO-L: ethyl phenyl(2,4,6-trimethylbenzoyl)phosphinate.

**Commercial chemicals**: Api-180: 2-hydroxy-1-[3-(hydroxymethyl)phenyl]-2-methyl-1- propanone, Irgacure® 671P: 2,2-Dimethoxy-2-phenylacetophenone, Irgacure® 819/Omnirad® 819 (BAPO): phenylbis(2,4,6-trimethyl-benzoyl)-phosphineoxide, Irgacure® 2959: 2-Hydroxy-4′-(2-hydroxyethoxy)-2-methylpropiophenone, Lucirin®: ethyl phenyl(2,4,6-trimethylbenzoyl)phosphinate, DAROCUR-1173: 2-hydroxy-2-methyl-1-phenyl-1-propanone.

a Values taken indirectly from graphs and not explicitly described on article's text.

#### 5.1.2 Orientation

**Fig. 3:**
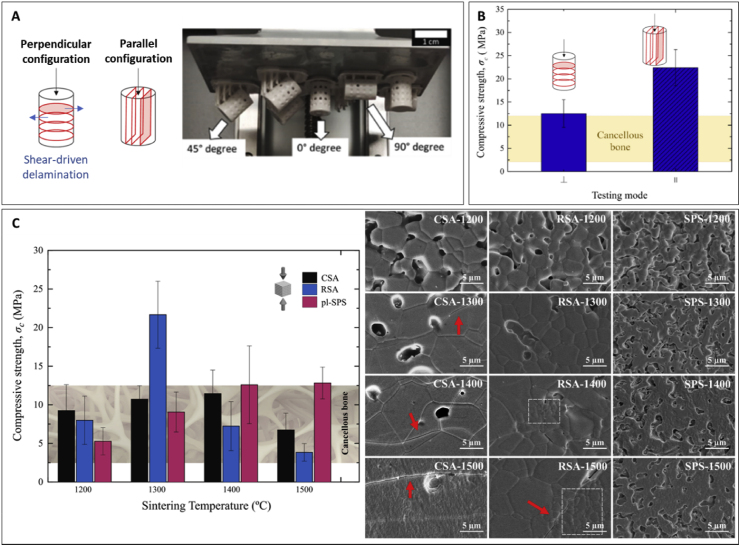
Effect of the selected printing parameters on mechanical properties; strategies proposed in the literature to improve the mechanical properties of calcium phosphate scaffolds obtained by VP. (A) the “stair-stepping” effect typically visualized in VP printing causes shear driven delamination/cracks when loaded perpendicularly to the printing plane. Photography of printed specimens at three different printing orientations, namely 0-, 45- and 90-degree angle. Figures taken from Ref. [117]. (B) In agreement with the later, β-TCP full ceramic scaffolds performed better when tested in the parallel configuration compared to the perpendicular one [156]. (C) Sintering method effects: conventional and advanced sintering methods such as Rapid Sintering in Air (RSA), and pressure-less Spark Plasma sintering (pl-SPS) have different effects on the microstructure and final compressive performance of β-TCP full ceramic scaffolds [160], red arrows pointing at microcracks.

Typical VP printed structures, based on layered configurations, often suffer the so-called “stair-stepping” effect, which refers to the individual layers stacking along the printing direction [156,158]. This effect leads to a notable anisotropy in the strengths of the construct between load configurations [157]. Specifically, layered structures are generally weaker when subjected to compression perpendicular to the orientation of the printed layers because defects across the interlayers promote shear-driven delamination. Paredes et al. reported that parts manufactured using VP techniques exhibit interlayer defects which results in the formation of local interlayer shear cracks, thus, reducing the strength during compression perpendicular to the printing plane (perpendicular configuration). In contrast, when tested parallelly to the printing plane (parallel configuration), the stacked layers collectively bear the load until a longitudinal crack propagates through one of the interlayers at the struts’ intersections (Fig. 3A). Although this results in catastrophic failure, the stress required to initiate crack propagation in this orientation is higher than that required for failure in the perpendicular orientation. Navarrete-Segado et al. reported a less pronounced but similar trend in full ceramic HA scaffolds. Compressive strength was higher when loads were applied parallelly or at a 45° angle to the printing direction (4.8 ± 0.2 MPa and 4.9 ± 0.3 MPa, respectively) compared to perpendicular loading (4.2 ± 0.4 MPa) [206]. Accordingly, in tests on β-TCP full-ceramic scaffolds, compressive strength in the parallel configuration (22 ± 4 MPa) was nearly double that of the perpendicular configuration (12 ± 3 MPa) [156] (illustrated in Fig. 3B). Another study showed similar results, with an average compressive strength of 37 ± 8 MPa in the parallel configuration (where the failure occurred abruptly) and 19 ± 4 MPa in the perpendicular configuration, where fractures progressed more gradually [158]. Therefore, it is important to consider the final implantation site and adjust the optimal printing orientation to withstand the highest loads.

#### 5.1.3 Thermal treatment: debinding and sintering

It is important to mention that in the particular case of full ceramic printed scaffolds, high-temperature treatments undoubtedly play a major role in their final mechanical performance. To start, the elimination of the polymeric phase of the scaffold, performed in the first step known as debinding, leads to the release of gases that produce microcracks and internal porosities, negatively affecting the structural and mechanical performance of the specimens. Furthermore, grain coarsening and the final consolidation of the piece, achieved during the second step known as sintering, also have a meaningful effect on its microstructure and resulting mechanical performance.

Specifically, the heating ramp and dwell times during the debinding step, as well as the temperature and dwell time during the sintering step, affect the grain growth and have a clear effect on the resulting microstructure. Temperature ramps are commonly set to increase slowly and are maintained at certain critical points where the mass change, as previously observed in TGA analysis, is highest for that specific material. The dwell time at those critical points is crucial to guarantee the proper controlled removal of the polymer, minimizing microcracks that could compromise structural integrity. Once the polymer is completely removed, the sintering process follows, playing a critical role in the structure's integrity [133]. A low sintering temperature leads to lose grains resulting in an increased number of pores, whereas excessive temperatures promote grain coarsening, abnormal growth, and may even cause ceramic cracking and failure [41], and the presence of second phases. Furthermore, the sintering dwell time also plays a key role in structural integrity. Guo et al. confirmed that a 2-h dwell during debinding and sintering resulted in optimal densification and mechanical properties of HA ceramic scaffolds. Without dwelling time, high microporosity led to weak intergranular bonding and low strength. Extending the dwell time to 4 h caused excessive grain growth and grain boundary cracks, reducing the compressive strength [145]. In addition, the heat treatment process itself plays a key role in the microstructure and, consequently, in the final structure's mechanical and biological properties [121,160].

Paredes et al. investigated the effects of conventional and advanced sintering methods, including Rapid Sintering in Air (RSA), and pressure-less Spark Plasma sintering (pl-SPS), on β-TCP ceramic scaffold at various sintering temperatures [160]. Their study highlighted how heat treatment parameters influence abnormal grain growth, the formation of microcracks (indicated by red arrows in Fig. 3C), the presence of an α-TCP second phase (associated with bimodal grain size distribution and the often relation to microcracks due to volumetric changes, shown as coarse dashed line areas in Fig. 3C), and undesired microporosity caused by poor densification. These factors play a critical role in the structural integrity of the scaffolds. They found that, with rapid sintering, the grain growth is inhibited facilitating surface diffusion and accelerating densification, which in turn is related to a lower amount of surface defects. This resulted in higher compressive strengths for the scaffolds sintered by the RSA method compared to those sintered by conventional routes.

### 5.2 Effect of scaffold composition on mechanical performance

**Fig. 4:**
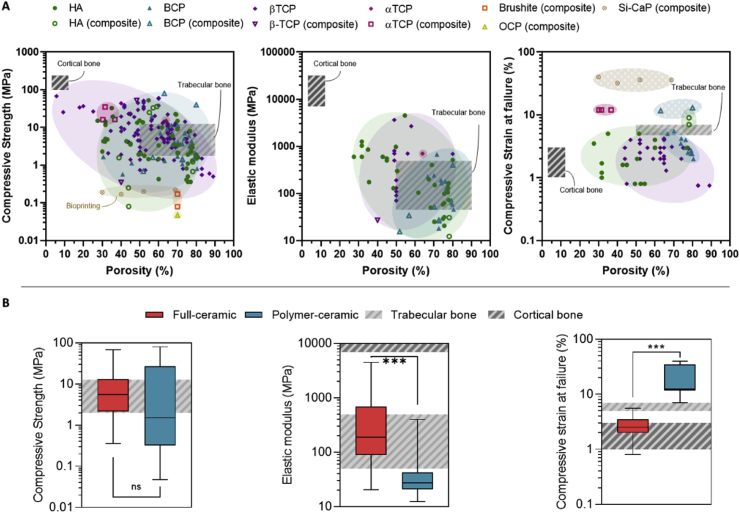
Mechanical properties of calcium phosphate ceramic and composite VP-printed scaffolds. (A) Compressive strength, elastic modulus, and compressive strain at failure as a function of the porosity of the scaffold. Each dot represents one condition. The dashed grey areas represent the values of natural bone (cortical and trabecular) obtained from Ref. [207]. (B) Box plots of all the data from the above graphs, grouped by processing strategies. Polymer-ceramic composites show improved strain at failure while maintaining adequate compressive properties. A normality test was performed, resulting in a non-parametric distribution; therefore, a Mann-Whitney test was conducted, revealing no significant differences in compressive strength (p > 0.05) and significant differences in elastic modulus and compressive strain at failure (∗∗∗p < 0.01).

The scaffolds’ composition is obviously a key parameter in their mechanical performance, together with biologically relevant pathways that will be later described. The mechanical properties of scaffolds as a function of their composition considering their overall porosity is represented in Fig. 4A. The numerical values based on the results reported in the literature are provided in Table S2 in the supplementary information. Compressive strengths and elastic moduli in the range of trabecular bone have been achieved for highly porous structures. It is well known that brittle materials when subjected to stress, break with little elastic deformation and near-zero plastic deformation. Consequently, brittle materials absorb relatively little energy before the fracture, even those of high strength [206]. This is the case of full ceramic scaffolds (represented in the graphs as filled symbols). Therefore, a rapid solution to mitigate the lack of strain energy is to print polymer-ceramic composite structures by creating ceramic slurries based on biocompatible prepolymer acrylic resins that serve as a matrix, with dispersed ceramic particles. Unlike full ceramic scaffolds, these composites (represented in the graphs as empty symbols) offer a higher deformation-uptake capability, where the polymeric phase can absorb higher energy yielding more flexible materials without compromising their compressive strength. A more direct assessment of the mechanical properties of full ceramic and composite scaffolds is presented in Fig. 4B, where the data from various studies are summarized in box plots and analyzed statistically using the Mann-Whitney test. This analysis indicated no significant difference in the compressive strength of the scaffolds. However, the intrinsic differences between full ceramic and composite scaffolds are evident in their elastic moduli and strain at failure. Full ceramic scaffolds are characterized by their stiffness, brittleness, and limited flexibility, with an average strain at failure of approximately 2 %, making them more challenging to handle. In contrast, the inclusion of a polymeric matrix in composite scaffolds enhances their strain at failure, achieving an average of around 10 %, thus improving their flexibility.

**Fig. 5:**
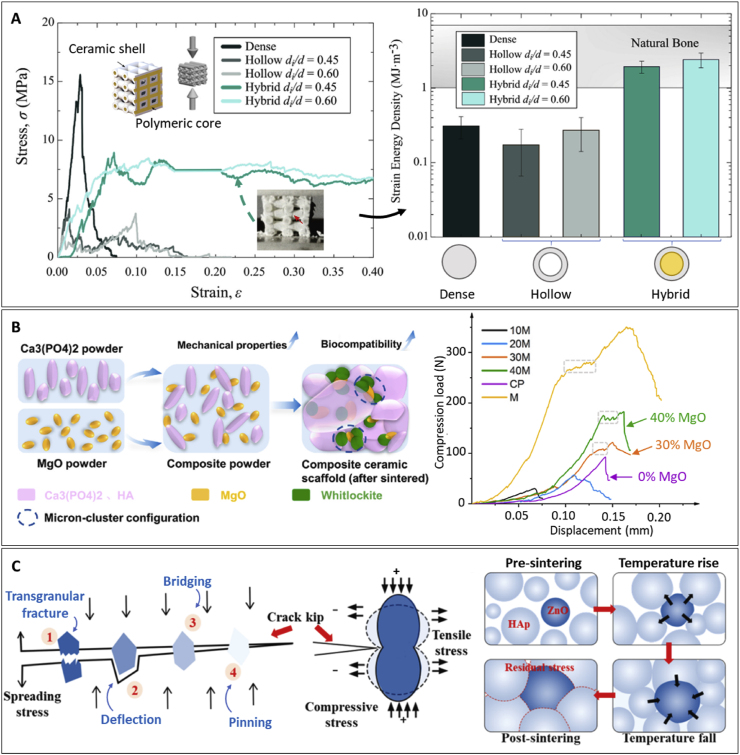
Effect of the scaffold's composition on mechanical properties; strategies proposed in the literature to improve the mechanical properties of calcium phosphate scaffolds obtained by VP. (A) when infiltrating a polymer (PCL) through the hollow internal canals/pores of a β-TCP full ceramic scaffold, creating a hybrid structure, its strain energy density further increases [157]. (B) Multicomponent printing, by the addition of other components (MgO) to the β-TCP initial powder. After sintering, whitlockite forms and further influences the mechanical properties [169]. (C) Fracture mode modification by incorporating ZnO as doping agent to HA ceramic scaffolds by dispersion of particles, piezoelectric properties, and residual stress toughening [147].

In addition to using biocompatible polymers as the structural matrix, researchers considered alternative strategies to improve the scaffold's flexibility and toughness. One strategy consists of infiltrating a polymeric phase into a hollow ceramic structure in order to create a hard yet less brittle phase-separated composite structure. Wu et al. demonstrated that these phase-separated composites exhibit strengths comparable to the fully dense-strut structure counterparts and enhanced toughness. These scaffolds resist loading even after the outer layer fractures under compression [208,209]. They printed β-TCP full ceramic scaffolds with an external macroporosity of 62.8 %. When subjected to compression testing, the hybrid scaffolds exhibited a compressive strength similar to fully dense scaffolds (approximately 15 MPa) but showed a notable improvement in strain energy density. A similar toughening effect was observed in bending tests. In a similar approach, Paredes et al. developed a similar co-continuous β-TCP/polycaprolactone shell-core composite obtained by infiltrating PLC into a hollow structure. They recorded enhanced toughening in both compression [157] and bending [158]. In the first study, while the hybrid scaffolds showed a slight reduction in compressive strength compared to dense scaffolds (from 11 ± 4 MPa to 9 ± 3 MPa), the strain energy density (a measure of toughness) improved by an order of magnitude, closely resembling the behavior of natural bone [157] (see Fig. 5A). It is important to note that in both studies the enhanced toughness in compression was recorded even after the scaffolds' ceramic shells began to fracture. The internal hybrid structures retained some integrity, allowing them to sustain additional loading.

Furthermore, the addition of ceramic/oxide phases to the calcium phosphates in the resin formulation has gained interest as a strategy to enhance scaffolds’ strength. Commonly used materials which act as second-phase ceramics are silicon oxides, zinc oxides, zirconia oxides, magnesium oxides/akermanite, bioglass, and to a smaller extent carbon nanotube (see Fig. 2B). The use of slurries consisting of a mixture of different agents has been performed mainly in full-ceramic routes containing high-temperature treatments. Thereafter, new phases can be obtained after sintering that enhance mechanical and biological properties. For instance, Ge et al. demonstrated that using MgO as co-ceramic filler with β-TCP, increased the compressive performance of composite scaffolds. After sintering, some of the MgO particles transformed into Whitlockite, a new ceramic phase which increased the compressive strength of the scaffolds when the amount of MgO achieved 30–40 %. The compression tests revealed that for a 50 % porous scaffolds, the compressive strength reached 4.49 MPa for the 40 % MgO-containing scaffolds compared to 2.26 MPa obtained with pure β-TCP ceramic scaffold (Fig. 5B) [169,170]. Moreover, the mechanical properties of HA scaffolds can be significantly enhanced by modifying their fracture mode. Gui et al. doped HA ceramic scaffolds with ZnO, demonstrating an improvement in their mechanical properties [147]. This improvement was attributed to three key factors: 1) dispersed ZnO reduced crack propagation through interfacial interactions such as transgranular cracking (also discussed in another study [145]), crack deflection, bridging, and pinning, increasing its hardness and strength; 2) ZnO generated an electrical charge under stress, transforming mechanical energy into electrical energy and enhancing fracture resistance; 3) due to differences in thermal expansion between ZnO and HA, residual stresses created during sintering and cooling restricted crack growth (Fig. 5C).

### 5.3 Effect of scaffold design on mechanical performance

The scaffold design, including the unit cell geometry and the overall scaffold architecture, is a crucial factor in the development of interconnected macroporous scaffolds as it directly influences and drives cell migration, vascularization, nutrient transport, and the mechanical stability required for successful bone regeneration and remodeling.

**Fig. 6:**
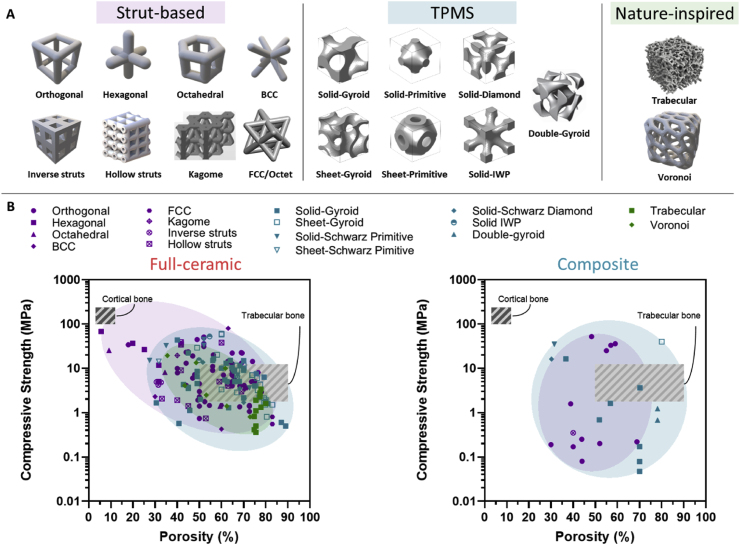
Pore geometry and mechanical properties of VP-printed calcium phosphate scaffolds. A) Different pore geometries, classified as strut-based, TPMS, and nature-inspired. Figures were taken from Refs. [122,157,210,211]; B) Compressive strength as a function of porosity classified as different pore geometries for full ceramic and composite scaffolds. The original data are reported in the Supplementary information. The values for natural bone (dashed grey areas) were taken from Ref. [207].

The advances in software processing have enabled the application of a wide range of different geometries that can be applied and further customized into the final printed scaffolds. In terms of geometrical designs, three main categories have been widely used in literature: 1) strut-based geometries; 2) Triply periodic minimal surface geometries; and 3) Nature-inspired geometries. Commonly used geometries are shown in Fig. 6A. The geometrical patterns can be obtained either by computer-aided designs (CAD) or by reverse-engineering, which involves the use of computer tomography (CT), or magnetic resonance imaging (MRI) to obtain a reconstructed 3D design [29].

The most common three-dimensional pore structures are the strut-based cells, also known as log piles, lattice-based, or orthogonal. The properties of these three-dimensional structures are directly related to the strut size, lattice shape, and cell arrangements. However, unlike the smooth, interconnected, and random arrangement of natural bone, they produce convex, self-intersecting rough shapes with no curvature. This makes strut-based cells suboptimal due to the abundance of stress concentration points [157]. In this line, researchers have also investigated the properties of inverse strut lattices, which are dense structures interpenetrated by linear pores, just like the negative of a strut-based lattice [60,97,206]. On the other hand, triply periodic minimal surface (TPMS) lattices are composed of infinite non-self-intersecting and periodic surfaces in three principal directions [210]. These structures are characterized as minimal surfaces, because the curvature along the principal curvature plane is equal and opposite at every point, making their overall curvature zero, and closely resembling bone natural surface. They can be designed as solid-TPMS, considering the volume bound by these minimal surfaces, hence filling out the volume inside them, or as sheet-TPMS, created by offsetting the minimal surface along its normal direction to generate a double surface, hence filling out the volume between the latter two. The smooth curvatures found in these arrangements eliminate the stress concentration often found in strut-based structures achieving mechanical properties close to trabecular bone [210]. Typical TPMS structures, used as solid or sheet-based, are Gyroid [86,98,100,118,212], Diamond [129,189], and Schwarz-primitive [54,106,130,190] among others. Finally, nature-inspired structures mimicking geometries found in nature have gained increasing interest. They can be obtained through tessellation during the scaffold modelling process. For instance, trabecular geometries consisting of trabeculae mimicking the cancellous bone hierarchy can be either obtained by CAD with Voronoi geometries [119] or CT scanned directly from natural bone [51], or sponges [130], through reverse engineering.

The mechanical performance of scaffolds is directly influenced by their design parameters. Among them, pore geometry is a critical factor extensively studied in the field of VP printing for CaP scaffolds. Fig. 6B illustrates the mechanical properties of scaffolds as a function of their porosity and pore geometrical designs, with data presented separately for full-ceramic scaffolds, and composite scaffolds. The numerical values derived from the literature are provided in Table S2 of the supplementary information. Most of the samples studied exhibit high porosity levels, ranging from 30 % to 80 %, close to the porosity of trabecular bone. However, less porous scaffolds, which mimic cortical bone, have also been developed, using mainly strut-based structures with porosities ranging from 5 to 25 %. In contrast, nature-inspired and TPMS geometrical designs seem to focus more on the trabecular bone zone. While both fabrication routes can achieve comparable compressive strengths (as seen in the previous section), the represented data reveal slight differences between these two routes. Full ceramic scaffolds generally show a reduction of the compressive strength with increasing porosity, which is a trend generally observed in porous materials [213]. Conversely, composite scaffolds show a wider dispersion of results with no clear correlation between porosity and compressive strength. This variability underscores that the lattice geometry of pores is not the only variable affecting the mechanical performance of the scaffolds. Instead, other factors, such as pore size and material composition, which are not considered in the graphs, play crucial roles. The latter is particularly significant for composite scaffolds, where the polymer matrix formulation can vary widely, contributing to the observed scatter in the results.

**Fig. 7:**
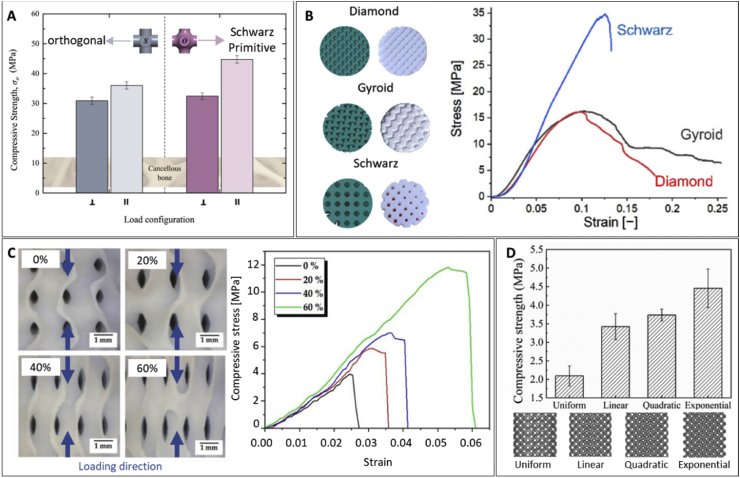
Effect of the scaffold's architecture on mechanical properties; strategies proposed in the literature to improve the mechanical properties of calcium phosphate scaffolds obtained by VP. Pore geometry plays an important role, (A) improving mechanical properties from convex-self intersecting orthogonal pattern to smooth Schwarz-TPMS of β-TCP full ceramic scaffolds [121], (B) Schwarz geometry outperforms gyroid and diamond counterparts for CDHA-epoxy composite scaffolds with similar porosities [189]. (C) Geometry modification, by elongating a BCP full ceramic gyroid structure yielded better compressive performance [113]. Blue arrows point the loading direction. (D) Porosity gradient can enhance its strength, as recorded in HA-AK full ceramic scaffolds [56].

Pore geometry, however, does significantly affect the mechanical properties of scaffolds when comparable scaffolds (in terms of materials and porosity variables) are analyzed. Paredes et al. have registered significant differences between two identical structures but with different geometry. In this study, a strut-based β-TCP full ceramic scaffold was compared with its Schwarz Primitive counterpart with equivalent porosity [121]. The smooth-curved Schwarz Primitive geometries demonstrated superior mechanical performance under compression compared to the strut-based geometries as seen in Fig. 7A. The smooth curvatures of these structures can effectively alleviate shear stresses in comparison to simpler geometries with intersecting edges, which can act as crack initiators. In another study, Oliver-Urrutia et al. compared different TPMS pore architectures with similar porosities for CDHA-Epoxy composite scaffolds; 36.7 % for the solid-gyroid, 30.2 % for the diamond, and 31.5 % for the solid-schwarz primitive. They found significant differences in mechanical properties, with the solid-Schwarz primitive structure exhibiting a compressive strength twice that of the diamond structure. This result indicates that, for comparable porosities, the compressive strength is significantly influenced by pore architecture (see Fig. 7B) [189].

In addition to selecting different pore geometries, another strategy to significantly enhance the mechanical performance of the scaffolds is by creating more complex pore architecture from already existing geometries. Lee et al. revealed that, by elongating the widely studied gyroid-TPMS pores, a remarkable increase of the compressive strength was obtained, from 4.3 ± 0.26 MPa to 11.5 ± 1.75 MPa, with an increase in elongation percentage from 0 to 60 % for BCP scaffolds [113] (see Fig. 7C). This suggests the possibility of enhancing the mechanical performance just by simple modifications of already-known structures. Another approach has been considered by creating gradient structures to TPMS geometries, mimicking the natural bone hierarchy, and thus promoting better mechanical properties. Deng et al. studied the effect of linear, quadratic, and exponential porosity gradients in gyroid-scaffolds. They reported an increase in compressive strength and elastic modulus for gradient-scaffolds compared to uniform ones (see Fig. 7D). More precisely, exponential gradient-scaffolds made with HA-AK (9:1 ratio) performed best of all four combinations, exhibiting twice compressive strength (from 2.10 ± 0.27 MPa to 4.45 ± 0.52 MPa) and elastic modulus (from 0.301 ± 0.036 GPa to 0.580 ± 0.034 GPa) than the other combinations [56].

## 6 Biological performance

Bone regenerative grafts act as sacrificial structures promoting tissue colonization, bone formation, and resorption while being replaced by new bone. Despite the vast research in biomaterials topic, there is a large discrepancy between the numbers of those reaching clinical application. Ascribed more to a lack of predictive power in the methods to test the biological performance than the biological performance itself, bone regenerative grafts must undergo a series of validations to ensure meeting clinical relevance. To comply with regulatory demands, scaffolds undergo extensive preclinical evaluations assessing cytotoxicity, bone formation capability, angiogenesis potential, and risks from biodegradation or manufacturing residues. Biomaterial validation entails both *in vitro* and *in vivo* studies. *In vitro* studies, usually performed following ISO-10993 standards, are aimed at testing the direct and indirect cytotoxicity of the scaffolds, besides other aspects like cell adhesion, proliferation, and osteogenic differentiation. This assessment can be performed using colorimetric assays (e.g., MTT, CCK8), staining methods (e.g., Alizarin Red), and osteogenic markers (e.g., ALP, RUNX2, OCN). On the other hand, vascularization, which is key in promoting bone regeneration and nutrient supply, is also assessed, especially in macroporous scaffolds. This is performed by examining endothelial progenitor cells (EPCs) for viability, migration, and differentiation via assays like tube formation and RT-PCR for angiogenesis-related genes (e.g., VEGF). *In vivo* studies involve implanting the scaffolds in orthotopic (bone) or ectopic (non-bone) sites in animal models such as rats, rabbits, and dogs. Bone formation is analyzed using histology, micro-CT, and biomechanical tests. Angiogenesis is assessed through immunohistochemical staining, angiography for 3D vascular reconstructions, and the measurement of percentage vascular volume. In/Ex ovo assays performed on a Chicken Egg Chorioallantoic Membrane (CAM) model, the vascularization potential can be assessed by measuring vascular density and bifurcation points on the embryonic membrane.

The most relevant reviewed outcomes in terms of biological evaluation of VP printed scaffolds are presented followingly, categorized as *in vitro* evaluations (Table 4), and *in vivo* evaluations (Table 5).

**Table 4:** in vitro evaluation studies of VP-printed calcium phosphate-based scaffolds reported in the literature (h: hour/s, d: day/s, w: week/s, m: month/s).

Ref	Assay	Cell type	Quantification assays	time
[87]	Indirect	hDF, Raw264.7	Alamar Blue (cytotoxicity)	24, 72 h
[86]	Indirect	L929	MTT	24, 48, 72 h
[153]	Indirect	mBMSC	qPCR of ALP, OCN, OPN, RUNX2 (osteogenesis genes)	7 d
			qPCR of CD31, VEGF, GAPDH	
[97]	Indirect	mBMSC	CCK8 (cytotoxicity)	3 d
[99]	Indirect	NCTC clone L929	CCK8 (cytotoxicity)	3 d
[131]	Indirect	MSCs	CCK8 (cytotoxicity)	1 d
[58]	Indirect	Primary rBMSC	CCK8 (cytotoxicity)	1 d
[98]	Indirect	L929	Cytotoxicity	3 d
[132]	Indirect	MC3T3-E1	Differentiation: Zn^2+^ released in medium detected using commercial kits	4 d
[179]	Indirect	L929	Microscope characterization	1, 2 d
[181]	Indirect	L929	Cytotoxicity	24, 48 h
[64]	Indirect	hBMSC	MTT (proliferation)	1, 3, 7 d
[114]	Indirect	NCTC L929	MTT (cytotoxicity)	1, 4 d
[171]	Indirect	rBMSC, EPC, RAW264.6	Sirius red staining, ARS, ALP, RT_PCR (osteogenic differentiation)	24 h, 3 d (PCR)
			Wound healing assay, Transwell assay, Tube formation assay, RT_PCR (angiogenic differentiation)	
[100]	Indirect	rBMSC	CCK8 (cytotoxicity)	1, 3 d
[137]	Indirect	MC3T3-E1	Immunofluorescence (cell survival)	24 h
[120]	Indirect	Mouse L929	MTT (Cytocompatibility)	1, 4, 7, 14 d
[166]	Indirect	rBMSC	(PCR: ALP, OCN, OPN, RUNX2), activation of MAPK	1, 7, 14, 21 d
[141]	Indirect	L929	MTT	24 h
[139]	Indirect	HUVEC	Scratch wound healing, cell migration, tube formation with 24 h extracts	6, 24 h
	Indirect	Rabbit BMSC	ALP, ARS, qRT-PCR (COL1, RUNX2, BMP2, OPN, SPP1), Staining	7, 10, 14 d
[140]	Indirect	HUVEC	Angiogenesis (tube formation, capillary length, ARS, crystal violet staining)	24 h
	Indirect	BMSC	Osteogenesis (ALP, ARS)	7, 14, 21 d
[147]	Indirect	MC3T3	Live/dead staining, CCK8 (viability), ALP	1, 3, 5 d
[76]	Indirect	BMSC	ALP, ARS staining, western blot (RUNX2, OSX, ALP, COL1, OPN, OCN), qRT-PCR (RUNX2, ALP, COL1, OPN, OSX, OCN)	3, 7, 14 d
	Indirect	RAOEC	CD31/DAPI, tube formation, western blot (TLR4, T-PI3K, T-AKT, P-AKT)	7 d
	Indirect	Macrophage	Western blot (iNOS, TNF-α, ARG-1, IL-10), flow cytometry	1, 3, 7 d
[175]	Indirect	BMSC	CCK8, Phalloidin staining, ALP, ARS	1, 2, 3, 4, 5 d
[97]	Direct	mBMSC	CAM/PI staining	7 d
[154]	Direct	MC3T3-E1	Not described	6, 12, 24, 48 h
[45]	Direct	MC3T3-E1	CCK8 (adhesion + proliferation)	1, 4, 7 d
[101]	Direct	MC3T3-E1	CCK8, DAPI, Ca deposition (ARS)	1, 3, 5 d (CCK8)
				24 h (DAPI)
				14 d (ARS)
[190]	Direct	hMSC	MTS (adhesion + proliferation), Quant-iT™ Picro-Green® dsDNA Kit, GAG, II collagen, Ca deposition reagent kit (differentiation)	4 h, 1, 3, 5 d (adhesion)
				1, 2 w (differentiation)
[187]	Direct	MC3T3-E1	RT-PCR (COL1, OPN, OCN)	21 d
[118]	Direct	BMSC	Adhesion (CCK8)	1, 3, 5, 7, 14 d
			Morphology and cytotoxicity (live/dead)	
			Early osteogenesis (ALP)	
			Mineral deposition (ARS)	
			Osteogenic gene expression (COL1, OCN, OPN, RUNX2)	
[58]	Direct	primary rBMSC	DAPI, Phalloidin (cell viability)	1 d
[98]	Direct	L929	Adhesion	8 d
[169]	Direct	MC3T3-E1	CCK8 (adhesion + proliferation)	1, 4, 7 d
[66]	Direct	hBMSC	Cytotoxicity	1, 7, 14, 21, 28 d
[67]	Direct	hBMSC	Adhesion + proliferation, ALP (cell differentiation), ARS (calcium deposition)	1, 7, 14, 21, 28 d
[132]	Direct	MC3T3-E1	Live/dead (cytotoxicity), CCK8 (proliferation), ALP (differentiation)	1, 4, 7 d (proliferation), 21 d (differentiation)
[46]	Direct	MC3T3-E1	DAPI (adhesion, proliferation, differentiation)	1, 4, 7 d
[178]	Direct	MC3T3-E1	DAPI (adhesion), MTS (cell proliferation)	1 d (adhesion)
				3 d (proliferation)
[180]	Direct	MG-63	MTS (cytotoxicity), DAPI-actin (morphology)	3 d
[191]	Direct	MC3T3_E1	CCK8 (adhesion + proliferation)	2 w
[108]	Direct	MC3T3-E1	Adhesion, CCK8 (proliferation), ALP (differentiation)	3 d (adhesion)
				1, 7 d (proliferation)
				7, 14, 21 d (differentiation)
[53]	Direct	rBMSC	CCK8	1, 7 d (adhesion)
				1, 3, 7 d (proliferation)
[106]	Direct	rBMSC	MTT (proliferation)	1, 4, 7 d
[111]	Direct	HUVEC	CCK8 & live/dead assay (adhesion)	1, 3, 5, 7 d
			qPCR (angiogenesis)	
[104]	Direct	rBMSC	CCK8, live/dead (adhesion, proliferation), ALP (differentiation), OPN, RUNX2, COL2, VEGFR2, vWF, CD31 (protein expression)	1, 4, 7 d
[107]	Direct	MC3T3-E1	CCK8 (adhesion + proliferation)	1, 3 d
[125]	Direct	U2OS, MSC	DAPI (adhesion), Glo^TM^-MT (proliferation), ALP + ARS (differentiation), RUNX2, Sp7, Spp1 (RT-qPCR)	66 h (proliferation)
				14–35 d (differentiation)
[94]	Direct	MC3T3-E1	XTT kit (adhesion, proliferation)	1, 3, 7 d
[39]	Direct	ADSC, MC3T3	Alamar blue (proliferation)	1, 7, 14, 21 d
			Phalloidin/DAPI staining (Viability)	
			RUNX2, COL1A1, OCN, OPN (qPCR)	
[171]	Direct	rBMSC, EPC, RAW264.7	DAPI (adhesion), CCK8 (proliferation), Calcium-AM/PI (Live/dead)	1, 3, 5 d
			Polarization of macrophages: immunofluorescence staining, RT-PCR	
			Osteogenic activity of BMSCs in MCM: Alizarin red	
			Angiogenic activity of EPCs in MCM	
[161]	Direct	rBMSC, hBMSC	DAPI (Adhesion), qPCR (qualitative osteogenic gene analysis (RUNX2, GAPDH, OCN, OPG)	3, 7 d
[193]	Direct	hMSCs	ALP, DNA	21 d
[122]	Direct	MC3T3-E1	pNPP (ALP activity), DAPI (cell adhesion), Quanti-iT PicoGreen assay (cell viability)	1, 7, 14 d
[214]	Direct	MC3T3-E1	Cytotoxicity (agar)	24 h
[182]	Direct	hTMSC	CCK8 (cytotoxicity)	1, 4, 7 d
[100]	Direct		Phalloidin/DAPI staining	1 d
[134]	Direct	hFOB	CCK8 (cytotoxicity & proliferation)	1, 4, 7 d
			qPCR: RUNX2, COL1, ALP, OPN, OCN (differentiation)	
			Actin/DAPI staining	
[184]	Direct	BMSC	Proliferation, migration, differentiation	1,3, 5 d (proliferation)
				1 d (migration)
				3, 7, 14, 21 d (differentiation)
[195]	Direct	MC3T3-E1	ALP (differentiation)	21 d
[135]	Direct	MC3T3-E1	Phase contrast light microscopy, SEM	2, 7, 14 d
[162]	Direct	rBMSC	Adhesion, proliferation	1, 3, 5, 7 d
[185]	Direct	BMSC	AlamarBlue, confocal, PCR	1, 3, 5, 7, 14 d
[47]	Direct	BMSC	Proliferation, apatite formation	1, 3, 5 d (proliferation)
				3 d (apatite formation)
[54]	Direct	BMSC	CCK8 (cytotoxicity & proliferation)	1, 4, 7 d
[55]	Direct	mBMSC	cell proliferation, ALP staining, live dead	1, 3, 5 d
[168]	Direct	hMSC	OIM, ALP, AR, OCN + qRT-PCR + angiogenic + proteomics	7, 14 d
[137]	Direct		MTT (cytotoxicity-proliferation)	12, 24, 36, 48 h
[110]	Direct	MC3T3-E1	CCK8 (cytotoxicity & proliferation)	1, 4, 7 d
			F-actin (adhesion)	
[49]	Direct	MC3T3-E1	CCK8 (cytotoxicity & proliferation)	1, 3, 7 d
[50]	Direct	BMSC	CCK8 (cytotoxicity & proliferation)	1, 3, 5 d
			Rhodamine-phalloidin/DAPI (adhesion)	
			qRT-PCR: RUNX2, COL1A1, BMP-2, OPN (differentiation)	
[120]	Direct	Mouse L929	DAPI, SEM (morphology)	1, 4, 7, 14 d
[116]	Direct	MC3T3-E1	SEM (adhesion)	1, 4, 7 d
			CCK8 (cell viability)	
[63]	Direct	mBMSC	Alamar blue + live dead (cell viability)	1, 3, 5 d
[42]	Direct	BMSC	ALP quantification (differentiation)	7 d
[167]	Direct	BMSC	Live/dead (viability)	1, 3, 5 d
			CCK (proliferation)	
[196]	Direct	MSC	MTS (proliferation)	1, 2, 3 w
			ALP (differentiation)	
[71]	Direct	hMSC	F-actin, DAPI (proliferation)	1, 2, 6 d
			PCR: RUNX2, ALP, OPN, Osteocalcin, COL1 (differentiation)	1, 2, 3, 4 w (PCR)
[70]	Direct	OB, MSC, BrCa (mono & co-culture)	Live/dead (cell viability)	1,3,5 d, 2 w (coculture)
			Alamar Blue (proliferation mono-culture)	
			MTS (proliferation co-culture)	
			CellTracker Green/orange (adhesion of co-culture)	
			ALP (differentiation)	
[197]	Direct	hFOB, MDA-MB-231	MTS (viability)	1, 3, 5, 7 d
		Co-culture	Red-X Phalloidin/DAPI (adhesion)	
			IL-8 ELISA Kit (Osteoblast cell function	
	Direct	hADSC	SEM (cell attachment)	1, 3, 7, 14, 21 d
			ARS (differentiation)	
			RT-qPCR (OCN, OPN, RUNX2)	
[198]	Direct	MCF-7/MB-MDA231	MTS (viability & chemosensitivity)	1, 3, 5 d
		Co-culture	Red-X Phalloidin/DAPI (adhesion and tumor growth)	
[141]	Direct	MG63	Live/dead	1, 3, 7, 14 d
[72]	Direct	Osteoblasts	MTS	1, 4, 7 d
[144]	Direct	RAW267.4	RT-qPCR	3, 5, 7 d
[139]	Direct	HUVEC	CD31/VEGF staining	5 d
	Direct	MC3T3	Cell viability (live/dead), adhesion (phalloidin/DAPI staining)	3, 5 d
[140]	Direct	MC3T3	CCK8 (cell proliferation)	3, 7 d
[95]	Direct	Rat BMSC	DAPI/FITC staining, CCK8	1, 3, 5 d
[145,152]	Direct	MC3T3	Rhodamine phalloidin/Hoechst 33342 staining, MTT, ALP	1, 3, 7 d (adhesion, MTT)
				7, 14 d (ALP)
[199]	Direct	dMC3T3-OB	Live/dead, XTT (proliferation), ALP (differentiation)	1, 3, 7 d
	Direct	dRAW-OC	XTT, TRAP staining, F-Actin/DAPI staining	7 d
[91]	Direct	rBMSC	CCK8, calcein AM/PI, SEM, ALP	1, 4, 7 d
[186]	Direct	rBMSC	CCK8, Phalloidin/DAPI, RT-qPCR (BMP2, ALP, OPN, COL1)	1, 3, 5 d (proliferation)
				7, 14 d (osteogenesis)
[146]	Direct	UC-MSC	CD31, PDGF-α staining (vascularization)	3 w
	Direct	UC-MSC	OCN, COL1 staining	1, 3, 4 w
[75]	Direct	BMSC	Live/dead staining, CCK8 (proliferation)	1, 4, 7 d
[74]	Direct	MC3T3-E1	CCK8, Phalloidin/DAPI staining (proliferation)	1, 4, 7 d
	Direct	Rabbit BMSC	ALP, ARS, PT.PCR	12, 18 d
[203]	Direct	MC3T3-E1	CCK8, Live/dead, hemolysis test	1, 3, 5 d
	Direct	MC3T3-E1	ALP staining, ALP, ARS, RT-qPCR (BMP-2, OCN, COL1, GAPDH), Phalloidin/DAPI staining, Elisa assay (osteogenesis activity)	7, 14 d
[55,215]	Direct	MC3T3-E1	Proliferation, ALP, ARS, PCR	1, 3, 5 d (proliferation)
				7, 14 d (ALP)
				14 d (PCR)
				14, 21 d (ARS)

Abbreviations: ADSC: adipose derived stem cell, ALP: alkaline phosphatase, ARS: alizarin red S, BMP-2: bone morphogenetic protein-2, BMSC: bone marrow stem cells, BrCa: breast cancer, CAM/PI: calcein-AM and propidium iodide solutions, CCK8: cell counting kit 8, CD31: cluster of differentiation 31, COL1: collagen type 1, DAPI: 2-(4-amidinophenyl)-1H -indole-6-carboxamidine, EPC: endothelial progenitor cells, GAG: glycosaminoglycans, GAPDH: glyceraldehyde-3-phosphate dehydrogenase, hBMSC: human bone marrow stem cells, hDF: human dermal fibroblasts, hFOB: human fetal osteoblast, hMSC: human mesenchymal stem cells, hTMSC: human turbinate mesenchymal stromal cells, HUVEC: human umbilical vein endothelial cells, IL-8: interleukin-8, MAPK: mitogen activated protein kinase, mBMSC: mouse bone marrow stem cell, MC3T3-E1: mouse calvaria cell line, MCF-7: breast cancer cell line, MDA-MB.231: breast epithelial cell line, MG-63: osteosarcoma derived cell line, MTS/MTT/XTT: cell viability assays, NCTC-L929: clone of mouse strain L, OCN: osteocalcin, OPN: osteopontin, PBS: Phosphate buffered saline, pNPP: nitrophenyl phosphate, RAW264,7: macrophage cell line, rBMSC: rabbit bone marrow derived mesenchymal stem cell, RT-qPCR: real-time quantitative polymerase chain reaction, RUNX2: runt-related transcription factor 2, SBF: simulated body fluid, SD rats: SPRAGUE DAWLEY®, SEM: scanning electron microscopy, Sp7: osterix, Spp1: osteopontin, TRIS-HCl: 2-Amino-2-hydroxymethyl-propane-1,3-diol, U2OS: human osteo-sarcoma cell line, VEGF: vascular endothelial growth factor, VEGFR2: vascular endothelial growth factor receptor 2, vWF: Von Willebrand factor.

∗Values taken indirectly from graphs and not explicitly described on article's text.

**Table 5:** In vivo characterization studies of VP-printed calcium phosphate-based scaffolds reported in the literature. (d: day/s, w: week/s, m: month/s).

ref	In vivo	Animal model	time
[153]	Orthotopic (forelimb)	New Zealand white rabbits	4, 12 w
[128]	Orthotopic (sheep femur)	Female Sheep (ISO 109936:2016)	6 m
[216]	Orthotopic (skull)	Humans' skull	12 m
[99]	Orthotopic (parietal bone)	New Zealand white rabbits	4, 8 w
[67]	Orthotopic (calvaria defect)	Rabbits, females	0, 3, 6 w
[179]	Orthotopic (mandible)	Adult male beagle dogs	4, 8 w
[180]	Orthotopic (subperiosteal cranial)	Rat	3, 6 m
[108]	Orthotopic (femur)	New Zealand rabbits, male	6, 12 w
[181]	Orthotopic (skull, calvaria)	Rabbit	4, 8 w
[111]	Ectopic (subcutaneous back midline)	SD rats	8 w
[41]	Ectopic (subcutaneous)	Not said	2 w
[107]	Ectopic (dorsal muscles)	New Zealand white rabbits	2 w
[39]	Orthotopic (rat femur)	Rat	4, 8 w
	Ex-ovo (CAM model)	Chicken egg	Embryonic d 10
[192]	Orthotopic (diastolic epiphysis of femoral bone)	White rat (line "Vistar")	2, 4, 8 w
[144]	Ectopic (subcutaneous)	SD male rats	1, 4 w
	Orthotopic (skull)	SD male rats	4, 8 w
[139]	Ectopic (epidermal wounds)	Male New Zealand rabbits	4 w
	Orthotopic (femoral condyle)	Male New Zealand rabbits	4 w
[140]	Orthotopic (femoral condyle)	Male New Zealand rabbits	4, 8 w
	Orthotopic (calvaria)	Rat	1, 2, 3, 6 w
[175]	Orthotopic (distal femur)	New Zealand rabbits	4, 8, 12 w
[186]	Orthotopic (femoral condyle)	Female Bama miniature pigs	3, 6 m
[146]	Orthotopic (femur)	New Zealand rabbits	2, 4 w
[75]	Orthotopic (calvaria)	SD rats	4, 8 w
[74]	Ectopic (intradermal)	Nude mice	30 d
	Orthotopic (cranium)	New Zealand rabbits	4, 12 w
[78]	Ectopic (subcutaneous)	SD rats	1, 7, 14, 21 d
	Orthotopic (calvaria)	SD rats	6 w
[177]	Ectopic (subcutaneous)	Mouse	2, 4 w
[142]	Ectopic (subcutaneous)	SD rats	2, 4 w
	Orthotopic (calvaria)	New Zealand white rabbits	4, 8 w
[171]	Orthotopic (femoral condyle bone)	New Zealand White Rabbit	6, 12 w
[161]	Orthotopic (parietal bones)	Sprague Dawley rats	4 w
[184]	Ectopic (rectus femoris muscle)	BALB/c mouse	60, 90 d
	Orthotopic (femoral condyle)	SD rat	4, 8 w
[162]	Orthotopic (femoral condyle)	Rabbit (New Zealand)	8, 12 w
[85]	Orthotopic (femur diaphysis)	Rat	3, 6 w
[136]	Orthotopic (calvaria)	Rat (male Wistar)	4, 8 w
[185]	Ectopic (rectus femoris muscle)	Mouse (BALB/C)	60, 90 d
[54]	Ectopic (dorsal muscle)	Dog (Beagle)	3 m
[55]	Orthotopic (calvaria defect)	Rat	12 w
[168]	Orthotopic (femoral defect)	Rabbit	4, 8 w
[110]	Orthotopic	Rabbit mandible	4, 8, 12 w
[49]	Orthotopic (femur)	Rabbit	4, 16 w
	Ectopic (dorsal muscles)	Dog (Beagle)	4, 16 w
[166]	Orthotopic (parietal bone)	Rat	8 w
[172]	Orthotopic (femur)	Rabbit	6, 12 w

Furthermore, the following sections summarize the most relevant effects of scaffolds’ composition and their design in the overall biological performance.

### 6.1 Effect of scaffolds composition on biological performance

The composition of the scaffolds directly influences their biological performance when interacting with cells or tissues in animal models. The most commonly reviewed strategies used to modify scaffold composition and enhance biological performance involve either adjusting the inorganic components or the organic polymeric matrix in composite scaffolds before printing or incorporating coatings and/or molecules after the printing process.

**Fig. 8:**
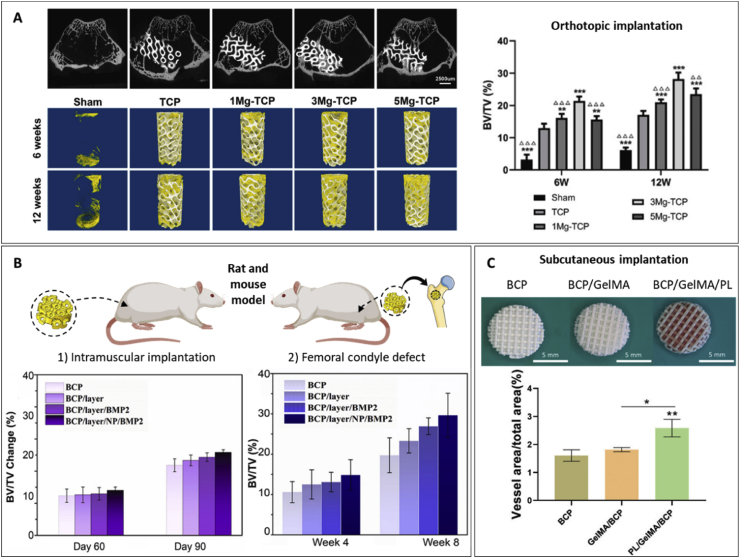
Effect of the scaffold's composition on biological performance; strategies proposed in the literature to improve the biological performance of calcium phosphate scaffolds obtained by VP. (A) Multicomponent printing, by the addition of other components (MgO) to the β-TCP initial powder, Mg-doped scaffolds performed better bone regeneration at orthotopic implantation for both 6 and 12 weeks compared to undoped full ceramic β-TCP counterpart [171]. (B) BMP-2-coated BCP ceramic scaffold performed better bone formation at ectopic and orthotopic implantation for both 2 and 3 month after implantation compared to uncoated BCP scaffolds [184]. (C) The addition of biological elements such as platelet lysates (PL) containing growth factors to a GelMA coating on BCP full ceramic scaffolds enhance the scaffold's vascularization properties in terms of vessel area/total area formation percentage [111].

The first strategy is the modification of the inorganic phase of the slurry, mainly focused on full ceramic scaffolds. The addition of multicomponent inorganic elements to the ceramic slurry has been addressed, as researchers have found that the release of different ions can positively favor bone ingrowth properties. Bivalent cations such as Sr^2+^, Mg^2+^, and Zn^2+^ can replace Ca^2+^ in the crystal structure, altering stability, microstructure, solubility, and ion exchange. These changes positively impact bone formation and material degradation through both chemical effects and lattice modification [217]. Following this line, Qi et al. used MgO as doping agent for full ceramic β-TCP scaffolds, as the appropriate release of Mg^2+^ demonstrated to regulate the immune environment and positively affect osteogenesis [171]. In this study, Mg-doped β-TCP scaffolds with a macroporosity of approximately 67 % and a pore size of 650 μm promoted osteogenesis and angiogenesis through macrophage immunomodulation *in vitro* and *in vivo*. The *in vivo* results reflected around 1.5-fold increase in bone formation (BV/TV) in 3 % Mg doped β-TCP compared to β-TCP controls both in 6 weeks and 12 weeks (Fig. 8A). Alternatively, adding ion-rich crystals like Whitlockite (Ca_9_Mg(PO_4_)_6_(PO_3_OH), [152]), Akermanite (Ca_2_MgSi_2_O_7_, [56,58]), or Bredigite (Ca_7_MgSi_4_O_16_, [145]) also promoted cell proliferation and osteogenic differentiation by releasing Mg^2+^ and Si^4+^. Guo et al. demonstrated that the addition of Bredigite in HA full ceramic scaffolds reduced HA grain size, increased microporosity, and reduced densification, leading to higher porosity, accelerated degradation, and Mg^2+^, and Si^4+^ release. In addition, it also accelerated the release of Ca^2+^ and PO_4_^3−^ from the HA matrix. While reducing the mechanical properties of the scaffolds, the release of Mg^2+^, Si^4+^ Ca^2+^, and PO_4_^3−^, stimulated bone protein production, osteoblast adhesion and growth [145]. Lately, barium titanate (BaTiO_3_) has gained attention in HA/BT doped ceramic scaffolds. During sintering, Ba^2+^ can substitute Ca^2+^, forming Ba_3_(PO_4_)_2_, and CaTiO_3_, promoting cell differentiation [149]. Together with Zn^2+^ substitution, these studies highlight the synergistic effects of piezoelectric properties that support osteogenesis and angiogenesis when combined with low-intensity pulsed ultrasound (LIPUS) [150]. Finally, ZrO_2_, has also been employed to modify the inorganic phase of HA ceramic scaffolds. Gao et al. introduced nano zirconia to coat the HA grains, targeting the inhibition of soft tissue invasion [144]. Zirconia was used for its potential to bind to dectin-1 receptor, known to mediate soft-tissue invasion. As the regeneration of hard and soft tissue is competitive and interactive, this study aimed at potentiating osteogenesis while reducing soft tissue generation. Although not inducing bone regeneration subcutaneously, HA/zirconia scaffolds caused an anti-inflammatory environment with little fibrosis after 4 weeks. Besides, when implanted in calvaria it resulted in decreased inflammation and soft tissue invasion, and accelerated bone regeneration at 4 weeks, showing around two-fold increased BV/TV in HA/nano zirconia group compared to HA control group.

Conversely, other researchers have focused on the modification of the organic part of polymer/CaP composite scaffolds made prior to printing, by formulating polymeric resins that contain biomolecules. Modified polymers are often envisioned to improve local cell-material interactions, providing textural or biologically engineered surfaces where cells can anchor through integrins in their membrane. Zhou et al. mixed GelMA with PEGDA to print HA-containing composite scaffolds, as GelMA exhibits notable biocompatibility due to the abundant arginine-glycine-aspartic acids (RGD) and matrix metalloproteinase (MMP) poly-peptide sequences, which can significantly promote cell attachment and proliferation while maintaining an adequate strength of the material using PEGDA [70,71]. In addition, in other studies, they substituted GelMA by incorporating RGD peptidic sequences directly into stronger synthetic PEGDA polymers. In fact, they demonstrated enhanced differentiation of mesenchymal stem cells (MSC) through alkaline phosphatase activity (ALP) assays on PEGDA-nHA composite scaffolds. Incorporating RGD sequences the ALP activity increased from 1.9 μmol/(L min) to 2.1 μmol/(L min) at week three. This effect was further amplified by treating the scaffold with low-intensity pulsed ultrasound (LIPUS), raising the ALP activity to approximately 2.3 μmol/(L min) at week three [196]. Moreover, Liu et al. highlighted the reduced list of natural polymers for printing bioactive scaffolds, and especially for bioprinting with cells, where GelMA is the most used polymer [76]. Their study focused on the synergistic effects of using methacrylated bone-derived decellularized extracellular matrix (bdECM-MA) and silicon-substituted CaP (Si-CaP) to bioprint bone-derived mesenchymal stem cells (BMSCs). *In vitro*, both materials enhanced osteogenesis and angiogenesis, with bdECM-MA promoting collagen secretion and endothelial growth, while Si-CaP boosting ALP expression. Combined, they showed synergistic effects and activated the TLR4–PI3K–AKT pathway. They also modulated the immune response by inhibiting the p38-MAPK pathway and promoting anti-inflammatory macrophage polarization. *In vivo*, the composite supported sequential immune modulation, enhanced vascularization, and achieved near-complete bone regeneration within 6 weeks. The findings highlighted their strong therapeutic potential and encourage further study of the immunomodulatory mechanisms.

Finally, coatings and functionalizing molecules can be included as part of the scaffold after printing. Tang et al. used Bone Morphogenetic protein-2 (BMP-2), a bone growth factor used to induce osteoblastic differentiation, by incorporating it into nanogels made of Heparin and Polyethyleneimine (PEI), which have good biocompatibility, water solubility, and biodegradation, and applying it as coating for BCP full ceramic scaffolds. Their findings revealed not only a promotion of BMSC proliferation, migration, and osteoblastic differentiation *in vitro* but also ectopic bone formation and accelerated bone regeneration *in vivo*. These results indicated a slightly increased bone formation fraction (BV/TV) for ectopic implantation for BMP-2 coated BCP ceramic scaffold compared to uncoated BCP scaffolds, showcasing the osteoinductive properties of morphogenic proteins. The most significant effect was revealed in orthotopic implantation, where BMP-2 coated BCP scaffolds resulted in a 10 % increase in bone formation after 8 weeks compared to the BCP control (Fig. 8B) [184]. In a similar approach but focusing on vascularization, Liu et al. coated a BCP full ceramic scaffold with platelet lysate (PL)-rich GelMA coating, which promoted angiogenesis [111]. Their findings revealed a significant increase in blood perfusion, number of capillaries, and vessel areas ratio with over 1.5 % improvement for PL-GelMA coated BCP scaffold compared to the uncoated control when implanted subcutaneously in the back of rat models (Fig. 8C). Finally, the integration of traditional pharmaceuticals to CaP scaffolds is gaining attention [139]. Gui et al. incorporated icariin, a primary chemical constituent extracted from plants (*Epimedium genus* in the *Burseraceae* family) in GelMA hydrogels to coat HA full-ceramic scaffolds after printing. Icariin has the promising ability to enhance stem cell osteogenic differentiation and vascular regeneration. The incorporation of icariin promoted angiogenesis and osteogenesis, outperforming the HA full ceramic groups both *in vitro* and *in vivo*. The HA/GelMA/Icariin group promoted the activation, proliferation, migration, and tube formation ability of HUVECs, and promoted the differentiation of rabbit BMSCs *in vitro*. Furthermore, *in vivo* tests concluded that icariin-loaded scaffolds promoted osteoinduction after 4 weeks of subcutaneous implantation showing ∼4 mm^3^ of new bone formation compared to ∼2 mm^3^ in the icariin-free scaffolds [139]. Furthermore, the HA/GelMA/icariin scaffolds showed a relative in-growth bone area of 9 %, compared to 5.5 % in icariin-free HA scaffolds, orthotopically after 8 weeks [140].

### 6.2 Effect of scaffolds design on biological performance

**Fig. 9:**
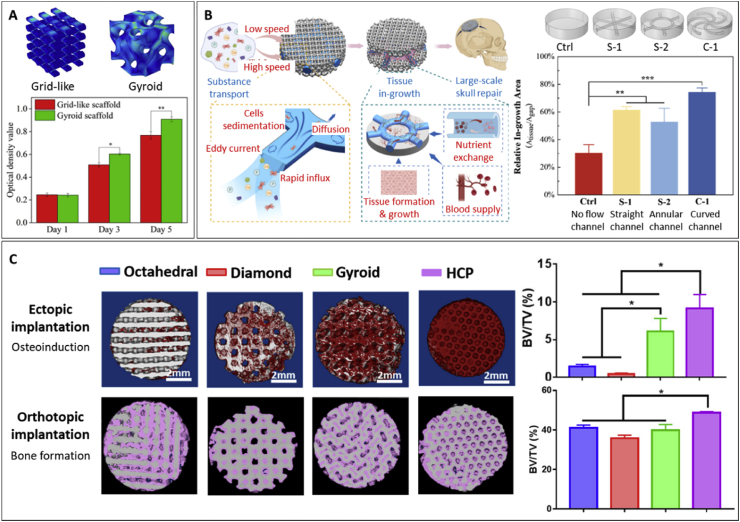
Effect of the scaffold's architecture on biological performance; strategies proposed in the literature to improve the biological performance of calcium phosphate scaffolds obtained by VP. (A) β-TCP full ceramic scaffolds with similar porosity for gyroid and grid-like geometries show different proliferation results of mouse bone marrow stem cells (mBMSCs) at day five. Gyroid-TPMS geometry showed a higher proliferation compared to common grid-like counterpart [167]. (B) flow-channel designs promote bone ingrowth and vascularization in ectopic implantation by facilitating a rapid infiltration of the HA full ceramic scaffold's inner struts and the smooth transportation of substances, forming a richer metabolic microenvironment [107]. (C) Hexagonal close-packed (HCP) BCP full ceramic structures promoted osteoinductivity, with enhanced new bone formation after 10 weeks *in vivo* ectopic implantation, and slightly promoted bone formation in orthotopic implantation after 8 weeks [215].

Similar to the mechanical performance outcomes, the overall scaffold architecture greatly affects their biological outcome. Scaffolds' designs, often dictating pore geometry, total porosity, and interconnectivity influence the cellular response, bone ingrowth and resorption rates, crucial for bone remodeling. For instance, studies have shown that smooth, curved geometries like TPMS promoted higher mechanical strength and cell proliferation than commonly designed grid-like structures in extrusion-based techniques [167]. Using VP printed β-TCP full ceramic scaffolds with a porosity of 60 % and 300 μm pore size for both geometries, *in vitro* studies of mouse bone marrow stem cells (mBMSCs) at day five showed a higher proliferation for gyroid compared to common grid-like counterpart (Fig. 9A). Among the various TPMS geometries, gyroid stands as one of the most promising. Recent research has demonstrated that gyroid structures promote greater bone formation *in vivo* compared to that of Diamond or Schwarz primitive [218]. Additionally, the influence of pore geometry on enhancing and steering cell activity and bone regeneration has garnered great interest [215]. Werner et al. showed that human bone marrow stem cells (hBMSCs) exhibited faster migration on concave surfaces compared to convex ones [219]. In line with this, previous studies using strut-based printed scaffolds have highlighted the need for concave surfaces within the scaffolds to enhance bone regeneration *in vivo* [220,221]. Biologically, the need for concavities capable of concentrating important ions, growth factors and proteins has been long accepted [7].

Generally, a greater bone formation is obtained with rapid cell colonization and proliferation, resulting from proper nutrient and cell diffusion throughout the printed scaffold. Therefore, different studies have focused on the promotion of permeability/flowability of the scaffolds following different strategies. Mao et al. designed interpenetrating flow channels in HA scaffolds, which appeared to be helpful for the rapid infiltration of the scaffold's inner struts and the smooth transportation of substances, forming a richer metabolic microenvironment for tissue regeneration by bringing richer nutrients and targeting cells. In fact, the flow channels revealed a higher relative bone ingrowth area than the conventional scaffold counterpart implanted *in vivo* in rabbit muscle (ectopic implantation) [107]. The results showed a prominent increase when comparing a control group without channels, with a relative in-growth area of ∼30 %, with scaffolds with channels, showing ∼50–70 % in-growth area (Fig. 9B). Following this idea, Gui et al. fostered bone regeneration by creating internal channels, mimicking the structure of loofah sponges. The enhanced flowability of the loofah-like scaffolds, combined with a drug-loaded coating, created a synergistic effect showing an optimum drug distribution inside the scaffold that promoted bone regeneration capability both *in vitro* and *in vivo* [139]. Regarding water penetration, Lee et al. found that elongated pores in β-TCP full ceramic scaffolds showed also much faster water penetration [113]. This discovery led them to suggest that porous CaP scaffolds with elongated pores would enhance the transport of blood, oxygen, and nutrients, thereby promoting faster bone regeneration. However, the search for better flowability properties comes with a decrease in mechanical properties. Therefore, a balance between these two properties must be considered when designing bone grafts.

Despite the decrease in mechanical properties, permeability can strongly influence the scaffold's properties *in vitro* and *in vivo*. Wu et al. tested BCP full ceramic scaffolds with varying pore architecture, namely gyroid, octahedral, diamond, and hexagonal close-packed (HCP), with comparable macroporosity and pore size (∼70 % and ∼515 μm respectively). They reported that compared to the other architectures, hexagonal close-packed (HCP) exhibited 1) a more uniformly cell growth along the pore walls, 2) significantly higher osteogenic genes and proteins expressions *in vitro* using MC3T3-E1, 3) higher osteoinductivity related to an enhanced new bone formation on an ectopic *in vivo* model after 10-week, with up to eight times greater bone formation (BV/TV) than octahedral (with an 8.02 ± 1.94 % of new bone), 4) higher new bone formation on orthotopic implantation, showing slightly higher bone formation capability for HCP with approximately around 10 % higher new bone (BV/TV) compared to orthogonal geometry after 8 weeks (Fig. 9C), 5) upregulated expression of angiogenic factors, and 6) high bioactivity regarding a full coverage of the surface with precipitated bone-like apatite after SBF immersion [215]. The role of pore geometry in bone regeneration was attributed to the round and close-packed nature of HCP pores, which diminishes permeability with a lower liquid flow rate as obtained with simulation and mercury porosimetry. This control in flow rate is thought to create a more stable environment, promoting the formation of bone-like apatite and increasing the biological performance. This is consistent with previous reports, which indicate that bone formation typically occurs in concavities rather than convexities, suggesting that concave surfaces are more effective at accumulating calcium and phosphate ions compared to convex pores [7].

## 7 Conclusions and future perspectives

In regenerative medicine, computer-aided three-dimensional printing stands as one of the most promising methods for fabricating bone implants. These additive manufacturing techniques offer high versatility and precision, enabling the creation of patient-specific grafts tailored to individual trauma needs. Notably, Vat photopolymerization (VP) printing has gained increasing attention in the field of bone regeneration. This review highlights the significant potential of calcium phosphate (CaP)-based scaffolds, which closely mimic the mineral phase of natural bone and possess osteoconductive and osteoinductive properties. The development of CaP scaffolds has rapidly advanced, with a clear trend towards using high-resolution AM techniques such as VP, which provides distinct advantages regarding other techniques in promoting bone healing.

The use of ceramic loaded resins in VP involves a suspension of CaP particles that must meet rigorous optical and rheological requirements. Numerous researchers have addressed this challenge, optimizing the printability of slurries containing up to 70 % ceramic in weight. Advances in understanding the interactions between slurry components have led to the development of complex compositions, such as introducing doping agents, biopolymers, and even therapeutic components, hardly considered in other AM techniques. Ongoing efforts focus on improving printing accuracy, maintaining design integrity, enhancing slurry printability, and evaluating the mechanical and biological performance of scaffolds.

VP offers the possibility of using a wide range of materials to create CaP-based bone scaffolds. In fact, resins can be extensively formulated with different photocurable polymers that provide varying characteristics. When printing with calcium phosphates, the resulting composite structures can follow two distinct processing routes, each affecting the microstructure. These routes lead either to a full ceramic body or a polymeric composite framework reinforced with ceramic particles.

Around 75 % of the reviewed studies are focused on full-ceramic scaffolds and the optimization of the thermal treatments after the printing process. Typically, full-ceramic scaffolds include high-temperature CaPs such as hydroxyapatite and beta-tricalcium phosphate, resulting in a sintered-grain microstructure. One key challenge when fabricating full-ceramic scaffolds is the inevitable shrinkage during thermal treatment and subsequent dimensional reduction, resulting in alteration of the initial CAD design and imposing rigorous dimensional characterization. In terms of mechanical properties, although sintered ceramic scaffolds have high compressive strength, they are brittle materials, which can be aggravated by the presence of cracks, abnormal grain growth, poor densification, phase transformations, and other microstructural defects. Furthermore, while showing excellent biocompatibility, sintered CaP scaffolds and especially HA have limited bone-forming capacity due to their reduced surface area and degradability. Some strategies to overcome this limitation include the incorporation of doping agents to enhance reactivity by distorting the crystal lattice, the combination with more soluble second phases, or the increase of microporosity. All of them increase ion release, able to interact with the surrounding cells, aiming at enhancing their biological activity, but in turn may compromise mechanical stability. To preserve the balance between mechanical properties and biological performance, another approach is the introduction of therapeutic-loaded biopolymers onto CaP scaffolds.

In contrast, composite scaffolds are based on biocompatible resins composed of cell-friendly monomers/oligomers, and photoinitiators, combined with CaP particles to form a composite structure. This usually results in a continuous polymeric matrix with dispersed ceramic particles, which resembles more closely the composition of natural bone. The combination of polymers and ceramics provides increased ductility and toughness while maintaining suitable compressive strength. Moreover, as no thermal treatment is required, dimensional changes from the original CAD designs are reduced or eliminated. Synthetic biocompatible polymers such as PEGDA, PTMC-MA, and HDDA, among others, confer adequate rigidity and toughness to the composite structure. However, their inert nature, lacking binding sites for cell adhesion, limits their bioactivity. Natural biopolymers derived from gelatin, silk fibroin, or bone-derived extracellular matrix can be functionalized with acrylate terminating groups, creating hydrogels that can be incorporated as the matrix of CaP composites instead of using them as coatings. In this way, osteogenesis and angiogenesis are promoted due to the increased cell-binding sites and biodegradation. In addition, the use of biopolymers may support bioprinting, with the incorporation of cells inside the structure further enhancing bone regeneration. However, despite the great potential of this family of materials, the list of possible candidates is still very narrow, and the development of new formulations of photosensitive biopolymers is emerging as one of the lines of progress for VP-printing of composite CaPs.

Another remaining challenge is to increase the CaP loading in biopolymer composite resins, as current studies use low amounts, with materials that lack sufficient mechanical properties, making manipulation, implantation, and stability in the host tissue challenging. Furthermore, the possibility of using new types of CaPs may open the way to materials with novel properties. In this regard, investigating the potential of using calcium phosphate cements (CPCs), such as α-TCP, could lead to the formation of a continuous ceramic network following the hardening reaction. This network can have the capacity to interpenetrate the solidified polymeric resin.

One critical feature that has been widely highlighted in this review is the role of the scaffold's pore architecture, which has revealed important outcomes in terms of mechanical stability and biological performance. Advanced geometries containing smooth curvatures, including Triply Periodic Minimal Surfaces (TPMS), have gained important attention, demonstrating increased mechanical stability by alleviating stress concentrations. Pore curvature is also responsible for creating an adequate environment for directing cell growth *in vitro* and bone regeneration *in vivo*. The chosen architecture and porosity also affect the scaffold's permeability, and in doing so, affect the biological response. In fact, an interconnected structure with adequate permeability is desired, permitting a proper interconnection between newly formed blood vessels, nutrients and growth factors, as well as the scaffold's degradation. However, the macroporous nature also affects the mechanical stability. Therefore, it is imperative to balance properties such as porosity, pore size, and geometrical design parameters together with the material and processing strategy selection.

One of the great advantages of VP is that it has high resolution and is capable of creating intricate structures, otherwise very difficult to replicate using other AM techniques. SLA and DLP (using DMD or LCD) are the only explored techniques to fabricate CaP scaffolds. While demonstrating exceptional resolution and the ability to create complex, curvature-rich-structures, these techniques operate in layered configurations, which lead to the so-called “stair-stepping” effect, referring to the individual layers stacked along the printing direction. This leads to certain degree of mechanical anisotropy. Although higher-resolution VP techniques, such as Continuous Liquid Interface (CLIP) and Two-Photon Polymerization (2PP) have not yet been explored, their implementation to fabricate CaP scaffolds could lead to smoother surfaces, higher isotropy, and enhanced personalization of medical implants. However, it is crucial to consider that higher resolution increases the printing time, which could jeopardize the stability of the resin during the process.

Infection risks remain a critical concern during scaffold implantation. To date, limited research has addressed this issue, with most efforts relying on antibiotic-based approaches. As pointed by the World Health Organization, antibiotic-resistant implant-related infections present a major challenge in biomedical devices, prompting diversification of treatments alternative to antibiotic-based ones. Promising strategies such as nature-inspired nano-topographies, with bactericidal properties, or antimicrobial peptides (AMPs), such as lactoferrin, have recently attracted scientific attention [222,223]. Nature-inspired nano-topographies can be replicated in CaP ceramics, where features such as crystal aspect ratio significantly influence antimicrobial activity. Acicular CDHA nanocrystals, characterized by a high aspect ratio, have demonstrated bactericidal potential [224], though this remains unexplored in macroporous 3D-printed scaffolds. Additionally, AMP functionalization of printed scaffolds, or their incorporation in the resin formulation could further offer antibacterial strategies [225,226]. Future research should continue to explore the integration of antibacterial properties, whether through mechanical design or compositional adjustments.

In conclusion, VP, combined with calcium phosphate materials and advanced pore designs, offers a powerful approach to improving bone scaffolds compared to existing additive manufacturing procedures, balancing mechanical stability and biological performance. Looking ahead, this technology holds great promise for addressing future challenges in bone tissue engineering. Its high resolution is ideally suited for creating intricate, scaffolds that mimic the complex microenvironment of native bone, paving the way for advanced *in vitro* models and ultimately, functional tissue regeneration. Furthermore, the versatility of VP allows for the incorporation of therapeutic agents directly into the scaffold material, offering a potent strategy to develop bone scaffolds that can release therapeutic agents in a controlled manner, offering unprecedented opportunities for personalized and dynamic bone regeneration therapies.

## CRediT authorship contribution statement

**Roberto Fagotto-Clavijo:** Writing – original draft, Visualization, Investigation, Formal analysis, Data curation, Conceptualization. **Irene Lodoso-Torrecilla:** Writing – review & editing, Investigation, Data curation, Conceptualization. **Anna Diez-Escudero:** Writing – review & editing, Supervision, Investigation, Data curation, Conceptualization. **Maria-Pau Ginebra:** Writing – review & editing, Supervision, Funding acquisition, Conceptualization.

## Ethics approval and consent to participate

Not applicable for this manuscript. This study did not involve animal/human participants, animal/human data, or animal/human tissue.

## Declaration of competing interest

The authors declare no competing nor financial interests, or personal relationship that influenced this paper.
