# Supplementary material for: Strategic advances in Vat Photopolymerization for 3D printing of calcium phosphate-based bone scaffolds: A review
Source: Bioact Mater. 2025 Jun 27;52:719–52. doi: 10.1016/j.bioactmat.2025.05.001 (PMC12269438; doi:10.1016/j.bioactmat.2025.05.001)
Supplement: Multimedia component 1 [file mmc1.docx]

Supplementary information

**Strategic advances in Vat Photopolymerization for 3D printing of calcium phosphate-based bone scaffolds: A Review**

Roberto Fagotto-Clavijo^a,b,c^, Irene Lodoso-Torrecilla^a,b^, Anna Diez-Escudero *^a,b,c^, and Maria-Pau Ginebra **^a,b,c,d^

^a^Biomaterials, Biomechanics and Tissue Engineering (BBT), Department of Materials Science and Engineering, Universitat Politècnica de Catalunya (UPC) and Institute for Research and Innovation in Health (IRIS), Av. Eduard Maristany, 16, Barcelona 08019, Spain.

^b^Barcelona Research Centre in Multiscale Science and Engineering, Universitat Politècnica de Catalunya (UPC), Av. Eduard Maristany, 16, Barcelona 08019, Spain.

^c^CIBER de Bioingeniería, Biomateriales y Nanomedicina (CIBER-BBN), Instituto de Salud Carlos III, Spain.

^d^Institute for Bioengineering of Catalonia (IBEC), The Barcelona Institute of Science and Technology, Baldiri Reixac 10-12, 08028 Barcelona, Spain

*Corresponding author. Present address: Department of Materials Science and Engineering, Universitat Politècnica de Catalunya (UPC), Av. Eduard Maristany, 16, 08019 Barcelona, Spain. E-mail addresses: anna.diez-escudero@upc.edu (A. D.-Escudero).

**Corresponding author. Present address: Department of Materials Science and Engineering, Universitat Politècnica de Catalunya (UPC), Av. Eduard Maristany, 16, 08019 Barcelona, Spain. E-mail addresses: maria.pau.ginebra@upc.edu (M.-P. Ginebra).

# Abbreviations

Table S1: List of abbreviations used in figures and tables throughout the review.

| Source | Acronyms | Meaning |
| --- | --- | --- |
| Figure 2 | α-CS | α-Calcium silicate |
|  | AK | Akermanite |
|  | BCP | Biphasic Calcium Phosphate |
|  | BG | Bioglass |
|  | BR | Bregidite |
|  | BT | Barium titanate |
|  | CPP | Calcium pyrophosphate |
|  | HA | Hydroxyapatite |
|  | MAEP | Monoalcohol ethoxylate phosphate |
|  | MCPM | Mono-calcium phosphate monohydrate |
|  | OCP | Octacalcium phosphate |
|  | Si-CaP | Silicon-Calcium phosphate |
|  | SWCNT | Single-walled carbon nanotube |
|  | TCP | Tricalcium phosphate |
| Table 2 | α-β-TCP | α-β-Tricalcium phosphate |
|  | β-CEA | β-carboxyethyl acrylate |
|  | ACMO | Acryloylmorpholin (4-(1-oxo-2-propenyl)-morpholine) |
|  | AK | Akermanite |
|  | AM | Acrylamide |
|  | BAPO | phenylbis(2,4,6-trimethyl-benzoyl)-phosphineoxide |
|  | BCP | biphasic calcium phosphate |
|  | BG | Bioglass |
|  | CEA | β-carboxyethyl acrylates |
|  | CQ | Camphorquinone |
|  | EDMD | ethanone, 2,2-dimethoxy-1,2-diphenyl |
|  | HA | Hydroxyapatite |
|  | HA-DA | hyaluronic acid-dopamine |
|  | HDDA | 1,6-hexanediol diacrylate |
|  | HEMA | 2-hydroxyethyl methacrylate |
|  | IBOA | isobornyl acrylate |
|  | MAEP | monoalcohol ethoxylate phosphate |
|  | MBAM | N-N′ methylenebisacrylamide |
|  | MeHQ | P-hydroxyanisole |
|  | OA | Oleic acid |
|  | OPPEA | 2-([1,1′-biphenyl]- 2-yloxy) ethylacrylate |
|  | PEG | Poly(ethylene glycol) |
|  | PEGDA | Poly(ethylene glycol) diacrylate |
|  | PMMA | Poly(methyl methacrylate) |
|  | PPG | Polypropylene glycol |
|  | PPO | phenylbis (2,4,6-trimethylbenzoyl) phosphine oxide |
|  | SPA | sodium polyacrylate |
|  | SPMA | sodium polymethacryate |
|  | TAEA | Tris(2-Hydroxyethyl) Amine |
|  | TGDA | tetraethylene glycol diacrylate |
|  | TMP3EOTA | ethoxylated trimethylolpropane triacrylate |
|  | TMPTA | trimethylol- propane triacrylate |
|  | TPGDA | tripropylene glycol diacrylate |
|  | TPO | diphenyl(2,4,6-trimethylbenzoyl)phosphine oxide |
|  | TPO-L | ethyl phenyl(2,4,6-trimethylbenzoyl)phosphinate |
|  | TTA | trimethylolpropane trimethacrylate |
|  | UA | Urethane acrylate |
|  | UDMA | diurethane dimethacrylate |
| Table 3 | α-β-TCP | α-β-Tricalcium phosphate |
|  | AESO | acrylated epoxidized soybean oil |
|  | AlgMA | alginate methacrylate |
|  | BAPO | phenylbis(2,4,6-trimethyl-benzoyl)-phosphine oxide |
|  | BCP | biphasic calcium phosphate |
|  | CSMA-2 | r((3R, 3aR, 6S, 6aR)-hexahydrofuro [3,2-b] furan-3,6-diyl) bis(oxy)) bis(ethane-2,1-diyl)) bis(oxy)) bis(carbonyl)) bis(azanediyl)) bis(3,3,5-trimethylcyclohexane-5,1-diyl)) bis(azanediyl)) bis(carbonyl))bis(oxy)) bis(ethane-2,1-diyl) bis(2-methylacrylate)) |
|  | CPP | calcium pyrophosphate |
|  | CQ | camphorquinone |
|  | DEF | diethyl fumarate |
|  | E104 | Quinoline Yellow food coloring |
|  | ETTMP | ethoxylated trimethylolpropane tri(3-mercaptopropionate) |
|  | GelMA | Gelatin-methacrylate |
|  | HA | hydroxyapatite |
|  | MCPM | Mono-Calcium Phosphate Monohydrate |
|  | NMP | N-Methyl-2-pyrrolidone |
|  | NPs | Nanoparticles |
|  | OCP | octacalcium phosphate |
|  | OCM-2P | olygocarbonate dimethacrylate |
|  | OlMA | methacrylated oligolactide |
|  | PAA | polyacrylic acid |
|  | PBS | phosphate-buffered saline |
|  | PDLLA | Poly(DL-lactide) |
|  | PEG | Poly(ethylene glycol) |
|  | PEGDA | Poly(ethylene glycol) diacrylate |
|  | PEGMA | Polyethylene glycol monomethacrylate |
|  | PETMP | pentaerythritol tetrakis(3-mercaptopropionate) |
|  | PLA-MA | Polylactic acid-methacrylate |
|  | PLGA | poly(lactic-co-glycolic) acid |
|  | PLLA | Poly(L-lactide) |
|  | PPF | Poly(propylene fumarate) |
|  | PTMC-MA | Poly(trimethylene carbonate)-methacrylate |
|  | PYG | pyrogallol |
|  | RGD | Arginine-glycine-aspartic acid peptide sequence |
|  | Si-CaP | Silicon-calcium phosphate |
|  | SilMA | silk fibroin methacrylate |
|  | SWCNT | single-walled carbon nanotubes |
|  | TATO alkene | 1,3,5-tiallyl-1,3,5-triazine-2,4,6-trione |
|  | TATO thiol | tris[2-(3-mercaptopropionyloxy)ethyl]-isocyanurate |
|  | TEA | triethanolamine |
|  | TEGDMA | triethylene glycol dimethacrylate |
|  | TGFb1 | Transforming Growth Factor Beta 1 |
|  | TMPMP | trimethylolpropane tris(3-mercaptopropionate) |
|  | TMPTMA | Trimethylolpropane trimethacrylate |
|  | TPO | diphenyl(2,4,6-trimethylbenzoyl) phosphine oxide |
|  | TPO-L | ethyl phenyl(2,4,6-trimethylbenzoyl)phosphinate |
| Tables 4 and 5 | ADSC | human adipose stem cell |
|  | ALP | Alkaline phosphatase |
|  | ARS | alizarin red S |
|  | BMP-2 | bone morphogenetic protein-2 |
|  | BMSC | Bone marrow stem cells |
|  | BrCa | Breast cancer |
|  | CAM/PI | Calcein-AM and Propidium Iodide solutions |
|  | CCK8 | cell counting kit 8 |
|  | CD31 | cluster of differentiation 31 |
|  | COL1 | collagen type 1 |
|  | DAPI | 2-(4-amidinophenyl)-1H -indole-6-carboxamidine |
|  | EPC | endothelial progenitor cells |
|  | GAG | Glycosaminoglycans |
|  | GAPDH | glyceraldehyde-3-phosphate dehydrogenase |
|  | hBMSC | human bone marrow stem cells |
|  | hDF | Human Dermal Fibroblasts |
|  | hFOB | human foetal osteoblast |
|  | hMSC | human mesenchymal stem cells |
|  | hTMSC | Human turbinate mesenchymal stromal cells |
|  | HUVEC | human umbilical vein endothelial cells |
|  | IL-8 | Interleukin-8 |
|  | MAPK | mitogen activated protein kinase |
|  | mBMSC | mouse bone marrow stem cell |
|  | MC3T3-E1 | mouse calvaria cell line |
|  | MCF-7 | Breast cancer cell line |
|  | MDA-MB.231 | breast epithelial cell line |
|  | MG-63 | Osteosarcoma derived cell line |
|  | MTS, MTT, XTT | cell viability assays |
|  | NCTC-L929 | NCTC clone 929 Clone of strain L |
|  | OCN | osteocalcin |
|  | OPN | osteopontin |
|  | PBS | Phosphate buffered saline |
|  | pNPP | nitrophenylphosphate |
|  | RAW264,7 | macrophage cell line |
|  | rBMSC | rabbit bone marrow derived mesenchymal stem cell |
|  | RT-qPCR | real-time quantitative Polimerase chain reaction |
|  | Runx-2 | runt-related transcription factor 2 |
|  | SBF | simulated body fluid |
|  | SD rats | SPRAGUE DAWLEY® |
|  | SEM | scanning electron microscopy |
|  | Sp7 | osterix |
|  | Spp1 | osteopontin |
|  | TRIS-HCl | 2-Amino-2-hydroxymethyl-propane-1,3-diol |
|  | U2OS | human osteo- sarcoma cell line |
|  | VEGF | Vascular endothelial growth factor |
|  | VEGFR2 | Vascular endothelial growth factor receptor 2 |
|  | vWF | von Willebrand factor |

# Methodology for the bibliographic search strategy

The review was conducted through a bibliographic search using three databases (Scopus, Web of Science, and PubMed) focusing on the fields described below. The bibliographic search conducted expands to March 2025.

(TS=(digital light processing) OR TS=(DLP) OR TS=(vat photopolymerization) OR TS=(stereolithography) OR TS=(SLA) OR TS=(LCD) OR TS=(Liquid crystal display)) AND (TS=(CALCIUM PHOSPHATE*) OR TS=(HYDROXYAPATIT*) OR TS=(TRICALCIUM PHOSPHATE*)) AND (TS=(BONE))

And PubMed with the following searching fields:

("Bone regeneration"[Mesh] OR "Osteogenesis"[Mesh] OR "Fracture healing"[Mesh] OR (Bone regeneration[Tiab]) OR ((Bone[Tiab]) AND (regeneration[Tiab])) OR Osteogenesis[Tiab] OR (Fracture healing[Tiab]) OR ((Fracture[Tiab]) AND (Healing[Tiab])) (Bone Formation[Tiab]) OR ((Bone[Tiab]) AND (Formation[Tiab])) OR (Bone repair[Tiab]) OR ((Bone[Tiab]) AND (Repair[Tiab])) OR (Bone healing[Tiab]) OR ((Bone[Tiab]) AND (Healing[Tiab])) OR (new bone[Tiab]) OR (new formed bone[Tiab]) OR (newly formed bone[Tiab]) OR (bone forming[Tiab]) OR (formation of bone[Tiab]) OR (formation of new bone[Tiab])) AND ("Bone Substitutes"[Mesh] OR "Bone Cements"[Mesh] OR "Bone Transplantation"[Mesh] OR "Calcium phosphates"[Mesh] OR Beta-tricalcium Phosphate[Supplementary Concept] OR Alpha-tricalcium Phosphate[Supplementary Concept] OR Tricalcium Phosphate[Supplementary Concept] OR Bone substitute*[Tiab] OR ((Bone[Tiab] OR Bones[Tiab]) AND (Substitute[Tiab] OR Substitutes[Tiab])) OR ((Artificial[Tiab] OR Artificials[Tiab]) AND (Bone[Tiab] OR Bones[Tiab])) OR ((Bone[Tiab] OR Bones [Tiab]) AND (Replacement[Tiab] OR Replacements[Tiab])) OR (Calcium[Tiab] AND Phosphate[Tiab]) OR Calcium phosphate*[Tiab] OR CPC[Tiab] OR CPCs[Tiab] OR ((Bone[Tiab] OR Bones[Tiab]) AND (Cement[Tiab] OR Cements[Tiab])) OR Tricalcium phosphate[Tiab] OR Tricalcium phosphates[Tiab] OR Tricalcium orthophosphate[Tiab] OR Tricalcium Diphosphate[Tiab] OR Tricalcium Phosphate Ceramic[Tiab] OR Beta-tricalcium Phosphate[Tiab] OR Calcium Superphosphate[Tiab] OR a-TCP[Tiab] OR b-TCP[Tiab] OR α-TCP[Tiab] OR β-TCP[Tiab] OR alpha-TCP[Tiab] OR beta-TCP[Tiab] OR alpha-tricalcium[Tiab] OR beta-tricalcium[Tiab] OR tetracalcium phosphate[Tiab] OR TTCP[Tiab] OR Dicalcium Phosphate[Tiab] OR DCPA[Tiab] OR hydroxyapatite*[Tiab] OR Synthetic bone[Tiab]) AND (Vat-polymerization[Tiab] OR Vat polymerization[Tiab] OR Vat-photopolymerization [Tiab] OR Vat photopolymerization [Tiab] OR Two-photon lithography[Tiab] OR TPL[Tiab] OR stereolithography[Tiab] OR SLA[Tiab] OR single-photon lasers[Tiab] OR digital light processing[Tiab] OR DLP[Tiab] OR volumetric bioprinting[Tiab] OR volumetric printing[Tiab] OR volumetric additive manufacturing[Tiab] OR VAM[Tiab])

# Bibliometrics on Vat photopolymerization printing of calcium phosphates for bone regeneration

Figure S1: Bibliometric bar chart representing the yearly publications reviewed, until March 2025, related to Vat photopolymerization printing with calcium phosphates for bone regeneration.

# Mechanical performance of VP-printed calcium phosphate-based scaffolds

Table S2: Mechanical properties of VP-printed calcium phosphate-based scaffolds categorized according to their macroporosity, geometry, and material used to manufacture following either low-temperature route, or high-temperature route. TPMS geometries are solid otherwise mentioned as sheet-TPMS. Values with an asterisk (*) represent not-reported data, extracted directly from graphs.

| **Ref.** | **Geometry** | **Material composition** | **Porosity (%)** | **Compressive strength (MPa)** | **Compressive modulus (MPa)** | **Flexural strength (MPa)** | **Flexural modulus (MPa)** | **Strain to failure (%)** |  |
| --- | --- | --- | --- | --- | --- | --- | --- | --- | --- |
| **Composite scaffolds** | | | | | | | | | |
| [1] | Inverse orthogonal (squared pores) | HA + SWCNT + Dental resin, DITAX | 55 | 25 |  |  |  |  |  |
|  |  |  | 57 | 33 |  |  |  |  |  |
|  |  |  | 59 | 36 |  |  |  |  |  |
| [2] | Solid-Gyroid | BCP + PLLA + TMPTA | 70.2 | 3.63 | 51.2 |  |  |  |  |
|  |  |  | 56.8 | 1.61 | 34 |  |  |  |  |
|  |  |  | 51.8 | 0.69 | 15.5 |  |  |  |  |
| [3] | Orthogonal | HA + methacrylated oligolactide + triethylene glycol dimethacrylate | 38.85 | 1.58 |  |  |  |  |  |
|  |  |  | 43.89 | 0.25 |  |  |  |  |  |
|  |  |  | 43.99 | 0.08* |  |  |  |  |  |
| [4] | Solid-Gyroid | Si-CaP + PEGDA | 60.6 | 2.096 |  |  |  |  |  |
| [5] | Sheet-Gyroid | BCP + Commercial resin Portux Print 3D Model | 80 | 40 | 400 |  |  | 13 |  |
| [6] | Solid-Gyroid | α-TCP → (CDHA) + Epoxi | 36.7 | 16.3 |  |  |  | 12 |  |
|  | Solid-Diamond |  | 30.2 | 16.1 |  |  |  |  |  |
|  | Solid-Schwarz |  | 31.5 | 34.9 |  |  |  |  |  |
| [7] | Double gyroid | HA + PDLLA | 78.16 | 0.68 ± 0.2 | 12.27 |  |  | 7 |  |
|  |  |  |  | 1.23 ± 0.2 | 30.76 |  |  | 9 |  |
| [8] | Solid-Gyroid | 5% OCP | 70 | 0.047 |  |  |  |  |  |
|  |  | 5% Brushite | 70 | 0.079 |  |  |  |  |  |
|  |  | 10% Brushite  + PEGDA | 70 | 0.171 |  |  |  |  |  |
| [9] | Orthogonal | 10 β-TCP + 90 PLA | 48.3 | 52.1 |  |  |  | 31.4 |  |
| [10] | Orthogonal | 10bdECM+0.5Si-CaP | 68.83 | 0.22* | 1.42 ± 0.17 |  |  | 36* |  |
|  |  | 10% bdECM + 1Si-CaP | 52* | 0.2* | 1.2* |  |  | 36* |  |
|  |  | 20%bdECM + 0.5 Si-CaP | 40* | 0.17* | 1* |  |  | 32* |  |
|  |  | 20%bdECM+ 1Si-CaP | 30* | 0.19* | 1.4* |  |  | 40* |  |
| [11] | Inverse orthogonal (round pores) | β-TCP + PEGDA | 40 | 0.35* | 27* |  |  |  |  |
| **Full-ceramic scaffolds** | | | | | | | | | |
| [12] | Trabecular | HA | 80 | 1.6 | 513 |  |  |  |  |
| [13] | Inverse orthogonal (squared pores) | βTCP | 40 | 9.89 |  |  |  |  |  |
|  | Inverse orthogonal (round pores) |  | 44 | 4.11 |  |  |  |  |  |
| [14] | Orthogonal | βTCP | 45 | 23.54 |  |  |  | 0.8 |  |
| [15] | Sheet-Gyroid | HA | 22.7 |  |  | 15 |  |  |  |
|  |  |  | 48.5 |  |  | 7.7 |  |  |  |
|  |  |  | 64.5 |  |  | 10.6 |  |  |  |
|  | Sheet-Diamond |  | 29.5 |  |  | 22.9 |  |  |  |
|  |  |  | 47.8 |  |  | 13.6 |  |  |  |
|  |  |  | 64.3 |  |  | 11.7 |  |  |  |
|  | Sheet-Schwarz Primitive |  | 30.4 |  |  | 15.3 |  |  |  |
|  |  |  | 45.8 |  |  | 6.5 |  |  |  |
|  |  |  | 66.6 |  |  | 10 |  |  |  |
| [16] | Solid-IWP | HA + ZrO2 | 54.6 | 52.25 | 4500 |  |  |  |  |
| [17] | Solid-IWP | HA | 49.8 | 15.25 | 970 | 41.3 |  |  |  |
| [18] | Gyroid | HA + AK | 50.5 | 2 | 300 |  |  | 2 |  |
|  | Gyroid Linear |  | 45 | 3.5 | 500 |  |  | 1.6 |  |
|  | Gyroid Quadratic |  | 45 | 3.7 | 550 |  |  | 1.6 |  |
|  | Gyroid Exponential |  | 42.8 | 4.3 | 600 |  |  | 1.6 |  |
| [19] | Solid-Gyroid | HA | 70* | 9 |  |  |  |  |  |
|  |  | HA_5AK | 77* | 2.2 |  |  |  |  |  |
|  |  | HA_10AK | 80* | 1.8 |  |  |  |  |  |
|  |  | HA_20AK | 81* | 1.6 |  |  |  |  |  |
| [20] | Sheet-Schwarz Primitive | HA-Zr | 31.67 | 14.21 | 1030 |  |  | 1.7 |  |
|  | framed hexahedral |  |  | 11.61 | 1260 |  |  | 1 |  |
|  | Hexahedral |  |  | 5.01 | 600 |  |  | 1.2 |  |
| [21] | Inverse orthogonal (squared pores) | HA | 54.52 | 1.45 |  |  |  | 0.8* |  |
|  |  | HA | 52.41 | 1.76 |  |  |  | 0.8* |  |
|  |  | HA | 49.32 | 1.92 |  |  |  | 0.8* |  |
| [22] | Orthogonal | βTCP | 50 | 2.26 | 110 |  |  |  |  |
|  |  | 90βTCP_10MgO |  | 0.74 | 70 |  |  |  |  |
|  |  | 80βTCP_20MgO |  | 1.4 | 90 |  |  |  |  |
|  |  | 70βTCP_30MgO |  | 2.99 | 130 |  |  |  |  |
|  |  | 60βTCP_40MgO |  | 4.48 | 190 |  |  |  |  |
| [23] | Solid-Gyroid | βTCP | 64.79 | 10.42 |  |  |  |  |  |
|  |  |  | 62.08 | 13.04 |  |  |  |  |  |
|  |  |  | 56.42 | 16.53 |  |  |  |  |  |
| [24] | Diamond | BCP + BG | 70 | 1.7 |  |  |  |  |  |
| [25] | Gyroid | βTCP | 66 | 8.61 |  |  |  | 1.3 |  |
| [26] | Voronoi | HA-Zr | 35.53 | 19.4 | 174.18 |  |  |  |  |
|  |  |  | 48.55 | 13.41 | 128.64 |  |  |  |  |
|  |  |  | 61.75 | 9.95 | 103.5 |  |  |  |  |
| [27] | Sheet-Gyroid | BCP | 76.4 | 3.2 ± 0.9 | 122 ± 21 |  |  | 4 |  |
|  |  |  | 74* | 7* | 165* |  |  | 4.2 |  |
|  |  |  | 66.7 | 11.5 ± 0.9 | 229 ± 12 |  |  | 5 |  |
| [28] | Sheet-Gyroid | BCP | 77.5 | 4.3 | 160 |  |  | 2.5* |  |
|  | 20-Elongated Gyroid |  | 76.1 | 6* | 180* |  |  | 3* |  |
|  | 40-Elongated Gyroid |  | 73.8 | 7* | 190* |  |  | 3.5* |  |
|  | 60-Elongated Gyroid |  | 70.1 | 11.5 | 240 |  |  | 5.5* |  |
| [29] | Sheet-Gyroid | BCP | 79.3 | 2.1 ± 0.2 | 72.5 |  |  | 2.7* |  |
|  |  |  | 79.2 | 1.7* | 63* |  |  | 2.4* |  |
|  |  |  | 79.8 | 1.5* | 58* |  |  | 2.4* |  |
|  |  |  | 80.4 | 0.8 ± 0.1 | 45.7 |  |  | 2* |  |
| [30] | Solid-Gyroid | βTCP-BG | 68.39 | 11.43 |  |  |  |  |  |
|  |  |  | 70.2* | 10* |  |  |  |  |  |
|  |  |  | 75.76* | 7.5* |  |  |  |  |  |
|  |  |  | 79.21 | 6.31 |  |  |  |  |  |
| [31] | BCC | HA | 70.43 | 3.4 | 150 |  |  |  |  |
|  | Sheet-Shwarz Primitive |  | 67.29 | 5.9 | 200 |  |  |  |  |
|  | Orthogonal |  | 68.39 | 22.5 | 400 |  |  |  |  |
| [32] | Orthogonal | BCP | 72.65 | 1.06 | 18.11 |  |  |  |  |
|  |  | BCP/GelMA |  | 1.39 | 26.28 |  |  |  |  |
|  |  | BCP/GelMA/PL |  | 1.45 | 27.28 |  |  |  |  |
| [33] | Solid-Gyroid | HA | 70 | 14.88 |  |  |  |  |  |
| [34] | BCC | HA | 60 | 0.42 |  |  |  | 3* |  |
|  | FCC |  |  | 1.46 |  |  |  | 3* |  |
|  | Solid-Gyroid |  |  | 3.32 |  |  |  | 4* |  |
| [35] | Voronoi | βTCP | 43.4 | 4.1 |  |  |  |  |  |
|  |  |  | 53.1 | 2.5 |  |  |  |  |  |
|  |  |  | 62.4 | 1.4 |  |  |  |  |  |
|  |  |  | 72.9 | 0.8 |  |  |  |  |  |
| [36] | Diamond | HA | 68.25 | 3.5 | 50 |  |  |  |  |
|  | Diamond + straight central flow |  | 70.2* | 2.1* | 25* |  |  |  |  |
|  | Diamond + curved central flow |  | 70.3* | 2.1* | 25* |  |  |  |  |
| [37] | Inverse orthogonal (round pores) | HA | 31.4 | 4.2 (\|\|) |  |  |  |  |  |
|  |  |  | 30.6 | 4.9 (45°) |  |  |  |  |  |
|  |  |  | 32.7 | 4.8 (⊥) |  |  |  |  |  |
| [38] | Orthogonal | βTCP | 44 | 13 |  |  |  | 2* |  |
|  |  |  |  | 23 |  |  |  | 3* |  |
| [39] | Orthogonal | βTCP | 56 | 11 |  |  |  | 3* |  |
|  | Orthogonal hollow |  | 63 | 4 |  |  |  |  |  |
|  | Orthogonal hollow |  | 69 | 3 |  |  |  |  |  |
|  | Orthogonal infiltrated |  | 56 | 9 |  |  |  |  |  |
|  | Orthogonal infiltrated |  | 56 | 9 |  |  |  |  |  |
| [40] | Orthogonal | βTCP | 47 | 19 |  | 9 |  | 4* |  |
|  | Orthogonal hollow |  | 59 | 5 |  | 7 |  | 2* |  |
|  | Orthogonal infiltrated |  | 47 | 6 |  | 8.5 |  |  |  |
| [41] | Orthogonal | βTCP | 52 | 31 ⊥ |  |  |  | 2.5* |  |
|  | Orthogonal |  |  | 36 \|\| |  |  |  | 3.5* |  |
|  | Solid-Schwarz Primitive |  |  | 33 ⊥ |  |  |  | 2* |  |
|  | Solid-Schwarz Primitive |  |  | 45 \|\| |  |  |  | 3.5* |  |
|  | Orthogonal |  |  | 49 ⊥ |  |  |  | 3.5* |  |
|  | Orthogonal |  |  | 51 \|\| |  |  |  | 4* |  |
| [42] | Orthogonal | βTCP | 74 | 5 |  |  |  | 2* |  |
|  |  |  | 69 | 13 |  |  |  | 3.1* |  |
|  |  |  | 78 | 2.9 |  |  |  | 2.9* |  |
|  |  |  | 75 | 5 |  |  |  | 3* |  |
| [43] | Orthogonal | βTCP | 64.8 (designed) | 9 |  |  |  | 4* |  |
|  |  |  |  | 11 |  |  |  | 4.1* |  |
|  |  |  |  | 11.5 |  |  |  | 4* |  |
|  |  |  |  | 7 |  |  |  | 3* |  |
|  |  |  |  | 7.5 |  |  |  | 2* |  |
|  |  |  |  | 22 |  |  |  | 4.2* |  |
|  |  |  |  | 7 |  |  |  | 2.2* |  |
|  |  |  |  | 4 |  |  |  | 2* |  |
|  |  |  |  | 5 |  |  |  | 2* |  |
|  |  |  |  | 9 |  |  |  | 1.7* |  |
|  |  |  |  | 12.5 |  |  |  | 2* |  |
|  |  |  |  | 12.6 |  |  |  | 4* |  |
| [44] | Sheet-Gyroid | βTCP | 67 | 2.84 ± 0.42 |  |  |  |  |  |
|  |  | 1Mg-βTCP | 67 | 5.87 ± 0.51 |  |  |  |  |  |
|  |  | 3Mg-βTCP | 67 | 8.91 ± 0.55 |  |  |  |  |  |
|  |  | 5Mg-βTCP | 67 | 6.9 ± 0.65 |  |  |  |  |  |
| [45] | Trabecular | HA | 75.55 | 0.36 | 20.52 |  |  |  |  |
|  |  | HA | 74.83 | 0.41 | 28.83 |  |  |  |  |
|  |  | HA | 75.54 | 1.1 | 62.23 |  |  |  |  |
|  |  | HA | 77.43 | 2.3 | 105.26 |  |  |  |  |
|  |  | HA | 77.84 | 1.32 | 72.61 |  |  |  |  |
|  |  | HA-Ca-Sr-Zn-Mg | 75.55 | 0.5 | 31.39 |  |  | 0.03* |  |
|  |  | HA-Ca-Sr-Zn-Mg | 74.83 | 0.82 | 58.48 |  |  | 0.03* |  |
|  |  | HA-Ca-Sr-Zn-Mg | 75.54 | 1.9 | 98.56 |  |  | 0.03* |  |
|  |  | HA-Ca-Sr-Zn-Mg | 77.43 | 2.76 | 129.51 |  |  | 0.03* |  |
|  |  | HA-Ca-Sr-Zn-Mg | 77.84 | 3.37 | 165.95 |  |  | 0.03* |  |
| [46] | Cubic | βTCP | 17.89 ± 2.37 | 34.1 ± 11.6 |  |  |  |  |  |
|  | Cubic |  | 40.07 ± 0.16 | 7.9 ± 1.5 |  |  |  |  |  |
|  | Cubic |  | 47.93 ± 1.41 | 7.2 ± 1.5 |  |  |  |  |  |
|  | Octahedral |  | 9.16 ± 0.72 | 25.4 ± 9.7 |  |  |  |  |  |
|  | Octahedral |  | 34.46 ± 0.62 | 8.1 ± 1.2 |  |  |  |  |  |
|  | Octahedral |  | 59.36 ± 0.18 | 1.4 ± 0.5 |  |  |  |  |  |
|  | Hexagonal |  | 5.58 ± 1.03 | 67.6 ± 13.3 |  |  |  |  |  |
|  | Hexagonal |  | 19.87 ± 1.03 | 36.6 ± 13.9 |  |  |  |  |  |
|  | Hexagonal |  | 25.14 ± 1.89 | 26.5 ± 6.7 |  |  |  |  |  |
| [47] | orthogonal (grid) | β-TCP | 49 | 44.7 |  |  |  |  |  |
|  | orthogonal (grid) |  | 74 | 14.2 |  |  |  |  |  |
|  | Kagome |  | 40 | 19.5 |  |  |  |  |  |
|  | Kagome |  | 65 | 6.75 |  |  |  |  |  |
| [48] | Solid-Gyroid | BCP | 30.55 | 1.66 |  |  |  |  |  |
|  |  | BCP-20BG | 40.8 | 0.57 |  |  |  |  |  |
|  |  | BCP-30BG | 45.65 | 1.12 |  |  |  |  |  |
|  |  | BCP-40BG | 63.2 | 1.29 |  |  |  |  |  |
| [49] | Cellular (gyroid-like) | HA | 77 | 0.36 |  |  |  |  |  |
| [50] | Hollow tube structure (similar to coaxial robocasting) | βTCP | 33.12 | 2.07 |  |  |  |  |  |
|  |  |  | 40* | 1.9* |  |  |  |  |  |
|  |  |  | 45* | 1.4* |  |  |  |  |  |
|  |  |  | 52.91 | 0.74 |  |  |  |  |  |
| [51] | Orthogonal | βTCP | 68.2 | 1.35 |  |  |  |  |  |
|  |  | βTCP-Pyritium | 66.11 | 5.5 |  |  |  |  |  |
| [52] | Orthogonal cubic | BCP | 68.36 | 3.95 |  |  |  |  |  |
|  | Octet-truss |  | 70.27 | 4.09 |  |  |  |  |  |
|  | Inverse FCC |  | 73.45 | 4.96 |  |  |  |  |  |
| [53] | Orthogonal cubic | BCP | 68.4 | 4* |  |  |  |  |  |
|  |  |  | 60.9 | 7* |  |  |  |  |  |
|  |  |  | 56.3 | 20.07 |  |  |  |  |  |
| [54] | Solid-Shwarz Primitive | BCP 50 wt.% | 72.61 | 3.6 |  |  |  |  |  |
|  |  | BCP 55 wt.% | 67.3 | 6.87 |  |  |  |  |  |
|  |  | BCP 60 wt.% | 64.5 | 9.03 |  |  |  |  |  |
| [55] | Orthogonal dense strut | βTCP | 62.8 | 14.2 |  | 10.4 | 5000 | 2 |  |
|  | Orthogonal hollow strut |  |  | 4.6 |  | 4.6 | 3000 | 2.5 |  |
|  | Orthogonal infiltrataed struts |  |  | 13.8 |  | 9.2 | 4800 | 3 |  |
| [56] | orthogonal (cylindrical structures as pores) | βTCP | 41.70% | 9* |  |  |  |  |  |
|  |  |  |  | 34* |  |  |  |  |  |
|  |  |  |  | 36* |  |  |  |  |  |
|  |  |  |  | 39* |  |  |  |  |  |
| [57] | BCC | BCP | 71.6 | 306* | 670* |  |  |  |  |
| [58] | orthogonal | βTCP | 60* | 37.67 |  |  |  |  |  |
|  | Sheet-Gyroid |  | 60* | 61.93 |  |  |  |  |  |
|  | Sheet-Gyroid |  | 60* | 58.27 |  |  |  |  |  |
|  | Sheet-Gyroid |  | 60* | 61.13 |  |  |  |  |  |
| [59] | Woodpile | BCP | 67.3 | 3.5* |  |  |  |  |  |
| [60] | Solid-Gyroid | αTCP | 63.99 | 15* | 700* |  |  |  |  |
|  |  | βTCP | 56.06 | 15* | 700* |  |  |  |  |
| [61] | FCC | βTCP | 83 | 0.8* |  |  |  | 0.75* |  |
|  | FCC |  | 83 | 0.55* |  |  |  | 0.75* |  |
|  | Sheet-gyroid |  | 83 | 1.5* |  |  |  | 0.75* |  |
|  | Solid-gyroid |  | 89 | 0.5* |  |  |  | 0.75* |  |
|  | Sheet-Gyroid |  | 80 | 1.91 | 690 |  |  |  |  |
|  |  |  | 58 | 20.14 | 2660 |  |  |  |  |
|  |  |  | 49 | 29.17 | 3620 |  |  |  |  |
| [62] | Solid-gyroid | βTCP | 68* | 5* |  |  |  |  |  |
|  |  | βTCP-2Laponite | 66* | 5* |  |  |  |  |  |
|  |  | βTCP-5Laponite | 62.5* | 5* |  |  |  |  |  |
|  |  | βTCP-10Laponite | 61.5* | 5.5* |  |  |  |  |  |
| [63] | Solid-gyroid | βTCP | 40 | 43.6 |  |  |  |  |  |
|  |  |  | 45 | 22* |  |  |  |  |  |
|  |  |  | 60 | 10* |  |  |  |  |  |
|  |  |  | 75 | 4* |  |  |  |  |  |
|  |  |  | 87 | 0.6 |  |  |  |  |  |
| [64] | Trabecular | HA + Sr^2+^, Mg^2+^, Zn^2+^ | 77.43 | 2.76 | 130 |  |  | 3* |  |
| [65] | Solid-Swarz-Primitive | HA | 35* | 32.5 | 850* |  |  | 5* |  |
|  |  | HA+20BR | 27.5* | 15 | 600* |  |  | 3.5* |  |
| [66] | FCC/Octet | HA | 76.59 | 8.03 |  |  |  |  |  |
|  |  |  | 56.27 |  |  | 3.20 ± 0.27 |  |  |  |
| [67] | Diamond | HA-10ZnO | 51.6 | 13.7 |  |  |  | 5 |  |
| [68] | BCC | HA | 30* | 2.3* |  |  |  |  |  |
| [69] | Solid-gyroid | HA | 70 (designed) | 2.135 ± 0.51 |  |  |  |  |  |
| [70] | Solid-gyroid | βTCP-15BG | 48.98 | 4.5 | 500 |  |  |  |  |
|  |  |  | 48.98 | 6.5 | 700 |  |  |  |  |
|  |  |  | 48.98 | 8.5 | 800 |  |  |  |  |

# References

[1] H. Akbari-Aghdam, A. Bagherifard, M. Motififard, J. Parvizi, E. Sheikhbahaei, S. Esmaeili, S. Saber-Samandari, A. Khandan, Development of Porous Photopolymer Resin-SWCNT Produced by Digital Light Processing Technology Using for Bone Femur Application., Arch. Bone Jt. Surg. 9 (2021) 445–452. https://doi.org/10.22038/abjs.2020.43409.2189.

[2] A. Bagheri Saed, A.H. Behravesh, S. Hasannia, B. Akhoundi, S.K. Hedayati, F. Gashtasbi, An in vitro study on the key features of Poly L-lactic acid/biphasic calcium phosphate scaffolds fabricated via DLP 3D printing for bone grafting, Eur. Polym. J. 141 (2020) 110057. https://doi.org/10.1016/j.eurpolymj.2020.110057.

[3] S. Channasanon, P. Kaewkong, S. Chantaweroad, P. Tesavibul, Y. Pratumwal, S. Otarawanna, S. Kirihara, S. Tanodekaew, Scaffold geometry and computational fluid dynamics simulation supporting osteogenic differentiation in dynamic culture, Comput. Methods Biomech. Biomed. Engin. 27 (2024) 587–598. https://doi.org/10.1080/10255842.2023.2195961.

[4] D. Chen, G. Chen, X. Zhang, J. Chen, J. Li, K. Kang, W. He, Y. Kong, L. Wu, B. Su, K. Zhao, D. Si, X. Wang, Fabrication and in vitro evaluation of 3D printed porous silicate substituted calcium phosphate scaffolds for bone tissue engineering, Biotechnol. Bioeng. 119 (2022) 3297–3310. https://doi.org/10.1002/bit.28202.

[5] C. Duque, C.A. Gómez-Tirado, S. Ocampo, L.M. Arroyave-Muñoz, L.M. Restrepo-Munera, A.F. Vásquez, A. Ossa, C. García, Obtaining biocompatible polymeric scaffolds loaded with calcium phosphates through the digital light processing technique, J. Mater. Res. 39 (2024) 1886–1900. https://doi.org/10.1557/s43578-023-01144-0.

[6] C. Oliver-Urrutia, L. Drotárová, S. Gascón-Pérez, K. Slámečka, S. Ravaszová, L. Čelko, E.B. Montufar, Hydroxyapatite-Resin Composites Produced by Vat Photopolymerization and Post-Processing via In Situ Hydrolysis of Alpha Tricalcium Phosphate, Ceramics 6 (2023) 2282–2294. https://doi.org/10.3390/ceramics6040139.

[7] A. Ronca, L. Ambrosio, D.W. Grijpma, Design of Porous Three-Dimensional PDLLA/nano-hap Composite Scaffolds Using Stereolithography, J. Appl. Biomater. Funct. Mater. 10 (2012) 249–258. https://doi.org/10.5301/JABFM.2012.10211.

[8] A. Tikhonov, P. Evdokimov, E. Klimashina, S. Tikhonova, E. Karpushkin, I. Scherbackov, V. Dubrov, V. Putlayev, Stereolithographic fabrication of three-dimensional permeable scaffolds from CaP/PEGDA hydrogel biocomposites for use as bone grafts, J. Mech. Behav. Biomed. Mater. 110 (2020) 103922. https://doi.org/10.1016/j.jmbbm.2020.103922.

[9] B. Wang, X. Ye, G. Chen, Y. Zhang, Z. Zeng, C. Liu, Z. Tan, X. Jie, Fabrication and properties of PLA/β-TCP scaffolds using liquid crystal display (LCD) photocuring 3D printing for bone tissue engineering, Front. Bioeng. Biotechnol. 12 (2024). https://doi.org/10.3389/fbioe.2024.1273541.

[10] D. Liu, J. Liu, P. Zhao, Z. Peng, Z. Geng, J. Zhang, Z. Zhang, R. Shen, X. Li, X. Wang, S. Li, J. Wang, X. Wang, 3D Bioprinted Tissue‐Engineered Bone with Enhanced Mechanical Strength and Bioactivities: Accelerating Bone Defect Repair through Sequential Immunomodulatory Properties, Adv. Healthc. Mater. 13 (2024). https://doi.org/10.1002/adhm.202401919.

[11] W. Zhang, Q. Lian, D. Li, K. Wang, D. Hao, W. Bian, Z. Jin, The effect of interface microstructure on interfacial shear strength for osteochondral scaffolds based on biomimetic design and 3D printing, Mater. Sci. Eng. C 46 (2015) 10–15. https://doi.org/10.1016/j.msec.2014.09.042.

[12] F. Baino, G. Magnaterra, E. Fiume, A. Schiavi, L. Tofan, M. Schwentenwein, E. Verné, Digital light processing stereolithography of hydroxyapatite scaffolds with bone‐like architecture, permeability, and mechanical properties, J. Am. Ceram. Soc. 105 (2022) 1648–1657. https://doi.org/10.1111/jace.17843.

[13] S. Liu, L. Mo, G. Bi, S. Chen, D. Yan, J. Yang, Y.-G. Jia, L. Ren, DLP 3D printing porous β-tricalcium phosphate scaffold by the use of acrylate/ceramic composite slurry, Ceram. Int. 47 (2021) 21108–21116. https://doi.org/10.1016/j.ceramint.2021.04.114.

[14] W. Bian, D. Li, Q. Lian, W. Zhang, L. Zhu, X. Li, Z. Jin, Design and fabrication of a novel porous implant with pre-set channels based on ceramic stereolithography for vascular implantation, Biofabrication 3 (2011) 034103. https://doi.org/10.1088/1758-5082/3/3/034103.

[15] I. Bouakaz, E. Sadeghian Dehkord, S. Meille, A. Schrijnemakers, F. Boschini, N. Preux, S. Hocquet, L. Geris, G. Nolens, D. Grossin, A. Dupret-Bories, 3D printed triply periodic minimal surfaces calcium phosphate bone substitute: The effect of porosity design on mechanical properties, Ceram. Int. 50 (2024) 2623–2636. https://doi.org/10.1016/j.ceramint.2023.10.238.

[16] Y. Cao, T. Shi, C. Jiao, H. Liang, R. Chen, Z. Tian, A. Zou, Y. Yang, Z. Wei, C. Wang, L. Shen, Fabrication and properties of zirconia/hydroxyapatite composite scaffold based on digital light processing, Ceram. Int. 46 (2020) 2300–2308. https://doi.org/10.1016/j.ceramint.2019.09.219.

[17] Z. Liu, H. Liang, T. Shi, D. Xie, R. Chen, X. Han, L. Shen, C. Wang, Z. Tian, Additive manufacturing of hydroxyapatite bone scaffolds via digital light processing and in vitro compatibility, Ceram. Int. 45 (2019) 11079–11086. https://doi.org/10.1016/j.ceramint.2019.02.195.

[18] Z.-L. Deng, M.-Z. Pan, S.-B. Hua, J.-M. Wu, X.-Y. Zhang, Y.-S. Shi, Mechanical and degradation properties of triply periodic minimal surface (TPMS) hydroxyapatite &amp; akermanite scaffolds with functional gradient structure, Ceram. Int. 49 (2023) 20808–20816. https://doi.org/10.1016/j.ceramint.2023.03.213.

[19] S.-B. Hua, X. Yuan, J.-M. Wu, J. Su, L.-J. Cheng, W. Zheng, M.-Z. Pan, J. Xiao, Y.-S. Shi, Digital light processing porous TPMS structural HA &amp; akermanite bioceramics with optimized performance for cancellous bone repair, Ceram. Int. 48 (2022) 3020–3029. https://doi.org/10.1016/j.ceramint.2021.10.003.

[20] Y. Du, T. Hu, J. You, Y. Ye, B. Zhang, B. Bao, M. Li, Y. Liu, Y. Wang, T. Wang, Study of falling‐down‐type DLP 3D printing technology for high‐resolution hydroxyapatite scaffolds, Int. J. Appl. Ceram. Technol. 19 (2022) 268–280. https://doi.org/10.1111/ijac.13915.

[21] C. Feng, K. Zhang, R. He, G. Ding, M. Xia, X. Jin, C. Xie, Additive manufacturing of hydroxyapatite bioceramic scaffolds: Dispersion, digital light processing, sintering, mechanical properties, and biocompatibility, J. Adv. Ceram. 9 (2020) 360–373. https://doi.org/10.1007/s40145-020-0375-8.

[22] M. Ge, D. Xie, C. Jiao, Y. Yang, L. Shen, M. Qiu, H. Zhang, Z. He, H. Liang, Z. Tian, Mechanical properties and biocompatibility of MgO / Ca3(PO4)2 composite ceramic scaffold with high MgO content based on digital light processing, Ceram. Int. 48 (2022) 21175–21186. https://doi.org/10.1016/j.ceramint.2022.04.010.

[23] Z. Hang, X. Songfeng, X. Yinze, G. Ruining, L. Xiang, Fabrication of Porous β β -TCP Bioceramics Using Digital Light Processing, J. Mech. Eng. 55 (2019) 81. https://doi.org/10.3901/JME.2019.15.081.

[24] S.-B. Hua, J. Su, Z.-L. Deng, J.-M. Wu, L.-J. Cheng, X. Yuan, F. Chen, H. Zhu, D.-H. Qi, J. Xiao, Y.-S. Shi, Microstructures and properties of 45S5 bioglass® &amp; BCP bioceramic scaffolds fabricated by digital light processing, Addit. Manuf. 45 (2021) 102074. https://doi.org/10.1016/j.addma.2021.102074.

[25] X. Huang, H. Dai, Y. Hu, P. Zhuang, Z. Shi, Y. Ma, Development of a high solid loading β-TCP suspension with a low refractive index contrast for DLP -based ceramic stereolithography, J. Eur. Ceram. Soc. 41 (2021) 3743–3754. https://doi.org/10.1016/j.jeurceramsoc.2020.12.047.

[26] C. Jiao, D. Xie, Z. He, H. Liang, L. Shen, Y. Yang, Z. Tian, G. Wu, C. Wang, Additive manufacturing of Bio-inspired ceramic bone Scaffolds: Structural Design, mechanical properties and biocompatibility, Mater. Des. 217 (2022) 110610. https://doi.org/10.1016/j.matdes.2022.110610.

[27] J.-W. Kim, J.-B. Lee, Y.-H. Koh, H.-E. Kim, Digital Light Processing of Freeze-cast Ceramic Layers for Macroporous Calcium Phosphate Scaffolds with Tailored Microporous Frameworks, Materials (Basel). 12 (2019) 2893. https://doi.org/10.3390/ma12182893.

[28] J.-W. Lee, Y.-H. Lee, H. Lee, Y.-H. Koh, H.-E. Kim, Improving mechanical properties of porous calcium phosphate scaffolds by constructing elongated gyroid structures using digital light processing, Ceram. Int. 47 (2021) 3252–3258. https://doi.org/10.1016/j.ceramint.2020.09.164.

[29] Y.-H. Lee, J.-W. Lee, S.-Y. Yang, H. Lee, Y.-H. Koh, H.-E. Kim, Dual-scale porous biphasic calcium phosphate gyroid scaffolds using ceramic suspensions containing polymer microsphere porogen for digital light processing, Ceram. Int. 47 (2021) 11285–11293. https://doi.org/10.1016/j.ceramint.2020.12.254.

[30] X. Li, H. Zhang, Y. Shen, Y. Xiong, L. Dong, J. Zheng, S. Zhao, Fabrication of porous β-TCP/58S bioglass scaffolds via top-down DLP printing with high solid loading ceramic-resin slurry, Mater. Chem. Phys. 267 (2021) 124587. https://doi.org/10.1016/j.matchemphys.2021.124587.

[31] H. Liang, Y. Wang, S. Chen, Y. Liu, Z. Liu, J. Bai, Nano-Hydroxyapatite Bone Scaffolds with Different Porous Structures Processed by Digital Light Processing 3D Printing, Int. J. Bioprinting 8 (2022) 502. https://doi.org/10.18063/ijb.v8i1.502.

[32] G. Liu, B. Zhang, T. Wan, C. Zhou, Y. Fan, W. Tian, W. Jing, A 3D-printed biphasic calcium phosphate scaffold loaded with platelet lysate/gelatin methacrylate to promote vascularization, J. Mater. Chem. B 10 (2022) 3138–3151. https://doi.org/10.1039/D2TB00006G.

[33] K. Liu, X. Wu, J. Liu, H. Yang, M. Li, T. Qiu, H. Dai, Design and manufacture of a customized, large-size and high-strength bioactive HA osteoid composite ceramic by stereolithography, Ceram. Int. 49 (2023) 11630–11640. https://doi.org/10.1016/j.ceramint.2022.12.010.

[34] K. Liu, Q. Zhou, X. Zhang, L. Ma, B. Xu, R. He, Morphologies, mechanical and in vitro behaviors of DLP-based 3D printed HA scaffolds with different structural configurations, RSC Adv. 13 (2023) 20830–20838. https://doi.org/10.1039/D3RA03080F.

[35] S. Liu, J. Chen, T. Chen, Y. Zeng, Fabrication of trabecular-like beta-tricalcium phosphate biomimetic scaffolds for bone tissue engineering, Ceram. Int. 47 (2021) 13187–13198. https://doi.org/10.1016/j.ceramint.2021.01.184.

[36] R. Mao, Y. Lai, D. Li, Y. Huang, L. Wang, F. Luo, Y. Chen, J. Lu, X. Ge, Y. Liu, Y. Fan, X. Zhang, Q. Jiang, K. Wang, Flow channel performance in 3D printed hydroxyapatite scaffolds to improve metabolism and tissue ingrowth in flat bone repair, Compos. Part B Eng. 259 (2023) 110727. https://doi.org/10.1016/j.compositesb.2023.110727.

[37] P. Navarrete-Segado, M. Tourbin, C. Frances, D. Grossin, Masked stereolithography of hydroxyapatite bioceramic scaffolds: From powder tailoring to evaluation of 3D printed parts properties, Open Ceram. 9 (2022) 100235. https://doi.org/10.1016/j.oceram.2022.100235.

[38] C. Paredes, F.J. Martínez-Vázquez, H. Elsayed, P. Colombo, A. Pajares, P. Miranda, Evaluation of direct light processing for the fabrication of bioactive ceramic scaffolds: Effect of pore/strut size on manufacturability and mechanical performance, J. Eur. Ceram. Soc. 41 (2021) 892–900. https://doi.org/10.1016/j.jeurceramsoc.2020.09.002.

[39] C. Paredes, F.J. Martínez-Vázquez, H. Elsayed, P. Colombo, A. Pajares, P. Miranda, Using ductile cores for enhancing the mechanical performance of hollow strut β-TCP scaffolds fabricated by digital light processing, Ceram. Int. 47 (2021) 10163–10173. https://doi.org/10.1016/j.ceramint.2020.12.165.

[40] C. Paredes, F.J. Martínez-Vázquez, A. Pajares, P. Miranda, Co-continuous calcium phosphate/polycaprolactone composite bone scaffolds fabricated by digital light processing and polymer melt suction, Ceram. Int. 47 (2021) 17726–17735. https://doi.org/10.1016/j.ceramint.2021.03.093.

[41] C. Paredes, J. Roleček, P. Miranda, Improving the strength of β-TCP scaffolds produced by Digital Light Processing using two-step sintering, J. Eur. Ceram. Soc. 44 (2024) 2571–2580. https://doi.org/10.1016/j.jeurceramsoc.2023.11.028.

[42] C. Paredes, J. Roleček, L. Pejchalová, P. Miranda, D. Salamon, Impact of residual carbon after DLP and SPS-Sintering on compressive strength and in-VITRO bioactivity of calcium phosphate scaffolds, Open Ceram. 11 (2022) 100281. https://doi.org/10.1016/j.oceram.2022.100281.

[43] C. Paredes, J. Roleček, L. Pejchalová, T. Spusta, D. Salamon, P. Miranda, Evaluating the suitability of fast sintering techniques for the consolidation of calcium phosphate scaffolds produced by DLP, J. Eur. Ceram. Soc. 43 (2023) 6493–6503. https://doi.org/10.1016/j.jeurceramsoc.2023.05.052.

[44] D. Qi, J. Su, S. Li, H. Zhu, L. Cheng, S. Hua, X. Yuan, J. Jiang, Z. Shu, Y. Shi, J. Xiao, 3D printed magnesium-doped β-TCP gyroid scaffold with osteogenesis, angiogenesis, immunomodulation properties and bone regeneration capability in vivo, Biomater. Adv. 136 (2022) 212759. https://doi.org/10.1016/j.bioadv.2022.212759.

[45] A. Ressler, S. Zakeri, J. Dias, M. Hannula, J. Hyttinen, H. Ivanković, M. Ivanković, S. Miettinen, M. Schwentenwein, E. Levänen, E.J. Frankberg, Vat photopolymerization of biomimetic bone scaffolds based on Mg, Sr, Zn-substituted hydroxyapatite: Effect of sintering temperature, Ceram. Int. 50 (2024) 27403–27415. https://doi.org/10.1016/j.ceramint.2024.05.038.

[46] E. Ryan, S. Yin, Compressive strength of β-TCP scaffolds fabricated via lithography-based manufacturing for bone tissue engineering, Ceram. Int. 48 (2022) 15516–15524. https://doi.org/10.1016/j.ceramint.2022.02.085.

[47] C. Schmidleithner, S. Malferrari, R. Palgrave, D. Bomze, M. Schwentenwein, D.M. Kalaskar, Application of high resolution DLP stereolithography for fabrication of tricalcium phosphate scaffolds for bone regeneration, Biomed. Mater. 14 (2019) 045018. https://doi.org/10.1088/1748-605X/ab279d.

[48] J. Su, S. Hua, A. Chen, P. Chen, L. Yang, X. Yuan, D. Qi, H. Zhu, C. Yan, J. Xiao, Y. Shi, Three-dimensional printing of gyroid-structured composite bioceramic scaffolds with tuneable degradability, Biomater. Adv. 133 (2022) 112595. https://doi.org/10.1016/j.msec.2021.112595.

[49] P. Tesavibul, S. Chantaweroad, A. Laohaprapanon, S. Channasanon, P. Uppanan, S. Tanodekaew, P. Chalermkarnnon, K. Sitthiseripratip, Biocompatibility of hydroxyapatite scaffolds processed by lithography-based additive manufacturing, Biomed. Mater. Eng. 26 (2015) 31–38. https://doi.org/10.3233/BME-151549.

[50] Y. Tian, H. Ma, X. Yu, B. Feng, Z. Yang, W. Zhang, C. Wu, Biological response of 3D-printed β-tricalcium phosphate bioceramic scaffolds with the hollow tube structure, Biomed. Mater. 18 (2023) 034102. https://doi.org/10.1088/1748-605X/acc374.

[51] D. Wang, J. Hou, C. Xia, C. Wei, Y. Zhu, W. Qian, S. Qi, Y. Wu, Y. Shi, K. Qin, L. Wu, F. Yin, Z. Chen, W. Li, Multi-element processed pyritum mixed to β-tricalcium phosphate to obtain a 3D-printed porous scaffold: An option for treatment of bone defects, Mater. Sci. Eng. C 128 (2021) 112326. https://doi.org/10.1016/j.msec.2021.112326.

[52] J. Wang, Y. Tang, Q. Cao, Y. Wu, Y. Wang, B. Yuan, X. Li, Y. Zhou, X. Chen, X. Zhu, C. Tu, X. Zhang, Fabrication and biological evaluation of 3D-printed calcium phosphate ceramic scaffolds with distinct macroporous geometries through digital light processing technology, Regen. Biomater. 9 (2022) rbac005. https://doi.org/10.1093/rb/rbac005.

[53] Y. Wang, S. Chen, H. Liang, Y. Liu, J. Bai, M. Wang, Digital light processing (DLP) of nano biphasic calcium phosphate bioceramic for making bone tissue engineering scaffolds, Ceram. Int. 48 (2022) 27681–27692. https://doi.org/10.1016/j.ceramint.2022.06.067.

[54] Y. Wei, D. Zhao, Q. Cao, J. Wang, Y. Wu, B. Yuan, X. Li, X. Chen, Y. Zhou, X. Yang, X. Zhu, C. Tu, X. Zhang, Stereolithography-Based Additive Manufacturing of High-Performance Osteoinductive Calcium Phosphate Ceramics by a Digital Light-Processing System, ACS Biomater. Sci. Eng. 6 (2020) 1787–1797. https://doi.org/10.1021/acsbiomaterials.9b01663.

[55] Y. Wu, R. Chen, X. Chen, Y. Yang, J. Qiao, Y. Liu, Development of Strong and Tough β-TCP/PCL Composite Scaffolds with Interconnected Porosity by Digital Light Processing and Partial Infiltration, Materials (Basel). 16 (2023) 947. https://doi.org/10.3390/ma16030947.

[56] Y. Wu, X. Chen, G. Zhao, R. Chen, Y. Liu, H. Ren, X. Qu, Y. Liu, β-Tricalcium phosphate/ԑ-polycaprolactone composite scaffolds with a controllable gradient: Fabrication and characterization, Ceram. Int. 45 (2019) 16188–16194. https://doi.org/10.1016/j.ceramint.2019.05.140.

[57] Y. Wu, Q. Cao, Y. Wang, Y. Liu, X. Xu, P. Liu, X. Li, X. Zhu, X. Zhang, Optimized fabrication of DLP-based 3D printing calcium phosphate ceramics with high-precision and low-defect to induce calvarial defect regeneration, Mater. Des. 233 (2023) 112230. https://doi.org/10.1016/j.matdes.2023.112230.

[58] Y. Yang, T. Xu, H.-P. Bei, L. Zhang, C.-Y. Tang, M. Zhang, C. Xu, L. Bian, K.W.-K. Yeung, J.Y.H. Fuh, X. Zhao, Gaussian curvature–driven direction of cell fate toward osteogenesis with triply periodic minimal surface scaffolds, Proc. Natl. Acad. Sci. 119 (2022). https://doi.org/10.1073/pnas.2206684119.

[59] Z. Yang, L. Xie, B. Zhang, G. Zhang, F. Huo, C. Zhou, X. Liang, Y. Fan, W. Tian, Y. Tan, Preparation of BMP-2/PDA-BCP Bioceramic Scaffold by DLP 3D Printing and its Ability for Inducing Continuous Bone Formation, Front. Bioeng. Biotechnol. 10 (2022). https://doi.org/10.3389/fbioe.2022.854693.

[60] B. Zhang, X. Yin, F. Zhang, Y. Hong, Y. Qiu, X. Yang, Y. Li, C. Zhong, H. Yang, Z. Gou, Customized bioceramic scaffolds and metal meshes for challenging large-size mandibular bone defect regeneration and repair, Regen. Biomater. 10 (2023). https://doi.org/10.1093/rb/rbad057.

[61] F. Zhang, J. Yang, Y. Zuo, K. Li, Z. Mao, X. Jin, S. Zhang, H. Gao, Y. Cui, Digital light processing of β-tricalcium phosphate bioceramic scaffolds with controllable porous structures for patient specific craniomaxillofacial bone reconstruction, Mater. Des. 216 (2022) 110558. https://doi.org/10.1016/j.matdes.2022.110558.

[62] H. Zhang, K. Han, L. Dong, X. Li, Preparation and Characterization of β-tricalcium Phosphate/Nano Clay Composite Scaffolds via Digital Light Processing Printing, J. Inorg. Mater. 37 (2022) 1116. https://doi.org/10.15541/jim20210745.

[63] Y. Zhang, Q. Zhang, F. He, F. Zuo, X. Shi, Fabrication of cancellous-bone-mimicking β-tricalcium phosphate bioceramic scaffolds with tunable architecture and mechanical strength by stereolithography 3D printing, J. Eur. Ceram. Soc. 42 (2022) 6713–6720. https://doi.org/10.1016/j.jeurceramsoc.2022.07.033.

[64] A. Ressler, S. Zakeri, J. Dias, M. Hannula, J. Hyttinen, H. Ivanković, M. Ivanković, S. Miettinen, M. Schwentenwein, E. Levänen, E.J. Frankberg, Vat photopolymerization of biomimetic bone scaffolds based on Mg, Sr, Zn-substituted hydroxyapatite: Effect of sintering temperature, Ceram. Int. 50 (2024) 27403–27415. https://doi.org/10.1016/j.ceramint.2024.05.038.

[65] W. Guo, P. Li, Y. Wei, L. Zhao, Y. Pang, Y. Huang, X. Ye, S. Wang, B. Liu, H. You, Y. Long, Ionic substitution through bredigite doping for microstructure and performance adjustment in DLP 3D-printed TPMS porous HA bone scaffolds, Virtual Phys. Prototyp. 19 (2024). https://doi.org/10.1080/17452759.2024.2423840.

[66] J. Guo, X. Zhang, J. Yan, J. Wu, Y. Shi, S. Zhang, Digital light processing bio-scaffolds of hydroxyapatite ceramic foams with multi-level pores using Pickering emulsions as the feedstock, J. Eur. Ceram. Soc. 44 (2024) 4272–4284. https://doi.org/10.1016/j.jeurceramsoc.2024.01.021.

[67] X. Gui, B. Zhang, Y. Qin, H. Lei, X. Xia, Y. Li, H. Lei, X. Zhou, Y. Tan, Z. Dong, Q. You, C. Zhou, Y. Fan, Structural and material double mechanical enhancement of HAp scaffolds promote bone defect regeneration, Compos. Part A Appl. Sci. Manuf. 189 (2025) 108600. https://doi.org/10.1016/j.compositesa.2024.108600.

[68] X. Gao, J. Yang, X. Gan, Y. Lin, J. Xu, Z. Shan, Z. Han, S. Chen, B. Huang, B. Fan, Z. Chen, Optimized DLP 3D-printed high-resolution nano zirconia-hydroxyapatite scaffold with craniomaxillofacial soft tissue invasion resistance and pro-osteogenic properties via dectin-1/syk inflammatory axis, Chem. Eng. J. 491 (2024) 152044. https://doi.org/10.1016/j.cej.2024.152044.

[69] H. Chen, W.-L. Lo, S.-Y. Lee, Y.-M. Lin, Controlling sintering temperature for biphasic calcium phosphate scaffolds using submicron hydroxyapatite slurries for LCD 3D printing, Ceram. Int. 50 (2024) 11060–11074. https://doi.org/10.1016/j.ceramint.2024.01.007.

[70] H. Zhu, M. Li, X. Huang, D. Qi, L.P. Nogueira, X. Yuan, W. Liu, Z. Lei, J. Jiang, H. Dai, J. Xiao, 3D printed tricalcium phosphate-bioglass scaffold with gyroid structure enhance bone ingrowth in challenging bone defect treatment, Appl. Mater. Today 25 (2021) 101166. https://doi.org/10.1016/j.apmt.2021.101166.
